# Advancements in supercapacitors: breaking barriers and enabling amazing applications

**DOI:** 10.1039/d5sc01955a

**Published:** 2025-05-15

**Authors:** Sidhanath V. Bhosale, Sheshanath V. Bhosale

**Affiliations:** a Polymers and Functional Materials Division, CSIR-Indian Institute of Chemical Technology Hyderabad-500007 Telangana India bhosale@iict.res.in; b Department of Chemistry, School of Chemical Sciences, Central University of Karnataka Kadaganchi Kalaburagi-585 367 Karnataka India bsheshanath@cuk.ac.in

## Abstract

Supercapacitors (SCs) display intrinsic advantages such as high power density and high rate capability but low energy density. Thus, the development of advanced pseudocapacitive electrode materials is crucial for the advancement of supercapacitor technologies. These electrode materials significantly influence the performance of supercapacitors in electrical energy storage (EES) systems in terms of energy density and cycling stability. In this review, we first discuss EES technologies and their development and types of SCs, followed by an overview of the importance of organic electrode materials in pseudocapacitor (PSC) applications. Moreover, we present the principles of different redox-active organic molecule design strategies and their theoretical calculations to understand their electrochemical characteristics. Furthermore, we highlight the role of redox-active organic electrode materials in achieving a wider potential voltage window and, in turn, higher energy density, thus enhancing the electrochemical performance of PSCs. Subsequently, we discuss the role of molecular structures, the composition of electronic conducting materials and their structural and electrochemical performance relationship. Moreover, we highlight the advantages and disadvantages of organic materials compared with traditional transition-metal oxide inorganic materials for PSCs. Then, we present a brief discussion on the advances in small redox-active molecular architectures and their use in the fabrication of novel electrode materials, including polymers, covalent organic frameworks and metal organic frameworks. We provide an in-depth discussion on how material development from small redox-active molecules advances the charge-storage field and their application in illuminating light-emitting diodes. We hope that this review article will help provide a fundamental basis for the design and development of next-generation pseudocapacitive electrode materials from renewable sources for sustainable supercapacitor systems with higher charge-storage capability.

## Abstract 2

The present review article provides a way to design high-performance energy storage materials based on organic compounds for flexible electronics and wearable device architectures.

## Graphical abstract

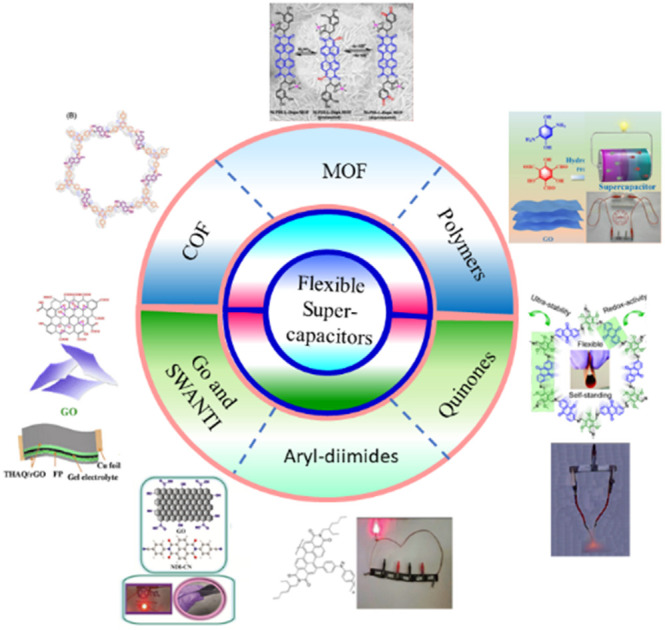

## 1. Introduction

In modern society, the exponential increase in the use of fossil fuels has led to significant environmental consequences, resulting in global warming and significant climate change.^1^ Thus, to avoid further environmental pollution, it is necessary to combine renewable energy resources with advanced technology development for energy generation, energy storage and conservation. To tackle the challenges in these fields, electrical energy storage (EES) technologies have been developed for achieving power storage and supply on various scales. Some EES technologies include batteries and fuel cells, which are used in daily life, whereas supercapacitors (SCs) are employed in industrial applications. Therefore, researchers around the world are working on different systems to reduce the use of fossil fuel. The contribution of non-renewable energy sources can be effectively reduced through the shift towards renewable energy sources.^2^ To reduce the environmental pollution, it is necessary to develop efficient power conversion technologies using the renewable energy sources. In this case, the storage of renewable energy generated on a mega scale, such as solar energy,^3^ nuclear energy,^4^ ocean energy^5^ and biomass energy,^6^ and its transfer to grids^7^ are challenging tasks for researchers. Presently, to achieve this task, the development of proper energy storage systems is necessary.^8^ In this connection, the development of flexible high-capacity energy storage technologies for power generation and storage from renewable energy sources is urgent.^9^ In addition, energy storage devices should ensure the supply-demand balance with promising consistency.^10^ To fulfil the need for power storage and supply on different scales, a number of electrical energy storage (EES) systems have been developed.^11^ EES technologies can store the generated energy for later use. EES systems such as flywheel energy storage (FES), compressed air energy storage (CAES), pumped hydroelectric storage (PHS), superconducting magnetic energy storage and supercapacitors are utilized in industrial applications, whereas batteries and fuel cells are employed in daily life.^12,13^ Among these technologies, electrochemical storage such as batteries [battery energy storage (BES)] have been widely utilized due to their efficiency in terms of lifetime, discharge time, scalability, and weight and/or mobility in devices.^14^ Batteries are broadly classified into two types, rechargeable and non-rechargeable. Both types of batteries generate electricity from chemical energy *via* redox-reactions between their electrodes, *i.e.* anode and cathode *via* the electrolyte. The former type of batteries cannot be recharged, whereas in rechargeable batteries, the process can be reversed and the raw-materials are re-formed.^15,16^ Presently, rechargeable batteries are the most common type of batteries utilized in portable electronic devices due to their high energy density and higher efficiency.^17^ However, battery technologies that can be applied in advanced equipment on various scales are limited not only due to their low power density, heat generation and limited cycle life performance but also their cost.^16,18,19^ Thus, to overcome all these aspects and provide a full solution for electrical energy storage applications, well-developed, durable, cost-effective, reliable devices with higher cycling stability and high energy and power densities are required. Therefore, the development of EES devices has become a major area of research.^20,21^ In this case, the suitable combination of various EES systems can be utilized to meet technical requirements, lower the cost of the device, increase safety and consumer friendly nature and achieve higher efficiency and long-term cycling life to transform the landscape of energy storage and distribution.^22^ Accordingly, to fulfill the energy storage demand in combination with battery systems, supercapacitor technology, which can display a higher electrochemical performance such as higher charge storage, higher power density and longer cycling stability, can provide a better solution for different power systems.^23^ To achieve these properties, the electrolytes and electrode materials in supercapacitors play key roles.^24,25^

In this review, we emphasize the recent progress on the material design and chemistry of supercapacitor electrodes, and their effect on the energy storage performance of devices is summarized and compared. More importantly, the advantages and disadvantages of organic electrode materials in comparison to conventional inorganic materials, advanced characterization and theoretical calculations to understand the mechanisms of various organic materials will be demonstrated, together with the present status, perspectives and challenges regarding further improvements. We conclude with remarks on the challenges and prospectus to develop pseudocapacitive materials for advanced SC applications.

## 2. Supercapacitors

Supercapacitors (SCs)^26^ are considered energy storage devices that display an electrochemical performance between that of traditional capacitors^27,28^ and batteries,^29^ satisfying the ever increasing demand for providing power in electronics and industrial applications. General Electric fabricated and patented the first supercapacitor in 1957.^30^ SCs exhibit specific capacitances a million times higher than that of traditional capacitors.^26^ Moreover, the cycling life^27^ of SCs is very long and they display stability over a wide operating temperature range.^31^ It is noticeable that the energy and power densities of SCs are between that of conventional capacitors and rechargeable batteries.^26–29^ However, despite the attractive features and applications in industry, military, electronics and transportation, they are associated with some shortcomings compared to capacitors and batteries. Among the limitations exhibited by SCs, the main difficulty is to improve their energy density.^32^ The lower density displayed by SCs results in bulkier cell configurations. Secondly, it is very important to have an ideal supercapacitor model to investigate the load nature, external environment and accidental risk of the cell and its stability in power supply applications of satellites to reduce the potential risks due to nonideal parameters.^33^ The third limitation of SCs is related to their very low voltage of <2.7 V, and therefore for practical applications, supercapacitors in series connections are required. Thus, in this field, it is necessary to develop industrial, national and international standard supercapacitors. Furthermore, to improve the practical utilization of supercapacitors in compact electronic appliances and industrial applications, it is essential to improve their manufacturing process and technology.^34^ Notably, it is important to develop new electrode materials and electrolytes to achieve higher energy density, which can be helpful to avoid connecting SCs in series.

## 3. Types of supercapacitors

The supercapacitor families^35^ are classified into three types, (i) electrical-double layer capacitors (EDLCs),^36^ (ii) pseudocapacitors^37^ and (iii) hybrid capacitors.^38^ The working mechanism of the EDLC cell configuration is to store charge electrostatically or *via* non-faradaic processes.^36^ Alternatively, pseudocapacitor (PSC)^37^ devices store charge *via* faradaic reversible redox reactions between the electrodes and electrolyte. This process relies on the transfer of electrical charges through redox reactions. The third, type, hybrid supercapacitors,^38^ are a combination of EDLCs and PSCs. These hybrid devices store charges *via* non-faradaic processes and the faradaic reversible redox-processes. Thus, hybrid SCs with low cost and better electrochemical performance in a wider temperature range, longer cycling life, low resistance and higher energy and power densities are utilized in vehicles.^39^ Although the progress of the above-mentioned supercapacitors has reached its peak in different applications, it is still necessary to improve the performances of the SC cell configurations by designing suitable materials and fabricating compact device configurations.^40^ The challenges in the fabrication of next-generation SCs include low energy density, poor cycling stability and low electrochemical performance. Thus, to tackle these challenges, the development of high-performing electrode materials with a wider applied potential voltage window is crucial, which will achieve a higher energy density, and thus improved electrochemical charge storage capacity and higher *C*_sp_.^41^ In this case, researchers have devoted their efforts to developing novel electrode materials based on renewable organic materials with high surface areas and abundant redox-active sites, and also incorporating suitable donor and acceptor entities, which can endure a wider potential window. By addressing these issues, researchers can achieve SC devices with higher energy densities, which will become competitive alternatives to batteries. These SCs aim to provide solutions to the supercapacitor industry to achieve environmentally friendly low-cost electrode materials to promote the healthy development of the next generation industry.

## 4. Organic electrode materials for supercapacitor applications

Owing to the advances in supercapacitors, they are emerging as the most efficient charge storage technology. Presently, SCs play an important role in daily life to fulfill the modern energy demand. They display applications in various fields including portable electronic devices,^42^ electrical vehicle applications,^43^ wearable and biointegrated electronics,^44^ microgrid^45^ and defence applications.^46^ Among the supercapacitors, pseudocapacitors play a critical role, showing a promising and sustainable future.^47^ The important components in the pseudocapacitor structure are electrodes.^37,47^ The conventional inorganic metal oxide,^48*a*^ conducting polymer, *e.g.* polyaniline (PANI) and polypyrrole (PPy),^48*b*,*c*^ active-carbon^49^ and MXene^50^ electrode materials are employed in PSCs^51^ for delivering spectacular electrochemical performances in the cell configuration. However, the metal oxide electrode materials rely on non-renewable sources of metals, are expensive and approaching their performance limits with respect to electrical conductivity and power density. Alternatively, PANI and PPy are cost-effective, and PSCs based on these materials exhibit good conductivity, easy processability and higher energy density but displayed some limitations such as lower cycling stability during electrochemical processes. Active-carbon-based electrode materials display excellent performances due to their higher surface area, chemical and thermal stability and very low electrical resistance. However, in real-world applications they display limitations due to their lower energy density. In recent years, MXenes have emerged as attractive alternatives but their limited potential voltage window results in a lower energy density. Therefore, to sustain the pace of technology with economic viability and high energy storage performances, the design and synthesis of new electrode materials for the fabrication of next-generation pseudocapacitors with tunable chemical properties, thermal stability, environmentally sustainable nature and high atom economy are essential.^52,53^ Furthermore, to develop next-generation pseudocapacitors, new design strategies are needed based on renewable resources.^54^ Herein, organic electrode materials (OEMs) utilizing redox-active small molecules and polymers have attracted attention from researcher due to their economic viability, versatility, ease of chemical structural modification, flexibility, environmental sustainability and higher electrochemical performance.^52–54^ These molecular and polymeric entities have abundant natural sources and hold promise for sustainable development.^55^ To fabricate efficient organic electrode materials for PSC applications, organic compounds need to satisfy the following criteria: (i) exhibit redox-reversibility during electrochemical processes, (ii) incorporate the electrolyte ions during the reversible process, (iii) insoluble in the electrolyte solution, and (iv) ability store and release charges on demand.^56^ However, although OEMs have displayed improved performances, they exhibit several challenges such as unfavourable potential voltage window, interaction with neighboring redox-active subunits, solubility in the electrolyte and practical solubility issues.^52–56^

In the present review, we aim to address the progress and challenges of redox-active electrode materials compared to inorganic transition metal oxides, conducting polymers, active-carbon materials and MXenes. Moreover, our focus is gaining a better understanding of their molecular design using theoretical approaches, synthesis, redox properties, voltage profiles and electrochemical performance for PSC applications.

### 4.1. Benzoquinone, naphthoquinone and anthraquinone for supercapacitors

#### 4.1.1. Benzoquinone for SC applications

Quinones play an important role in harvesting photosynthetic electrons from *Chlamydomonas reinhardtii*, a green unicellular alga, and generating a steady-state photocurrent.^57^ In the natural photosystem II (PSII), quinones (Q) acts as acceptors and are utilized as redox mediators.^58^ Under sunlight illumination, quinone undergoes reduction, resulting in the formation of hydroquinone (QH_2_) in the photosynthetic electron transfer process. Subsequently, obtained QH_2_ undergoes oxidation, resulting in a quasi-steady state (eqn (1)).^59^

1 QH_2_ = Q + 2e^−^ + 2H^+^

**Scheme 1:**
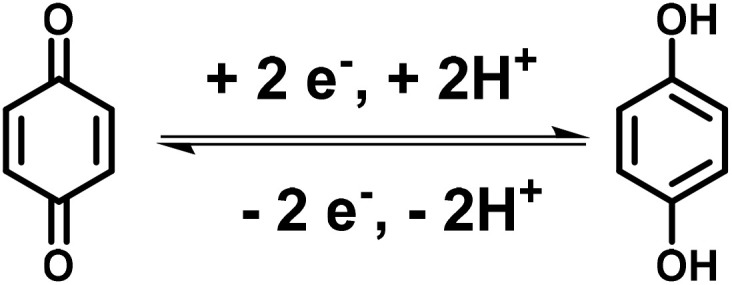
Reversible redox-reactions of quinone during the electrochemical process.

Thus, quinone bearing two carbonyl groups undergoes redox reactions involving two electrons and two protons.^60^ Moreover, the redox chemistry of quinone in aqueous electrolyte was established, which showed its electrochemical reversibility at lower pH.^61^ The faradaic reversible-redox process displayed by quinones makes them attractive candidatures for pseudocapacitor applications.^62^ Blanco and co-workers demonstrated the charge storage properties of a carbon-based supercapacitor functionalized by the redox-active quinone (Q)/hydroquinone (HQ) in the aqueous H_2_SO_4_ electrolyte (Scheme 1).^63^ The as-fabricated electrode materials in a three-electrode supercapacitor system as the anode and cathode displayed an outstanding specific capacitance (*C*_sp_) of 5017 F g^−1^ and 477 F g^−1^, respectively. The obtained *C*_sp_ values were superior to that of the original carbon-based supercapacitors, which showed the value of ∼290 F g^−1^*via* double-layer formation. The higher *C*_sp_ value was attributed to the slower kinetics displayed through the Q/HQ reversible redox-reactions, which was due to the major faradaic reversible redox reactions at a lower current density, indicating the pseudocapacitive behavior of the device. Moreover, the energy density of the SC device was found to be 30.6 W h kg^−1^, which is higher than that of the original carbon-based SC (10.1 W h kg^−1^).

It is noticeable that before the utilization of quinone as an electrode material in PSCs, Trasatti and co-workers developed the first inorganic transition metal oxide, *i.e.* RuO_2_, based electrode materials in H_2_SO_4_ electrolyte for pseudocapacitive charge storage applications.^64^ In this case, when RuO_2_ in its hydrous form in H_2_SO_4_ electrolyte was used as an electrode in SC devices, it showed *C*_sp_ of about 720 F g^−1^ at a scan rate of 2 mV s^−1^ and energy density of 26.7 W h kg^−1^.^65^ Herein, we conclude that compared to RuO_2_-based^65^ inorganic counterpart (*C*_sp_ 720 F g^−1^, 26.7 W h kg^−1^), the organic molecule quinone-based^63^ (*C*_sp_ 5017 F g^−1^, 30.6 W h kg^−1^) electrode in the PSC device exhibited a higher *C*_sp_ and energy density in aqueous H_2_SO_4_ electrolyte. The performance of the quinone moiety relies on reversible redox-reactions in acidic electrolyte. The price of benzoquinone is about Rs. 3000 per kg (Indian currency in 2025), which is much cheaper than RuO_2_ (about Indian Rs. 6480 per g in 2025). Thus, the lower cost and outstanding electrochemical properties of quinone compared to ruthenium oxide make it an attractive electrode material for commercialization in electrochemical energy storage devices.

**Fig. 1:**
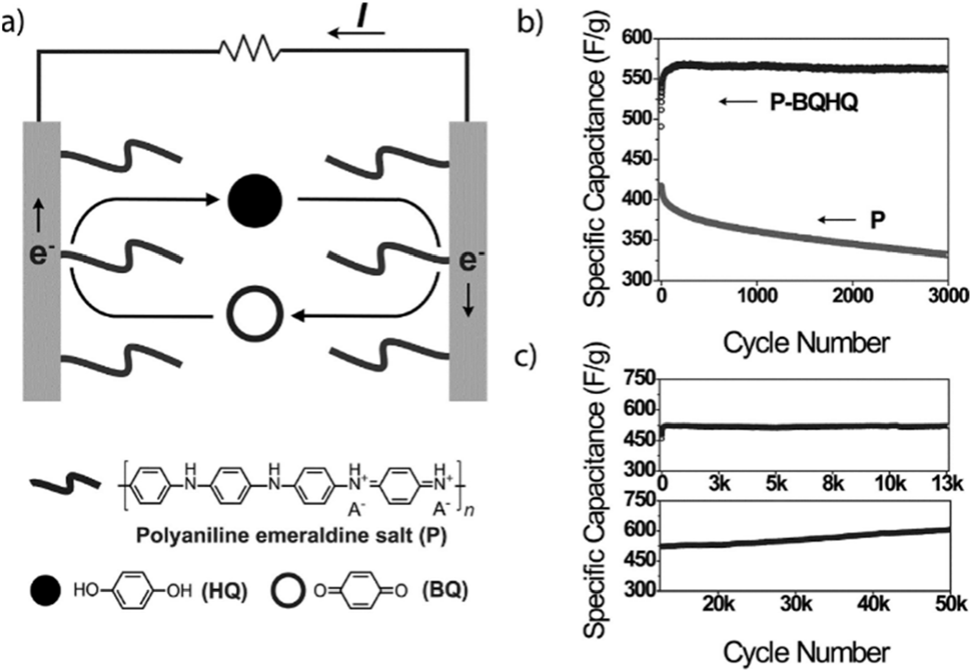
(a) SC with a polyaniline salt form-based electrode and Pt-metal current collectors in quinone electrolyte and a supporting electrolyte (H_2_SO_4_/AcOH); (b) *C*_sp_ of the SC *vs.* cycle number from GCD in BQHQ/H_2_SO_4_/AcOH electrolyte solution (P-BQHQ, black curve) and in H_2_SO_4_/AcOH (P, grey curve). (c) Cycling stability of P-BQHQ SC over 50 000 GCD cycles. Reproduced from ref. 67 with permission from [John Wiley and Sons], Copyright [2014].

In recent years, owing to the low cost and fast reversible redox kinetics of quinone, it has emerged as an attractive electrolyte and electrode material, and thus immensely explored for pseudocapacitor applications. Santamaría and co-workers demonstrated the utilization of redox-active Q/HQ as the supporting electrolyte, which enhanced the *C*_sp_ to 901 F g^−1^ at 2.65 mA cm^−2^ for the SC device based on an activated carbon electrode.^66^ This value is higher than that of SCs (720 F g^−1^) based on a ruthenium electrode.^65^ The contribution of redox-active organic scaffolds as an electrolyte resulted in the large *C*_sp_. In addition, in 2014, Heeger and co-workers reported that the influence of the BQ/HQ redox electrolyte enhanced the electrochemical performance of an SC device (Fig. 1) based on polymeric electrodes (P-BQHQ) in H_2_SO_4_/AcOH electrolyte, exhibiting *C*_sp_ of 524 F g^−1^ after 200 cycles (Fig. 1b).^67^ The SC device displayed long-term cycling stability over >50 000 cycles (Fig. 1c). The *C*_sp_ of a polymer-based device increased in the presence of BQHQ electrolyte by a factor of 5.5 compared to the bare polymer-based SC device, reaching 2646 F g^−1^ at a current density of 0.5 mA cm^−2^. The *C*_sp_ of the thicker polymer in the presence of the supporting BQHQ electrolyte was almost doubled to 882 F g^−1^. The increase in electrochemical performance is ascribed to the faster reversible redox kinetics between the polymeric electrode and the BQHQ electrolyte. Herein, the authors attributed to the stability of the device to the lower pH created by the doped conducting polymer.

**Fig. 2:**
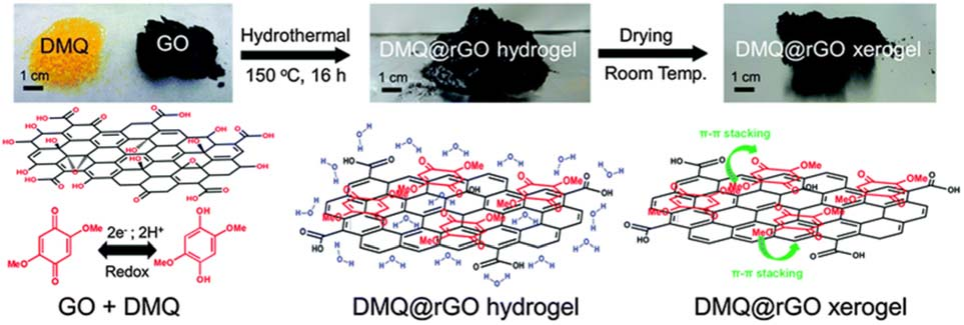
Schematic presentation of DMQ@rGO xerogel electrode material preparation. Reproduced from ref. 68, with permission from RSC.

In 2016, the Gogotsi group reported that a quinone derivative such as 2,5-dimethoxy-1,4-benzoquinone (DMQ)-modified reduced graphene oxide (rGO) (denoted as DMQ@rGO electrode) (Fig. 2) acted as a pseudocapacitive electrode in 1 M sulfuric acid and displayed an excellent *C*_sp_ of 650 F g^−1^ at a scan rate of 5 mV s^−1^.^68^ Moreover, at 50 mV s^−1^, the electrode exhibited excellent *C*_sp_ retention of 99% after 25 000 cycles. The obtained *C*_sp_ shows promise compared to numerous inorganic electrodes.

**Fig. 3:**
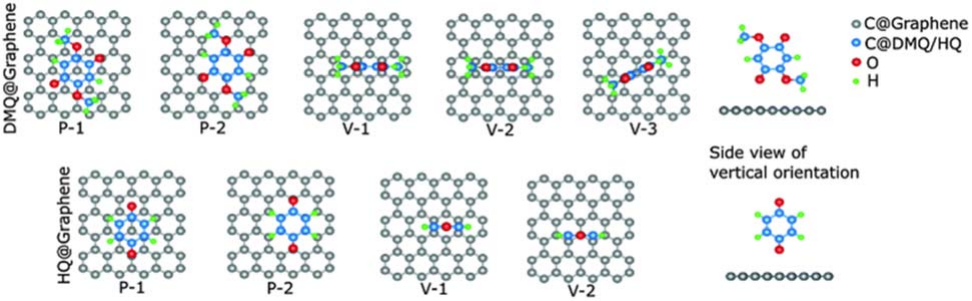
Optimized geometries of DMQ and HQ on graphene surface according to DFT calculations. Reproduced from ref. 68 with permission from RSC.

The authors utilized density functional theory (DFT) calculations to understand the energy storage mechanism of the device. Initially, they examined the preferred adsorption orientation of DMQ and HQ on the graphene sheets, followed by estimation of their binding energies in different orientations during the energy storage process using eqn (2) (Fig. 3 and Table 1).^68^

2 *E*_b_ = *E*_graphene+molecule_ − (*E*_graphene_ + *E*_molecule_)

where *E* is the total energy of the respective components.

**Table 1:** Adsorption energies (eV) of DMQ and HQ on the graphene (gr.) surface in different orientations

Composite	P-1	P-2	V-1	V-2	V-3
DMQ@gr.	−1.36	−1.25	−0.59	−0.68	−0.66
HQ@gr.	−1.01	−0.98	−0.46	−0.45	

The estimated energies for parallel adsorption such as the DMQ@gr. (P-1) and HQ@gr. (P-1) states were found to be −1.36 eV and −1.01 eV, respectively (Table 1). This type of π–π-stacking interaction between DMQ and HQ on the graphene surface implies that the maximum charge transfer process happens in the P-1 state. In addition, the higher binding energy of the DMQ@gr. composite compared to HQ@gr. indicates that the former displays a more stable cycling performance than the latter composite during electrochemical processes.

**Fig. 4:**
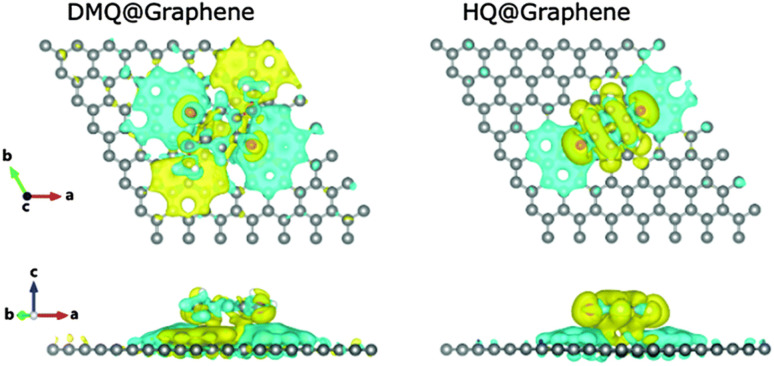
DMQ@gr. and HQ@gr. composite materials in stacked sites and their charge density differences. Turquoise and yellow regions suggest depletion and accumulation of electrons, respectively. Reproduced from ref. 68 with permission from RSC.

According to the charge density calculations, the authors demonstrated the most stable configurations between the DMQ@gr. and HQ@gr. composite materials.^68^ As shown in Fig. 4, DMQ causes a larger charge distribution on the graphene surface compared to HQ. This results in larger electrostatic interaction between DMQ and the graphene surface, enhancing the *C*_sp_ and cycling performance of the SC device. Herein, the charge distribution shown in Fig. 4 also implies that not only the carbonyl functional group but also the methoxy moiety of DMQ take part in electrochemical processes. Thus, based on the theoretical and experimental results, the authors claimed that the stronger adhesion of DMQ on the graphene surface will lead to a greater charge distribution, leading to a longer cycle life.

**Fig. 5:**
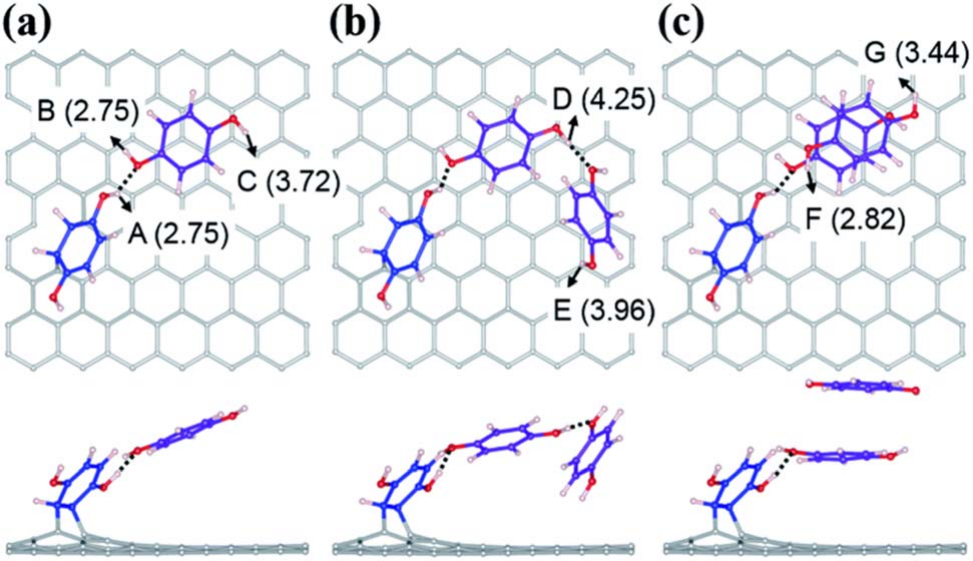
Different HQ structures formed on the carbon surface. (a) Hydrogen-bonded HQ molecule directly grafted on surface; (b) two hydrogen-bonded HQ molecules indirectly grafted; (c) hydrogen bond-*co*-π–π stacking two HQ molecules form a hydrogen bonding with a directly grafted HQ. *Directly grafted HQ benzene rings are marked in blue, whereas indirectly grafted HQs are marked in purple. The hydrogen bonds are represented by a dashed line. Reproduced from ref. 69 with permission from RSC.

**Fig. 6:**
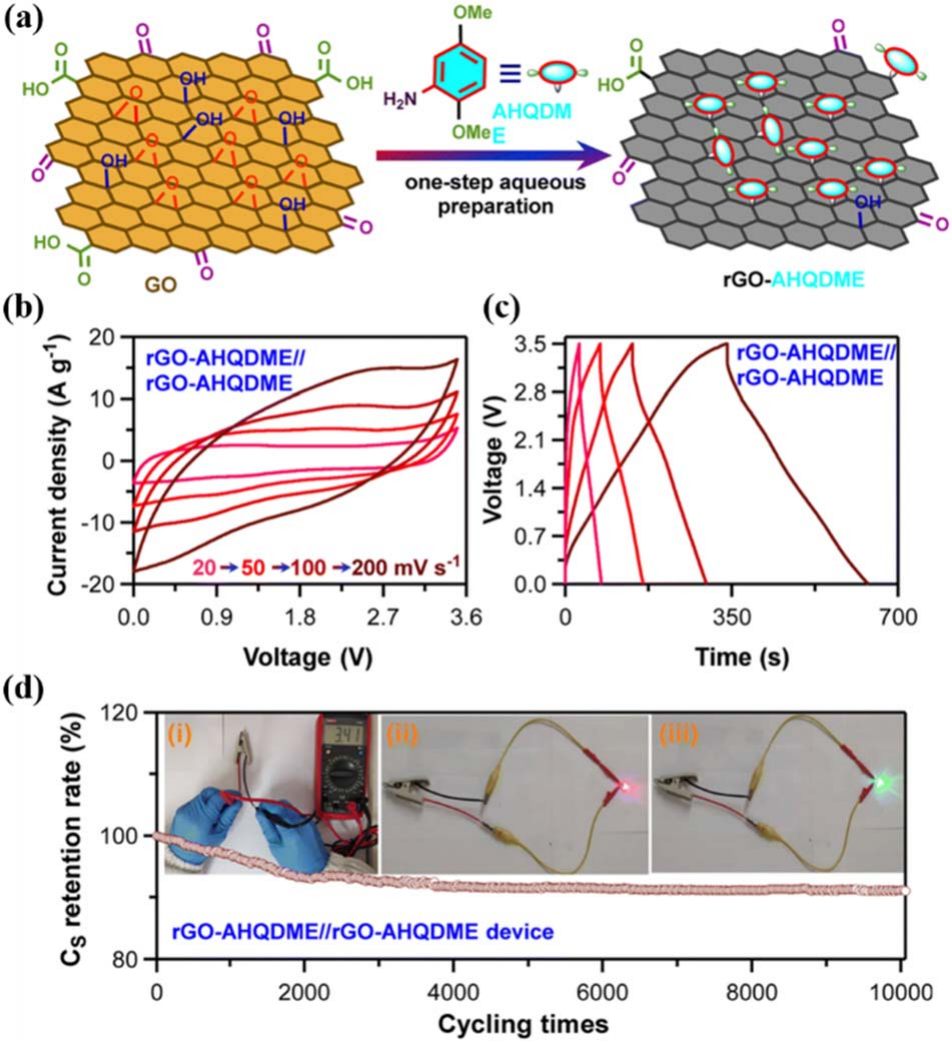
(a) Schematic presentation of the preparation of the rGO-AHQDME composite material; (b) CV of rGO-AHQDME//rGO-AHQDME at various scan rates; (c) GCD of rGO-AHQDME//rGO-AHQDME at various current densities and (d) cycling stability and illumination of LED light at 3.5 V. Reproduced from ref. 73 with permission from RSC.

Kim and co-workers demonstrated the use of redox-active hydroquinone (HQ) pseudocapacitor materials in a high-performance flow capacitor (HFC) application.^69^ The device exhibited the maximum *C*_sp_ of 513 F g^−1^ and energy density of ∼14 W h kg^−1^ with a 0.38 M HQ redox-mediator slurry electrode.^69*a*^ It has been well documented that the capacitive performance of the electrode also depends on the pore size and changes in the texture of activated carbon.^69*b*,*c*^ The specific surface area (SSA), pore size distribution (PSD) and pore volume parameters are utilized to describe the pore texture of carbon materials. The anchoring of the redox-active HQ influences the microporous structure of carbon materials, which can be examined using the Brunauer–Emmett–Teller (BET) surface area and PSD. These authors measured the N_2_ adsorption/desorption isotherms of carbon spheres, carbon black and 1 M H_2_SO_4_ as double-layer capacitive slurry and pseudocapacitive slurry electrodes, together with 0.3 M HQ. The N_2_ adsorption/desorption isotherms of the cycled double-layer capacitive and pseudocapacitive slurry are displayed in Fig. 6a. The estimated BET surface areas using the N_2_ adsorption/desorption isotherms were found to be 1948 m^2^ g^−1^ and 1411 m^2^ g^−1^ for the double-layer capacitive and pseudocapacitive slurry, respectively, suggesting a decrease in surface area following the incorporation of 0.3 M HQ molecules. Fig. 6b displays the pore size distributions of the cycled double-layer capacitive and pseudocapacitive slurries. It was confirmed that upon the addition of 0.3 M HQ, a reduction in pore size and PSD broadening occurred. The specific capacitance of the SC device using the pseudocapacitive slurry electrode in the presence of 0.3 M HQ reached the maximum and decreases at an HQ concentration of 0.38 M. The authors claimed that the decrease in the double-layer capacitance could be attributed to the pore size constriction with an increase in the HQ loading. Moreover, the presence of HQ molecules in the electrolyte resulted in the formation of clusters *via* hydrogen bonding on the pore entrance during the charge/discharge cycles. Therefore, the increase in the blockage of the pores happened in the presence of the grafted HQ and their clusters, which obstructed the ion mobility. This led to a reduced double layer-capacitance. These results are superior to that for previously reported slurry electrodes for aqueous FCs. They also performed DFT calculations^70,71^ to understand the basis for the enhanced electrochemical performance with the grafting of HQ on the activated carbon surface. As shown in Fig. 5, different HQ structures grafted on graphene sheets were utilized to display the amorphous carbon surface model. The estimated desorption energy (*E*_v_) values for the examined model were in the range of 2.75–4.25 eV for the various –OH sites. In case A, the directly grafted HQ on the graphene surface displays an energy of 2.75 eV, whereas, in the case of indirectly grafted HQ, *i.e.* B–G, the estimated energies are larger than that in case A. According to the experimental CV results, the authors found that cases A and B displayed peaks at 0.1 V and C–G cases exhibited peaks in the range of 0.2 to 0.7 V in their CV profiles. According to these results, it was indicated that the HQ redox-active molecular scaffolds grafted directly or indirectly on the graphene surface contribute to the specific capacitance through faradaic reversible redox-reactions. They found that the HQ slurry participated to enhance the *C*_sp_ by about two times. Moreover, the presence of neighbouring water on the H distortion decreases the *E*_v_ values but does not change the reaction energies quantitatively.

Lv and co-workers reported the preparation of tetraamino-benzoquinone (TABQ)-modified carbon nanotube (MWCNTs) electrode materials (TABQ-MWCNTs) for PSC applications.^72^ In a three-electrode SC system, the fabricated electrode displayed a *C*_sp_ of 463 F g^−1^ at 1 A g^−1^. The adsorption/desorption isotherms of MWCNTs and TABQ-MWCNTs (4 : 1) were recorded. These electrode materials exhibited mesoporous characteristics. The estimated SSA of MWCNTs and TABQ-MWCNTs (4 : 1) was found to be 115.64 and 62.85 m^2^ g^−1^, respectively. TABQ-MWCNTs displayed a decrease in SSA, indicating the partial coverage of their surface due to the adoption of TABQ on the surface of MWCNTs. At a current density of 1 A g^−1^, the calculated *C*_sp_ was found to be 7.4, 17, 185, 215, 278, 463, and 291 F g^−1^ for the pure TABQ, bare MWCNTs, TABQ-MWCNTs (1 : 1), TABQ-MWCNTs (2 : 1), TABQ-MWCNTs (3 : 1), TABQ-MWCNTs (4 : 1), and TABQ-MWCNTs (5 : 1), respectively. It was observed that TABQ-MWCNTs (4 : 1) exhibited the highest *C*_sp_ of 463 F g^−1^ among the tested electrodes with different mass ratios, where the MWCNT surface was fully covered with TABQ molecules. The lower mass ratio of TABQ in 1 : 1, 1 : 2 and 1 : 3 TABQ-MWCNTs resulted in a lower *C*_sp_, which could be attributed to the low conductivity of the TABQ organic materials. At a higher mass ratio in TABQ-MWCNTs (5 : 1), a decrease in specific capacitance was observed, which could be ascribed to the excessive presence of TABQ blocking the surface of MWCNTs. This reduced the contribution of the TABQ-MWCNT composite active sites to the resulting *C*_sp_. Moreover, the SC device exhibited 76.8% *C*_sp_ retention of its initial value after 6000 cycles at a current density of 10 A g^−1^.^72^ The TABQ-MWCNT electrode in the asymmetric two-electrode TABQ-MWCNT//activated carbon SC device showed a specific capacity of 57.3 F g^−1^ at 1 A g^−1^ and energy density 15.6 W h kg^−1^ at a power density of 700 W kg^−1^. At 5 A g^−1^, the ASC device exhibited 91.5% *C*_sp_ retention after 10 000 cycles. Herein, TABQ can be easily grafted on the surface of MWCNTs *via* π–π stacking interactions. Furthermore, the structural stability of the composite originated from the non-covalent hydrogen-bonding between the hydroxyl groups present in the MWCNT structure and the amino functional group of TBAQ. This stability provided faster electron transportation pathways, facilitating the charge storage process in the TABQ-MWCNT composite electrode. The higher *C*_sp_ could be attributed to the synergistic effect generated by the TABQ organic scaffold and MWCNTs conducting material. In 2023, Shen and co-workers reported the fabrication of an amino hydroquinone dimethylether (AHQDME)-functionalized reduced graphene oxide (rGO) electrode (Fig. 6) and its charge storage properties.^73^ In the proto-type three-electrode SC device, the rGO-AHQDME electrode displayed a *C*_sp_ value of 523 F g^−1^ at a current density of 1.0 A g^−1^, which is close to the theoretical capacitance value of pristine graphene of about 550 F g^−1^. The symmetric two-electrode rGO-AHQDME//rGO-AHQDME SSC device in acetonitrile (AN) and EMIMBF_4_ (1 : 1, mass ratio) electrolyte exhibited *C*_sp_ of 338 F g^−1^/100% at 1.0 A g^−1^ and an energy density as high as 143 W h kg^−1^ at a power density of 1745 W kg^−1^ (Fig. 6b and c, respectively). The present protocol represents a simple way to fabricate practical rGO materials for constructing high energy density SCs. The SSC device showed excellent *C*_sp_ retention of 91% after 10 000 GCD cycles, and was also successfully utilized to illuminate an LED light with a voltage of 3.5 V (Fig. 6d). The obtained results for the AHQDME organic material on rGO surface-based electrode in the SC and SSC cell configurations are superior to that of an MnO_2_-based spinel nanostructure electrode material, exhibiting *C*_sp_ of 241 F g^−1^ with pseudocapacitive behaviour.^74^

**Fig. 7:**
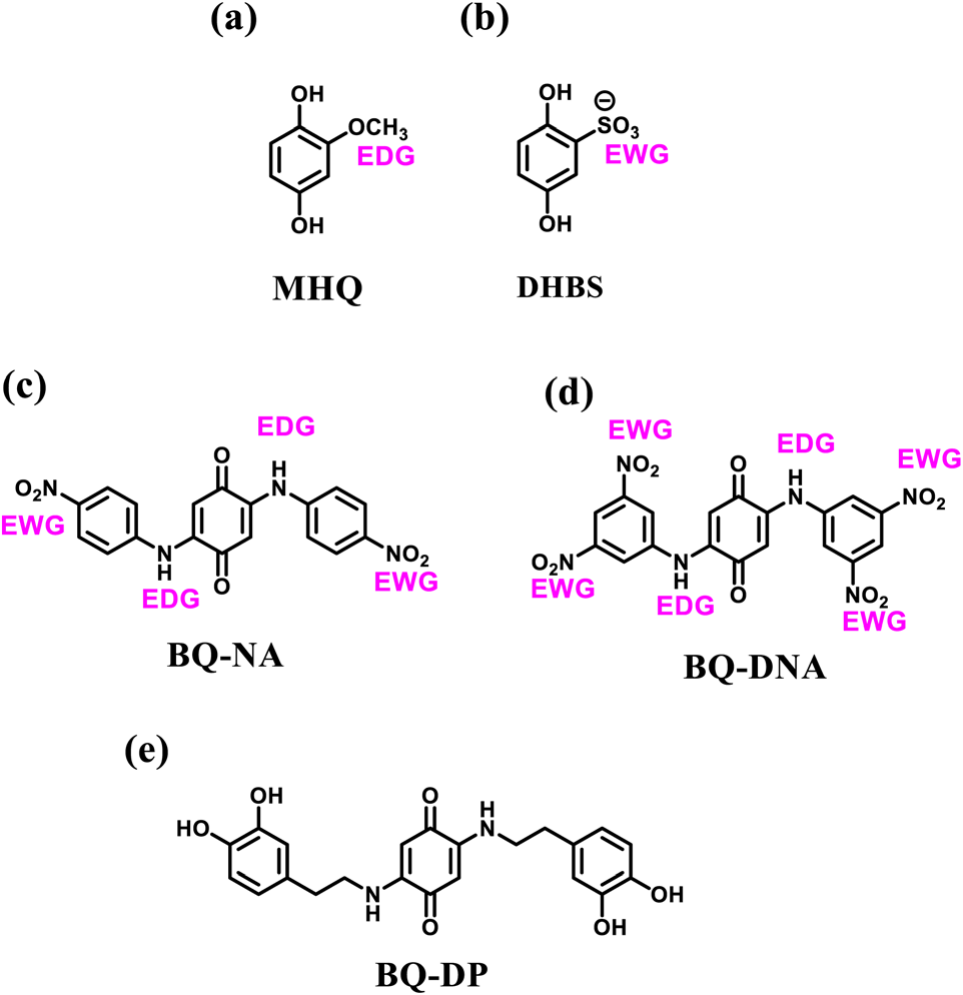
Functionalized hydroquinone and quinone: (a) MHQ, (b) DHBS, (c) BQ-NA, (d) BQ-DNA and (e) BQ-DP for supercapacitor applications.

Biradar *et al.* demonstrated the preparation of an adenine-functionalized quinone pillared graphene oxide system for three-electrode SC and two-electrode SSC applications.^75^ The calculated *C*_sp_ of ABQA-GO/CP//ABQA-GO/CP was 134 F g^−1^ with an energy density of 32.87 W h kg^−1^ at a power density of 1256 W kg^−1^ at 0.5 A g^−1^. To achieve superior charge storage properties, researchers modified the core structure of quinone and hydroquinone with suitable functional groups as well as organic subunits (Fig. 7). In this regard, in 2018, Zhang and co-workers utilized electron-donating groups (EDG) such as methoxyl and electron-withdrawing groups (EDG), *e.g.* sulfonic acid, to modify the *p*-hydroquinone (PHQ).^76^ The obtained molecular structures were 2-methoxyhydroquinone (MHQ) (Fig. 7a) and 2,5-dihydroxybenzenesulfonate (DHBS) (Fig. 7b). It was demonstrated that the PHQ, DHBS and MHQ molecular entities can act as effective redox-additives in the presence of aqueous 1 M H_2_SO_4_ electrolyte. In a two-electrode SSC system, the DHBS-2 (2 mmol L^−1^) sample exhibited the highest *C*_sp_ of 112 F g^−1^ at 1 A g^−1^, which is comparatively higher than that of MHQ-2 (88 F g^−1^) (2 mmol L^−1^) and nearly 2.95-times that of the pristine C-blank sample in the absence of redox-active additives. Moreover, DHBS and MHQ exerted pseudocapacitive behaviour during the electrochemical process. The higher charge storage capacity of DHBS than that of the MHQ could be ascribed to the higher electronegativity of the sulfonic group (3.1933) compared to methoxyl (2.8638). Thus, this work provides a basis for the influence of electron-withdrawing and donating group functionalization on the charge storage capacity of the PHQ derivatives. To explore the influence of the number of electron-withdrawing groups on the Q core, Bhosale and co-workers demonstrated the synthesis and use of 2-nitroaniline (NA) and 3,5-dinitro aniline (DNA)-functionalized benzoquinone (BQ) scaffolds, *e.g.* BQ-NA (Fig. 7c) and BQ-DNA (Fig. 7d), respectively, in charge storage applications.^77^

**Fig. 8:**
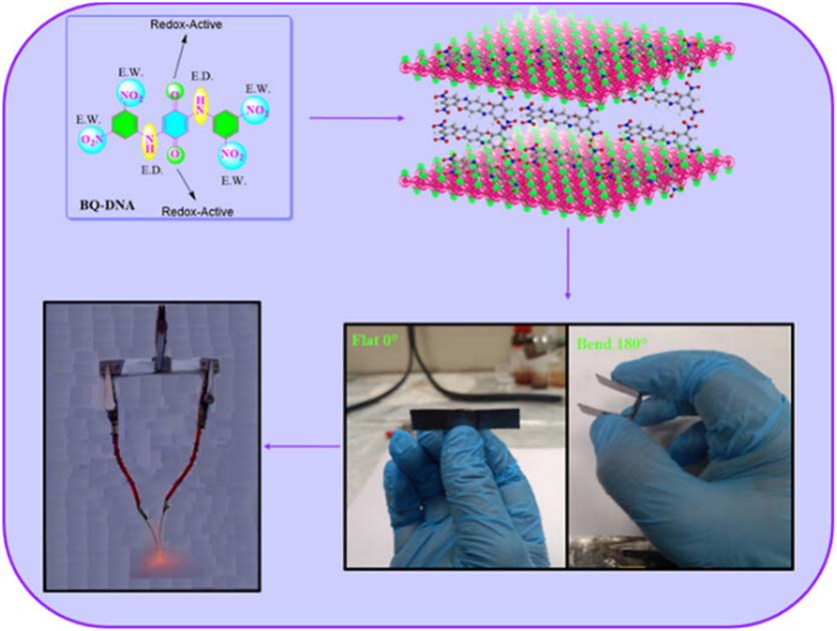
Schematic presentation of the molecular structure of BQ-DNA and the BQ-DNA/rGO composite, flexible device and illumination of LED light at 1.8 V. Reproduced from ref. 77 with permission from [the American Chemical Society], Copyright [2024].

The as-fabricated BQ-DNA/rGO electrode in a three-electrode SC system delivered a higher *C*_sp_ of 341.13 F g^−1^ compared to the BQ-NA/rGO (322.47 F g^−1^)-based device architecture. The larger *C*_sp_ of BQ-DNA/rGO could be attributed to its four EWG nitro functional groups. Moreover, the role of the pore size, volume and distribution of the electrode material in its charge-storage properties was examined by means of surface characteristics using BET analysis. The porous characteristics of the BQ-NA/rGO and BQ-DNA/rGO electrode materials were determined using the BET method and N_2_ adsorption–desorption measurements. The calculated SSA of BQ-NA/rGO and BQ-DNA/rGO was found to be 15.401 and 16.848 m^2^ g^−1^, respectively, suggesting that the latter displayed a higher surface area than the former electrode material. Further, the pore size distribution of BQ-NA/rGO and BQ-DNA/rGO was observed to be 16.66 and 13.051 nm, together with the estimated pore volumes of 0.064146 and 0.05497 m^3^ g^−1^, respectively. The larger surface area, higher pore size and sider adsorption pore size distribution of the electrode material can enhance its charge-storage properties. In addition, to examine the practical applications of the BQ-DNA/rGO composite electrode material, SSC and FSSC cell configurations were fabricated and investigated for their charge-storage properties (Fig. 8). The utilization of the BQ-DNA/rGO electrode for illuminating an LED light was performed at 1.8 V. The obtained *C*_sp_ and electron density results were impressive, paving the way for the construction of flexible wearable devices for next-generation SC applications using these materials.

The same group explored the energy storage properties of quinone functionalized with the neurotransmitter dopamine. The synthesized 2,5-bis((3,4-dihydroxyphenethyl)amino)cyclohexa-2,5-diene-1,4-dione molecule (BQ-DP) (Fig. 7e) organic material was utilized for the fabrication of a BQ-DP/carbon black (super P) (GS) electrode material.^78^ The BQ-DP/GS electrode was employed to investigate its charge storage properties in SC and SSC devices. The three-electrode SC cell displayed a significant specific capacitance. In addition, in the SSC device, the electrode delivered a *C*_sp_ of 247 F g^−1^ at 9.5 A g^−1^. Moreover, the SSC device with negligible EDLC contribution was treated as battery-type device, exhibiting a specific capacity of 115–58 mA h g^−1^ after 1000 cycles, and thus displayed a longer cycling life. The obtained results for the BQ-DP/GS electrode-based^78^ pseudocapacitor are superior compared to that of an MnO_2_-based electrode, which exhibited a *C*_sp_ of 529.5 F g^−1^ and 237.3 F g^−1^ at current densities of 1 A g^−1^ and 10 A g^−1^, respectively. Furthermore, the *C*_sp_ retention was found to be 79.8% after 2000 cycles.^79^ Thus, quinone and hydroquinone molecules upon functionalization with electron-withdrawing/donating groups as well as small redox-active molecules, *e.g.* dopamine, displayed excellent charge storage performances. These materials are available at a lower cost and the quinone core structure can be easily modified with suitable substituent groups. Furthermore, these molecular entities with a wider working voltage window can be utilized as cathode and anode materials. Thus, systems with quinone can play an important role in the next generation of hybrid symmetric SC applications.

To explore the PSC applications of redox-active quinone molecules, in recent years, some researchers have demonstrated the covalent grafting of organic molecular subunits on the carbon electrode surface. Herein, they presume that the pseudocapacitive properties of the redox-active organic molecular structure in combination with the carbon conductive network result in faster reaction kinetics and higher cycling stability.^80^ In 2023, Qiu and co-workers grafted the redox active *p*-benzoquinone (PBQ)-functionalized *p*-phenylenediamine (PPD) on the surface of microporous carbon materials *via* covalent bonding.^81^ The BET SSA of activated carbon (AC) was recorded to be 2084 m^2^ g^−1^. Upon grafting the surface of AC with organic materials, a dramatic decrease in the SSA of AC-PPD and AC-PPD-PBQ was observed to 643 m^2^ g^−1^ and 203 m^2^ g^−1^, respectively. The SSA results demonstrate the successful incorporation of the PPD and PBQ molecules in the AC microporous frameworks. The authors observed that the as-fabricated AC-PBQ displayed nearly the same SSA value as AC in the absence of the PPD molecular architecture, suggesting weak π–π stacking interactions between PBQ and the AC framework. The BET results implied that the PPD molecular subunit plays an important role as a covalent linker between PBQ and the surface of AC. Consequently, the as-fabricated AC-PPD-PBQ electrode displayed excellent charge storage characteristics with an extremely high *C*_sp_ of 377 F g^−1^ at 0.5 A g^−1^ and 276 F g^−1^ retention at a current density of 100 A g^−1^. Moreover, they reported the assembly of an AHSa device architecture using AC-PPD-PQB and NiCoAl-LDH@CNT electrodes. The NiCoAl-LDH@CNT//AC-PPD-PBQ ASC device exhibited the maximum *C*_sp_ of about 158 and 144 F g^−1^ at 5 mV s^−1^ and 2 A g^−1^, respectively. The device was tested in a wide potential window of 1.8 V and delivered an energy density of 70.9 W h kg^−1^ at a power density of 709 W kg^−1^ together with cycling stability of 84.4% *C*_sp_ retention of its initial value after 5000 cycles. This work emphasized the fabrication of covalently grafted redox-active quinone in a microporous framework, exhibiting high performances. This can afford a new pathway to fabricate high-performance charge storage devices using these hybrid electrodes. Jia *et al.* reported the fabrication of a three-dimensional graphene electrode functionalized with the molecular mixture of hydroquinone and 2,5-(di-*p*-phenylenediamine)-1,4-benzoquinone (DBP).^82^ Among the pared electrodes, DFGN-1 displayed the *C*_sp_ of 667.3 F g^−1^ at 1 A g^−1^ with 89.2% retention at 50 A g^−1^.^82*a*^ The same electrode in its flexible device delivered the *C*_sp_ of 441 F g^−1^ at 0.5 A g^−1^ with 90.6% cycling stability after 10 000 GCD cycles at 10 A g^−1^. The relationship between the energy and power density was expressed using the Ragone plot. The estimated energy density of 9.29 W h kg^−1^ was obtained at a power density of 96.22 W kg^−1^. The FSSC device maintained a power density as high as 1.28 kW kg^−1^ at an energy density of 0.34 W h kg^−1^. The charge-storage capacity results of DFGN-1 are superior to that of vanadium oxide (V_2_O_5_)-based electrode materials, which exhibited the *C*_sp_ of ∼141.8 F g^−1^.^82*b*^ Very recently, our group demonstrated the fabrication of the GO-BAPh-BQ-BAPh-GO electrode material based on 2,5-bis((4-aminophenyl) amino) cyclohexa-2,5-diene-1,4-dione (2NH2-Ph-BQ) as pillars between graphene oxide (GO) sheets. The SSC device delivered a *C*_sp_ of 147.67 F g^−1^ at a current density of 0.5 A g^−1^ and ED of 36.18 W h kg^−1^ at a PD 1259.98 W kg^−1^.^82*c*^ This provides a novel way to construct composite electrodes based on quinone and its derivatives and their applications in charge storage devices. Herein, we conclude that researchers have established that quinone organic compounds can be utilized as sustainable pseudocapacitor electrode materials for high-performance charge-storage SC applications.

#### 4.1.2. Quinone-based polymers for supercapacitor applications

**Fig. 9:**
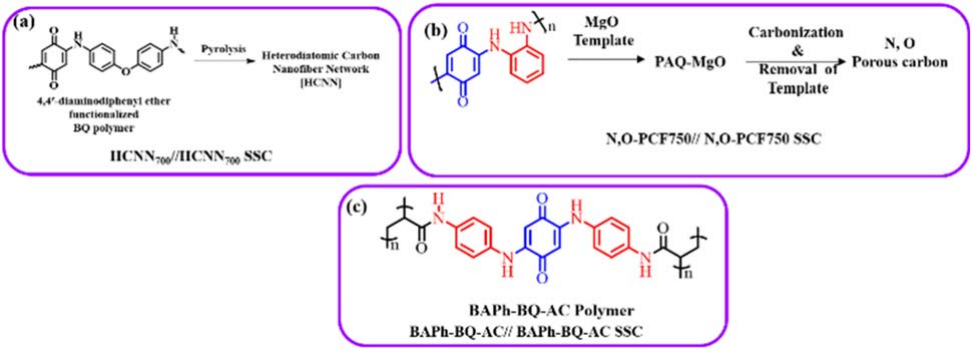
Benzoquinone (BQ)-based polymer structures and materials: (a) HCNN_700_, (b) N,O-PCF_750_ and (c) BAPh-BQ-AC.

Carbon materials with low cost and high surface area are environmentally friendly for use in energy storage applications.^83^ In this context, structural engineering is a crucial factor for modulating carbon nanostructures to enhance the active surface area and electrolyte ion migration in carbon electrodes for boosting the energy storage efficiency of SC devices.^84^ To achieve this type of active carbon electrode material, Liu and co-workers synthesized a polymer based on electron-withdrawing benzoquinone and electron-donating 4,4′-diaminodiphenyl ether *via* a coupling reaction (Fig. 9).^85^ The as-prepared quinone-based polymer upon pyrolysis was converted into interwoven heterodiatomic carbon nanofiber networks (HCNN) such as HCNN_600_, HCNN_700_, HCNN_800_ and HCNN_900_ (Fig. 9a). The HCNN_600_, HCNN_700_, HCNN_800_ and HCNN_900_ carbon fiber networks in aqueous 7 mol per kg LiCF_3_SO_3_ electrolyte in the operational potential voltage window of 0–2.2 V at a current density of 1 A g^−1^ a *C*_sp_ of 131, 249, 195 and 169 F g^−1^, respectively. The HCNN_700_ electrode delivered an energy density as high as 41.8 W h kg^−1^ at a power density of 450 W kg^−1^. The higher charge-storage performance of the HCNN_700_ electrode was examined by N_2_ adsorption–desorption isotherms. The type-I profile of the BET curve from the nitrogen adsorption–desorption isotherm of HCNNX indicates the presence of small and an abundant proportion of mesopores and micropores, respectively. The micropore areas contribute to the improvement in electric double-layer charge storage, whereas the micro-mesopore domain decreases the ion diffusion barrier, consequently enhancing the accessibility of the carbon framework internal pore surface. The surface area of HCNN_600_, HCNN_700_ and HCNN_800_ increased to 1751 m^2^ g^−1^, 2384 m^2^ g^−1^ and 2905 m^2^ g^−1^, respectively, whereas it decreased for HCNN_900_. A similar trend was found for the pore volume of HCNN. According to the BET analysis parameters, it was observed that HCNN_700_ displayed a higher surface area of 2384 m^2^ g^−1^ and pore volume of 1.27 cm^3^ g^−1^ (micropores: 0.92 cm^3^ g^−1^ and mesopores: 0.28 cm^3^ g^−1^), which could enhance the ion diffusion and accessibility of the active electrode surface, resulting in a higher electrochemical performance. The HCNN_700_-based SC exhibited 71.7% capacitance retention and 98.2% coulombic efficiency after 20 000 cycles at 10 A g^−1^. The high performance of the HCNN_700_ electrode could be attributed to its pore structure, heteroatom doping levels and geometry. This indicates that the as-prepared quinone-amine coupling route and pyrolysis of the carbon network structures result in the formation of efficient materials for SC applications. The HCNN-based Zn-ion SC delivered the *C*_sp_ of 220 mA h g^−1^ at 20 A g^−1^ and exhibited cycling stability of 85.3% after 20 000 cycles. Thus, this work offers a new polymer design for the production of carbon network electrodes with improved energy storage performances.

**Fig. 10:**
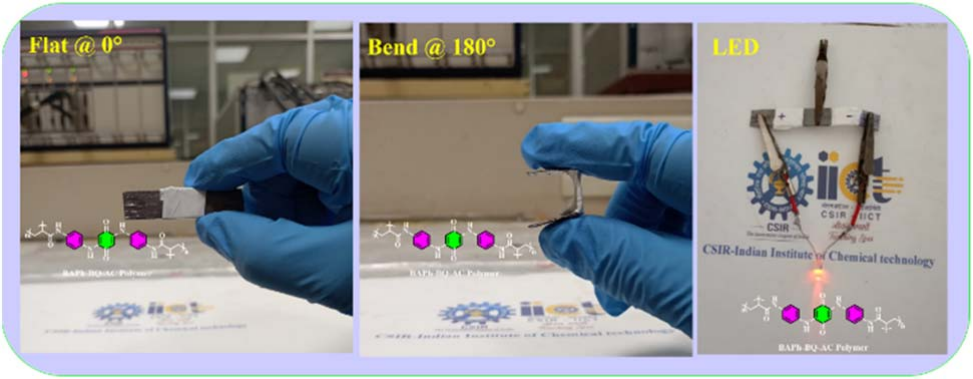
Photograph of the FSSC current collector collected at 0° and 180° bending angles and illumination of a light-emitting diode (LED) powered by the FSSC device. Reproduced from ref. 87 with permission from [the American Chemical Society], Copyright [2024].

Guo and co-workers developed a quinone-amine polymer (PAQ), which was further carbonized on the surface of nanosized MgO to yield carbon foam after the removal of MgO with acetic acid (Fig. 9b).^86^ The symmetric SC devices based on the as-prepared carbon foam named N,O-PCF_750_ in 1 M H_2_SO_4_ electrolyte yielded a *C*_sp_ of 321 F g^−1^ at 1 A g^−1^ with the energy density of 15.91 W h kg^−1^ at the power density of 0.4 kW kg^−1^.^86*a*^ The as-fabricated SSC device at 5 A g^−1^ displayed cycling life with 98% *C*_sp_ retention after 15 000 cycles. The excellent charge storage performance of the as-prepared N,O-PCF_750_-based SC could be ascribed to its micro–meso–macro pore structure and higher number of faradaic-active subunits present in the quinone-amine polymer. The porous properties of N,O-PCF750 was examined by means of BET analysis using nitrogen adsorption–desorption measurements. The BET analysis of N,O-PCF750 displayed an SSA of 1215 m^2^ g^−1^, in which the contribution of the microporous area is 304 m^2^ g^−1^. It has been well documented that an increase in surface area is useful to enhance the performance of supercapacitors.^86*b*^ The Barrett–Joyner–Halenda (BHJ) analysis of N,O-PCF750 showed the pore volume and pore size of 1.5 cm^3^ g^−1^ and 5.3 nm, respectively. The larger surface area of N,O-PCF750 provides the basis for the EDLC behaviour of the electrode and easy pathway for electrolyte ion diffusion.^86*c*^ Very recently, our group examined the charge storage characteristics of the polymer BAPh-BQ-AC (Fig. 9c) derived from 1,4-diaminobenzene (DAPh) and benzoquinone (BQ).^87^ The flexible device based on the BAPh-BQ-AC/graphite foil (GF) electrode in a poly(vinyl alcohol) (PVA)/H_2_SO_4_ gel electrolyte at bending angles of 0° and 180° (Fig. 10) exhibited *C*_sp_ of 102.39 and 99.59 mF cm^−2^ at a current density of 0.5 mA cm^−2^, respectively. It also delivered an energy density of 17.90 μW h cm^−2^ at a power density 1.76 mW cm^−2^ at 0.5 mA cm^−2^. The flexible SSC device showed an excellent electrochemical performance, which could be attributed to the presence of pseudocapacitive moieties in the BAPh-BQ-AC polymer. This material will be interesting to design and prepare wearable and electronic devices for real-world applications. The FSSC device was successfully utilized for the illumination of an LED light (Fig. 10).

#### 4.1.3. Quinone-based covalent organic framework (COF) for supercapacitor applications

**Fig. 11:**
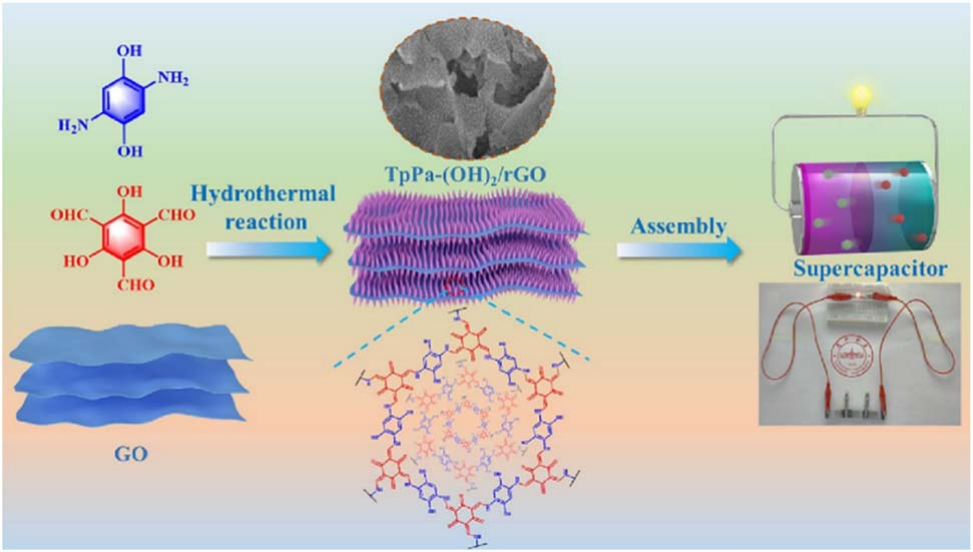
Schematic of the preparation of the (TpPa-(OH)_2_)/rGO material, its assembly in an SC device and three SCs applied to light an LED lamp. Reproduced from ref. 89b with permission from [Elsevier], Copyright [2024].

Covalent organic frameworks (COFs) are utilized in electrochemistry applications.^88^ However, it has been observed that pure COFs display limitations such as low electronic conductivity and restricted accessibility, restricting their practical EES applications. Thus, to overcome these limitations, COFs based on redox-active organic moieties and conducting carbon materials have been found to enhance the charge storage property. In this regard, graphene due to their synthetic scalability and higher electron conductivity acts as the most promising material for fabrication of SC electrodes.^89*a*^ In this context, Du and co-workers successfully synthesized a 2D COF material named (TpPa-(OH)_2_) nanowires, which was further anchored on reduced graphene oxide *via* the hydrothermal method to yield the (TpPa-(OH)_2_)/rGO electrode (Fig. 11).^89*b*^ (TpPa-(OH)_2_)/rGO displayed a *C*_sp_ of 371.1 F g^−1^ at a current density of 0.5 A g^−1^. Moreover, the (TpPa-(OH)_2_)/rGO-based SC device showed excellent cycling stability of 93% after 20 000 GCD cycles at 10 A g^−1^. The TpPa-(OH)_2_/rGO-3//TpPa-(OH)_2_/rGO-3 SSC cell configuration delivered a *C*_sp_ of 197.1 F g^−1^ at 0.2 A g^−1^ with a specific energy of 16.6 W h kg^−1^ at a power density of 158.7 W kg^−1^.^89*b*^ Three SC devices in series were successfully utilized to illuminate an LED lamp (Fig. 11). The higher electrochemical performance could be attributed to the pseudo-capacitive process shown by the redox-subunits present in the COF and the higher conducting rGO material.

**Fig. 12:**
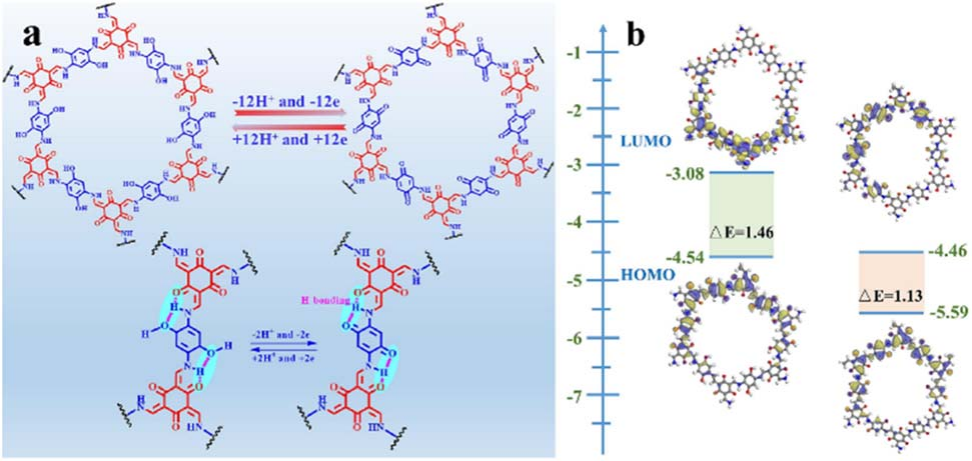
(a) Plausible reversible faradaic redox-activity of (TpPa-(OH)_2_) and (b) corresponding HOMO and LUMO energy levels of (TpPa-(OH)_2_) in the charge–discharge process. Reproduced from ref. 89b with permission from [Elsevier], Copyright [2024].

The possible reversible redox-activity of (TpPa-(OH)_2_)/rGO in acidic medium is demonstrated in Fig. 12. Two-electron oxidation/reduction processes take place at a single phenolic hydroxyl functional group, whereas reversible twelve-electron transfer takes place within a single (TpPa-(OH)_2_) COF ring system (Fig. 12a). The authors claimed that the C=O functional group of benzoquinone undergoes non-covalent hydrogen bonding with an adjacent amino group within the COF, inhibiting the decomposition of benzoquinone.^90^ Furthermore, Chandra *et al.* demonstrated the texture of a COF using BET analysis. TpPa-(OH)_2_ and TpBD-(OH)_2_ exhibited type-I adsorption isotherms. The BET analysis displayed the surface areas of 369 and 197 m^2^ g^−1^ together with the pore volumes of 0.417 and 0.241 cm^3^ g^−1^ for TpPa-(OH)_2_ and TpBD-(OH)_2_, respectively. The moderate surface area shown by TpPa-(OH)_2_ and TpBD-(OH)_2_ could be ascribed to the lack of sufficient long-range ordering in their 2D COF structures. The HOMO and LUMO of (TpPa-(OH)_2_) are presented in Fig. 12b. These frontier molecular orbitals are utilized to establish the energy levels of (TpPa-(OH)_2_)/rGO during the charging–discharging process. The calculated HOMO–LUMO energy gap of TpPa-(OH)_2_ was found to be 1.46 eV, which could be ascribed to the extended π-electron overlap in the highly conjugated COF system. The hydroquinone form of TpPa-(OH)_2_ was converted into quinone form during the charge–discharge process, and the LUMO energy level decreased to −4.46 eV, which resulted in a reduction in the energy gap to 1.13 eV (Fig. 12b). The reduction in the energy gap between the HOMO and LUMO energy levels suggests the faster redox reaction kinetics and excellent electronic conductivity characteristics of the TpPa-(OH)_2_/rGO electrode.^89*b*,91^

However, although COFs in combination with conducting carbon materials have shown an improvement in charge storage properties, due to their organic construction, these electrode frameworks lose their charge slowly, resulting in limited conductivity. Therefore, the importance of higher cycling life and cycling stability cannot be overstated. It is further interesting to note that 1,4-naphthoquinone (NQ) and its derivatives in the quinone family can be employed for SC applications.

Table 2 comparison of electrochemical properties of quinone-based small molecules, polymers and covalent organic frameworks (COFs). Quinones (AQs) have attracted interest for charge-storage applications for decades. AQs can be utilized as either the electrolyte or electrode materials in SC systems. As shown in Table 2, AQ as the supporting electrolyte with 1 M H_2_SO_4_ displayed the highest *C*_sp_ of 5017 F g^−1^ and energy density as high as 30.6 W h kg^−1^.^63^ In contrast, a small molecule AQ-based electrode in combination with rGO displayed the highest *C*_sp_ of 2646 F g^−1^ at 0.5 mA cm^−2^.^67^ The rGO-AHQDME-based SSC exhibited the highest energy density of 143 W h kg^−1^ at 1745 W kg^−1^.^73^ Among the numerous possibilities, polymeric electrode materials based on AQs are best suited for SC applications. In this case, the AQ-based HCNN_700_ polymer displayed the best performance with a *C*_sp_ of 249 F g^−1^ at 1 A g^−1^ and energy density as high as 41.8 W h kg^−1^ at a power density of 450 W kg^−1^ (Table 2).^85^ These results indicate that AQ in its smaller molecular entity and in its polymeric form are effective in providing a high specific capacitance, energy density and power density, suggesting their potential utilization in next-generation SC applications.

**Table 2:** Comparison of electrochemical properties of quinone-based small molecules, polymers and covalent organic frameworks (COFs)

Compound code	Electrolyte	Type of working electrode	Specific capacitance (*C*_sp_)	Energy density (ED)	Power density (PD)	Ref.
**Quinone small molecules/other materials**						
Carbon material	1 M H_2_SO_4_ + quinone/hydroquinone, (Q/HQ) supporting electrolyte	Three-electrode	Anode 5017 F g^−1^	30.6 W h kg^−1^	—	63
			Cathode 477 F g^−1^			
Activated carbon material	1 M H_2_SO_4_/(Q/HQ) supporting electrolyte	Two-electrode	901 F g^−1^ at 2.65 mA cm^−2^	31.3 W h kg^−1^	—	66
P-BQHQ	1 M H_2_SO_4_/AcOH (30%)	Three-electrode	2646 F g^−1^ at 0.5 mA cm^−2^	—	—	67
DMQ@rGO	1 M H_2_SO_4_	Three-electrode	650 F g^−1^ at 5 mV s^−1^	—	—	68
Slurry electrode	1 M H_2_SO_4_ and 0.38 M HQ	Two-electrode	513 F g^−1^ at 2 mV s^−1^	∼14 W h kg^−1^	103 W kg^−1^	69
TABQ-MWCNTs	1 M H_2_SO_4_	Two-electrode ASC	Specific capacity 57.3 F g^−1^ at 1 A g^−1^	15.6 W h kg^−1^	700 W kg^−1^	72
rGO-AHQDME	Acetonitrile (AN)/EMIMBF_4_ (1 : 1 mass ratio)	Two-electrode SSC	338 F g^−1^/100% at 1.0 A g^−1^	143 W h kg^−1^	1745 W kg^−1^	73
ABQA-GO/CP	1 M H_2_SO_4_	Two-electrode SSC	134 F g^−1^ at 0.5 A g^−1^	32.87 W h kg^−1^	1256 W kg^−1^	75
Templated carbon	1 M H_2_SO_4_ redox additive 2 mM DHBS-2	Two-electrode SSC	112 F g^−1^ at 1 A g^−1^	15.6 W h kg^−1^		76
	1 M H_2_SO_4_ redox additive 2 mM MHQ-2		88 F g^−1^ at 1 A g^−1^			
BQ-DNA/rGO	1 M H_2_SO_4_	Two-electrode	142.65 F g^−1^ at 0.5 A g^−1^	25.67 W h kg^−1^	1080 W kg^−1^	77
BQ-DP/GS	1 M H_2_SO_4_	Two-electrode	247 F g^−1^ at 9.5 A g^−1^	∼51 W h kg^−1^	>15 000 W kg^−1^	78
AC-PPD-PQB	6 M KOH	Two-electrode ASC	144 F g^−1^ at 2 A g^−1^	70.9 W h kg^−1^	709 W kg^−1^	81
DFGN-1	PVA-H_2_SO_4_ hydrogel	FSSC	441 F g^−1^ at 0.5 A g^−1^	9.29 W h kg^−1^	96.22 W kg^−1^	82a
GO-BAPh-BQ-BAPh-GO	1 M H_2_SO_4_	Two-electrode SSC	147.67 F g^−1^ at 0.5 A g^−1^	36.18 W h kg^−1^	1259.98 W kg^−1^	82c

**Quinone based polymers**						
HCNN_700_	LiCF_3_SO_3_	Two-electrode SSC	249 F g^−1^ at 1 A g^−1^	41.8 W h kg^−1^	450 W kg^−1^	85
N,O-PCF_750_	1 M H_2_SO_4_	Two-electrode SSC	321 F g^−1^ at 1 A g^−1^	15.91 W h kg^−1^	0.4 kW kg^−1^	86
BAPh-BQ-AC/graphite foil (GF)	PVA/H_2_SO_4_ gel electrolyte	FSSC	102.39 mF cm^−2^ (0°) and 99.59 mF cm^−2^ (180°) at 0.5 mA cm^−2^	17.90 μW h cm^−2^	1.76 mW cm^−2^	87

**Quinone based COFs**						
TpPa-(OH)_2_/rGO	1 M H_2_SO_4_	Two-electrode SSC	197.1 F g^−1^ at 0.2 A g^−1^	16.6 W h kg^−1^	158.7 W kg^−1^	89b
TpPa-(OH)_2_	1 M phosphate buffer	Two-electrode SSC	214 F g^−1^ at 0.2 A g^−1^	—	—	90

#### 4.1.4. Naphthoquinone-based supercapacitors

**Scheme 2:**
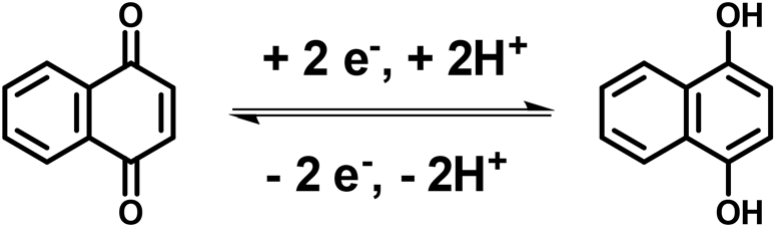
Reversible redox-reactions of naphthoquinone during an electrochemical process.

Naphthoquinones are the naturally occurring organic compounds obtained in carnivorous plants such as *Dionaea* and *Drosera* species.^92^ 1,4-Naphthoquinone (NQ) is synthetically derived from naphthalene.^93^ NQs with six carbon atoms and two oxygen atoms in their structure are capable of storing two protons and two electrons and undergoing reversible redox-reactions in aqueous solution (Scheme 2).^94^

**Fig. 13:**
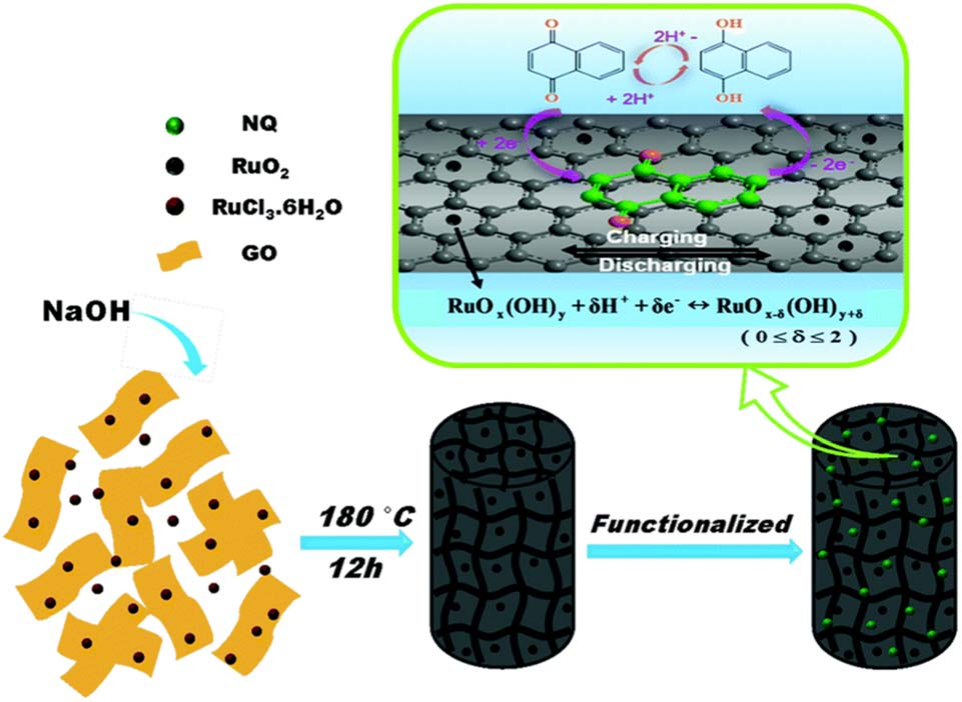
Schematic presentation of the synthesis of the NQ-RuO_2_/SGH composite electrode material. Reproduced from ref. 96 with permission of RSC.

Naphthoquinone (NQ) and its derivatives bearing two –C=O functional groups are important entities due to their reversible redox reactions (Scheme 2). These NQs are utilized to fabricate electrode materials for pseudocapacitor applications. Presser and co-workers reported the preparation of a quinone-decorated onion-like carbon electrode for PSC applications in 1 M H_2_SO_4_ as the electrolyte.^95^ The BET analysis of the onion-like carbon (OLC) was performed by recording their nitrogen gas sorption/desorption profiles examined at −196 °C. According to the BET analysis of OLC, it exhibited an SSA of 520 m^2^ g^−1^. These results demonstrate the presence of dense carbon nanoparticles in the carbon onion framework. The charge-storage process was investigated by means of cyclic voltammetry (CV) and galvanostatic charge–discharge (GCD) methods. The PSC device with naphthoquinone-functionalized onion-like carbon (NQ-OLC) compared to the only onion-like carbon (OLC)-based electrode showed an increase in *C*_sp_ from 30 F g^−1^ to 91 F g^−1^ and energy density (*E*_d_) from 0.5 W h kg^−1^ to 1.5 W h kg^−1^, respectively. The present authors demonstrated the pseudocapacitive performance of the SC device and the successful use of NQ to improve the energy density and cycling stability of the OLC electrodes. Zhang and co-workers reported the fabrication of a hybrid NQ-RuO_2_/SGH electrode material from naphthoquinone and RuO_2_ in combination with a graphene hydrogel (Fig. 13).^96^ They examined the electrochemical properties of the SGH, RuO_2_/SGH and NQ-RuO_2_/SGH electrode materials. NQ was anchored on the RuO_2_/SGH material *via* π–π stacking interactions. The BET analysis using the nitrogen adsorption/desorption isotherms of SGH, RuO_2_/SGH and NQ-RuO_2_/SGH was performed to estimate their SSA and pore size distribution (PSD). SHG exhibited a type-IV isotherm, suggesting the presence of mesopores and macropores in its structural framework. The BET SSA and average pore size of SGH were found to be 332.8 m^2^ g^−1^ and 5.4 nm, respectively. When RuO_2_ was anchored on the SHG surface, the macropores in the RuO_2_/SGH composite disappeared, whereas the mesopores were still exists. The BET analysis of the RuO_2_/SGH composite showed a slight decrease in its SSA and average pore size to 302.8 m^2^ g^−1^ and 3.5 nm, respectively. In contrast, the NQ-RuO_2_/SGH electrode material displayed a sharp decrease in SSA to 30.8 m^2^ g^−1^, whereas an increase in average pore size to 8.6 nm. The estimated *C*_sp_ for SGH, RuO_2_/SGH and NQ-RuO_2_/SGH according to their GCD curves at a current density of 1 A g^−1^ is 176.2, 371.4 and 450.8 F g^−1^, respectively. The higher *C*_sp_ of the NQ-RuO_2_/SGH composite electrode could be ascribed to (i) the 3D network of the graphene hydrogel, which can enhance the contact between the electrolyte and electrode, (ii) the faster transmission of H^+^ ions, (iii) the rapid charge transport due to the uniformly anchored Ru_2_O nanoparticles and NQ molecules on the graphene hydrogel surface and (iv) faster redox reactions displayed by NQ. Thus, the composite electrode showed EDLC and pseudocapacitive behavior, resulting in a higher *C*_sp_. Moreover, the authors demonstrated the fabrication of an ASC cell, which displayed a *C*_sp_ of 60.1 F g^−1^ at 1 A g^−1^ and energy density as high as 16.3 W h kg^−1^ at 0.7 kW kg^−1^ in aqueous 1 M H_2_SO_4_ electrolyte solution. This can be ascribed to the presence of mesopores and macropore textures in the structure of the electrode. It has been well documented that macropore electrode materials can provide the basis for shortening the diffusion length of electrolyte ions, whereas mesopores and micropores can enhance the charge storage capability, enhancing the energy density and power density of the electrode materials. Therefore, the as-fabricated ASC MNC//NQ-RuO_2_/SGH ASC possessed abundant mesopores and macropores in its positive electrode framework, whereas its negative electrode contained mesopores and micropores. Therefore, the MNC//NQ-RuO_2_/SGH ASC exhibited higher energy and power densities. Thus, the inorganic and organic hybrid composite NQ-RuO_2_/SGH electrode revealed the importance of the properties of organic and inorganic materials in SC applications.

**Fig. 14:**
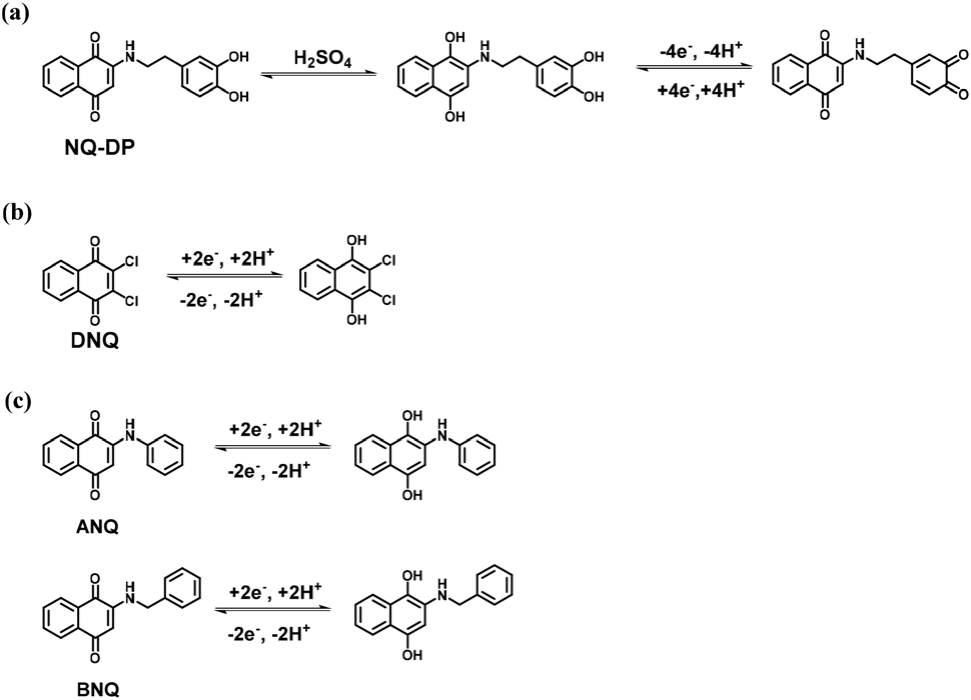
Proposed reversible redox-mechanism of (a) NQ-DP, (b) DNQ and (c) ANQ and BNQ.

In 2019, Miao and co-workers reported the preparation of the NQ-modified nitrogen-oxygen co-doped carbon nanotube (CNT) electrode material, denoted as NQ/N-O-CNT, and their SC applications.^97^ In the three-electrode SC configuration, they performed GCD measurements at 1 A g^−1^ in the applied potential window of −0.95 to 0 V in the presence of an aqueous 6 M KOH electrolyte solution. The CNT, O-CNT, N-O-CNT, NQ/N-O-CNT, O-NQ/O-CNT and O-NQ/N-O-CNT electrode material-based SCs displayed *C*_sp_ of 25.78 F g^−1^, 30.38 F g^−1^, 30.71 F g^−1^, 74.60 F g^−1^, 98.19 F g^−1^ and 143.68 F g^−1^, respectively. The *C*_sp_ of O-NQ/N-O-CNT was 4.5-times higher than that of the CNT electrode. Moreover, the O-NQ/N-O-CNT electrode exhibited 83.6% retention of its original *C*_sp_ value after 3000 cycles. The present results demonstrate the importance of NQ organic compounds in electrode materials for pseudocapacitor applications. In recent years, not only NQs have been utilized for the preparation of active-electrode materials for pseudocapacitor applications but also functionalized NQs have been systematically employed. In this case, Bhosale and co-workers demonstrated the synthesis and application of a new organic material, 2-((3,4-dihydroxyphenethyl)amino)naphthalene-1,4-dione (NQ-DP) (Fig. 14).^98^ The NQ-DP molecular structure was designed based on the redox chemistry of NQ and dopamine (DP). They fabricated the NQ-DP/CP electrode using NQ-DP and Toray carbon paper (CP). In the three-electrode SC and two-electrode SSC, at a current density of 0.5 A g^−1^, NQ-DP/CP displayed the *C*_sp_ of 160.8 F g^−1^ and 65.9 F g^−1^, respectively. The *C*_sp_ originated from the faradaic reversible redox reactions (Fig. 14a) of the organic electrode materials, indicating the pseudocapacitive behaviour of the device. Hou and co-workers utilized 2,3-dichloro-1,4-naphthoquinone (DNQ) as an active organic material for pseudocapacitor applications (Fig. 14b).^99^ They fabricated the DNQ@rGO composite electrode material *via* the non-covalent modification of reduced graphene oxide (rGO) with DNQ redox species. The optimized PSC device based on the DNQ@rGO electrode in 1 mol per L H_2_SO_4_ electrolyte exhibited the *C*_sp_ of 361.2 F g^−1^ at 5 mV s^−1^ and displayed cycling stability of 87.5% at a sweep rate of 100 mV s^−1^. The charge-storage properties arose from the reversible redox reactions of the electrode material (Fig. 14b). Moreover, the asymmetric supercapacitor (ASC) device of HLGH//DNQ@rGO in 1 M H_2_SO_4_ electrolyte in the applied voltage window 0 to 1.6 V showed the *C*_sp_ of 60.6 F g^−1^ at 5 mV s^−1^. The ASC device achieved an energy density of 16.6 W h kg^−1^ at a power density of 0.7 W kg^−1^. The BET analysis based on the N_2_ adsorption–desorption isotherms of the bare rGO displayed an SSA of 456.6 m^2^ g^−1^, which is higher than that of DNQ@rGO (219.14 m^2^ g^−1^). The sharp decrease in the SSA of the DNQ@rGO composite electrode material could be due to the covering of the some micropores in rGO by the DNQ organic molecules. The hysteresis loop and the relatively low pressure than 0.1 indicate the presence of mesopores and micropores in the electrode material. In addition, the authors confirmed the presence of macropores in the material with the help of the steep vertical tails that appeared at a higher relative pressure. According to the size distribution curve, the DNQ/rGO-1 composite displayed less micropores than that of bare rGO. The presence of macropores with a pore size of 10–100 nm favour an enhancement in the specific capacitance of the DNQ@rGO electrode materials. In addition, the macropores in the DNQ@rGO material act as a electrolyte ion-buffering reservoir. This will help minimize the distance between the graphene thin layers for ion diffusion, which accelerates the transportation of ions. Therefore, the as-fabricated DNQ@rGO composite electrode material with a hierarchical pore structure exhibited an excellent charge-storage performance. The excellent electrochemical performance of the organic NQ molecule will lead to greater research in this field to construct next-generation SCs. Very recently, Yoo and co-workers reported the synthesis and applications of 2-anilino-1,4-naphthoquinone (ANQ) and 2-benzylamino-1,4-naphthoquinone (BNQ) as an active organic material for charge-storage applications (Fig. 14c).^100^ They fabricated ANQ-AC and BNQ-AC electrodes using ANQ and BNQ in combination with activated carbon (AC) materials, respectively. The various compositions of ANQ and BNQ with AC were prepared. In 1 M H_2_SO_4_, the asymmetric SC device at a current density of 1 A g^−1^ exhibited the estimated *C*_sp_ of 76, 134 and 174 F g^−1^ for AC, AC : ANQ (3 : 1) and AC : BNQ (3 : 1), respectively. The BET analysis using the N_2_ adsorption–desorption isotherms of the AC-ANQ (3 : 1) and BC-ANQ (3 : 1) composite electrodes displayed their larger SSA and pore volume than that of AC. The authors observed that as the ANQ and BNQ content increased in the AC-ANQ and BC-ANQ composites, their SSA and pore volume decreased. This can be ascribed to the filling of the activated carbon framework with ANQ and BNQ molecules. This can lead to a reduction in SSA and pore volume, as confirmed by the BET analysis. The AC : ANQ and AC : BNQ composite electrode materials with a wt. ratio of 3 : 1 displayed superior charge-storage properties. The PSCs based on AC : ANQ (3 : 1) and AC : BNQ (3 : 1) exhibited good cycling stability of about 79.7% and 77.1% after 10 000 GCD cycles at 5 A g^−1^, respectively. The highest *C*_sp_ and good cycling stability of AC : ANQ and AC : BNQ compared to the AC electrode can be ascribed to the extra aromatic ring system present in the ANQ and BNQ molecular structures and the pseudocapacitive behaviour of the NQ derivatives (Fig. 14c). The aromatic ring system present in ANQ and BNQ allows extra π–π stacking interactions between them with AC. These findings are important for the design and development of new electrode materials based on the manipulation of the NQ molecular structure for enhancing the pseudocapacitor electrode performance. These materials have great potential in next-generation SC applications.

#### 4.1.5. Naphthoquinone COF supercapacitors

**Fig. 15:**
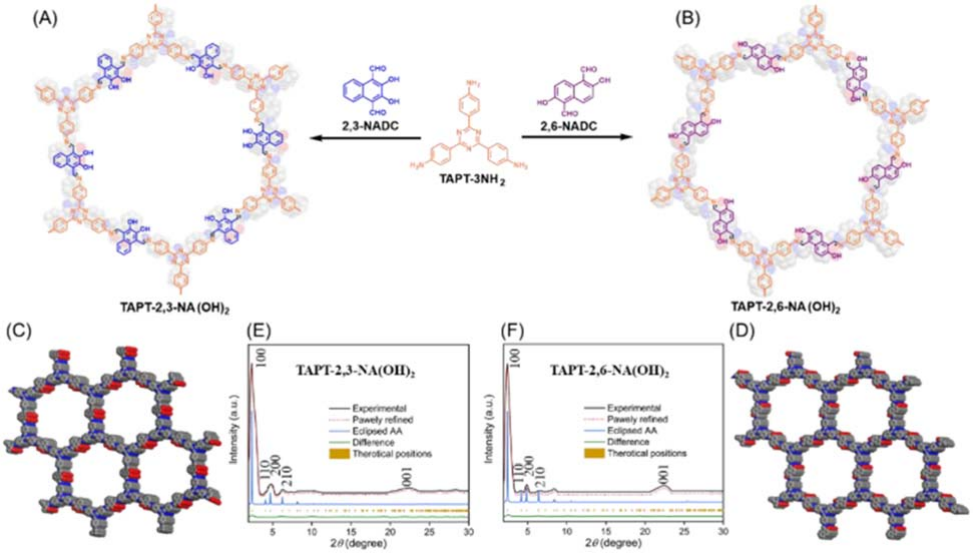
(A and B) Structures of TAPT-2,3-NA(OH)_2_ and TAPT-2,6-NA(OH)_2_ COFs. Top-view crystal structures of (C) TAPT-2,3-NA(OH)_2_ and (D) TAPT-2,6-NA(OH)_2_ COFs. Experimental and simulated PXRD patterns of (E) TAPT-2,3-NA(OH)_2_ and (F) TAPT-2,6-NA(OH)_2_ COFs. Reproduced from ref. 103 with permission from [MDPI], Copyright [2022].

Owing to the specific electrochemical properties of COFs, they have attracted attention from researcher over non-crystalline polymers. The redox-active subunits in the framework can be manipulated and their pore size controlled.^101^ In recent years, COF materials have been used as electrodes for real-world applications.^102^ In this regard, El-Mahdy and co-workers prepared two novel hydroxyl functionalized covalent organic frameworks, denoted as TAPT-2,3-NA(OH)_2_ and TAPT-2,6-NA(OH)_2_ COFs, starting from 1,3,5-tris-(4-aminophenyl)triazine (TAPT-3NH_2_) with 2,3-dihydroxynaphthalene-1,4-dicarbaldehyde (2,3-NADC) and 2,6-dihydroxynaphthalene-1,5-dicarbaldehyde (2,6-NADC), respectively, *via* a Schiff-base [3 + 2] polycondensation reaction (Fig. 15).^103^ The BET analysis using N_2_ sorption analysis at 77 K of the TAPT-2,3-NA(OH)_2_ and TAPT-2,6-NA(OH)_2_ COFs displayed type I isotherms. The small hysteresis loop displayed by the TAPT-2,3-NA(OH)_2_ and TAPT-2,6-NA(OH)_2_ COFs indicates their microporous nature as electrode materials. The BET SSA of the as-prepared TAPT-2,3-NA(OH)_2_ and TAPT-2,6-NA(OH)_2_ COFs was determined to be 429 and 1089 m^2^ g^−1^, with pore volumes of 0.17 and 0.22 cm^3^ g^−1^, respectively. The COFs displayed electrochemical characteristics due to the presence of redox-active hydroxyl subunits in TAPT-2,3-NA(OH)_2_ (Fig. 15A) and TAPT-2,6-NA(OH)_2_ (Fig. 15B). Fig. 15C and D display the top view of the crystalline structure of TAPT-2,3-NA(OH)_2_ and TAPT-2,6-NA(OH)_2_, respectively. The simulated and experimental powder X-ray diffraction patterns of TAPT-2,3-NA(OH)_2_ (Fig. 15E) and TAPT-2,6-NA(OH)_2_ (Fig. 15F) exhibit their long-ordered architectures with a triclinic network. The GCD measurements of the TAPT-2,3-NA(OH)_2_ and TAPT-2,6-NA(OH)_2_ COFs exhibited the *C*_sp_ of 271 F g^−1^ and 190 F g^−1^, respectively, at a current density of 0.5 A g^−1^. At 10 A g^−1^, the cycling stability of the TAPT-2,3-NA(OH)_2_ and TAPT-2,6-NA(OH)_2_ COFs was 79.1% and 74.5%, respectively, of their initial *C*_sp_ values after 5000 cycles. The energy density of the TAPT-2,3-NA(OH)_2_ and TAPT-2,6-NA(OH)_2_ COFs was 45.43 W h kg^−1^ and 31.11 W h kg^−1^, respectively, implying their use in industrial applications.

Thus, the molecular naphthoquinone moiety and its derivatives, together with the fabricated COF materials have been explored as electrodes in SC applications. We believe that these electrode materials will be useful in energy storage technologies and can be useful for a variety of wearable electronic applications.

Similar to AQ, the charge storage properties of naphthoquinone (NQ)-based small molecules and covalent organic frameworks (COFs) are summarized in Table 3. NQ-based small molecules display excellent electrochemical properties. Among the reported NQ electrode materials, NQ-RuO_2_/SGH in the three-electrode SC displayed the highest *C*_sp_ of about 450.8 F g^−1^ at 1 A g^−1^,^96^ whereas the highest energy density of 16.6 W h kg^−1^ at a power density of 0.7 W kg^−1^ was achieved by DNQ@rGO^99^ in a two-electrode ASC system. NQ embedded in the TAPT-2,3-NA(OH)_2_ COF displayed the highest *C*_sp_ of 190 F g^−1^ at 0.5 A g^−1^ and energy density as high as 45.43 W h kg^−1^.^103^ The excellent performance of small molecule-based electrodes and COFs makes NQs an interesting subject for further research. To ensure the competitiveness of NQs with conventional inorganic pseudocapacitor materials, it is important to design composite electrode materials.

**Table 3:** Comparison of the electrochemical properties of naphthoquinone-based small molecules and covalent organic frameworks (COFs)

Compound code	Electrolyte	Type of working electrode	Specific capacitance (*C*_sp_)	Energy density (ED)	Power density (PD)	Ref.
**Naphthoquinone-based small molecules**						
NQ-OLC	1 M H_2_SO_4_	Three electrode	91 F g^−1^	1.5 W h kg^−1^	—	95
NQ-RuO_2_/SGH	1 M H_2_SO_4_	Three electrode	450.8 F g^−1^ at 1 A g^−1^	—	—	96
		Two electrode ASC	60.1 F g^−1^ at 1 A g^−1^	16.3 W h kg^−1^	0.7 kW kg^−1^	
O-NQ/N-O-CNT	6 M KOH	Three electrode	143.68 F g^−1^ at 1 A g^−1^	—	—	97
NQ-DP/CP	1 M H_2_SO_4_	Two-electrode solid state SSC	43.4 F g^−1^ at 0.5 A g^−1^	6.0 W h kg^−1^	0.6 kW kg^−1^	98
		Two-electrode solid state ASC	65.9 F g^−1^ at 0.5 A g^−1^	9.0 W h kg^−1^	1.0 kW kg^−1^	
DNQ@rGO	1 M H_2_SO_4_	Two-electrode ASC	60.6 F g^−1^ at 5 mV s^−1^	16.6 W h kg^−1^	0.7 W kg^−1^	99
ANQ-AC	1 M H_2_SO_4_	Two-electrode ASC	134 F g^−1^ at 1 A g^−1^	—	—	100
BNQ-AC			174 F g^−1^ at 1 A g^−1^	—	—	

**Naphthoquinone-based COFs**						
TAPT-2,3-NA(OH)_2_	1 M KOH	Three-electrode	271 F g^−1^ at 0.5 A g^−1^	45.43 W h kg^−1^	—	103
TAPT-2,6-NA(OH)_2_ COFs			190 F g^−1^ at 0.5 A g^−1^	31.11 W h kg^−1^	—	

#### 4.1.6. Anthraquinone-based supercapacitors

**Scheme 3:**
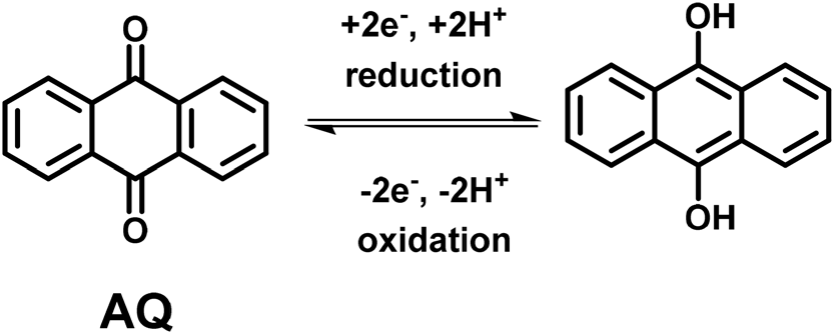
Reversible redox-reactions of anthraquinone (AQ) during electrochemical processes.

Anthraquinone (AQ) is an important pigment utilized in the dye and textile industries.^104^ AQs are abundant in nature.^105^ Owing to the fast reversible redox reactions of AQs with two electrons and two protons (Scheme 3), they are utilized to replace metal-based electrodes in energy storage applications.^106^

**Fig. 16:**
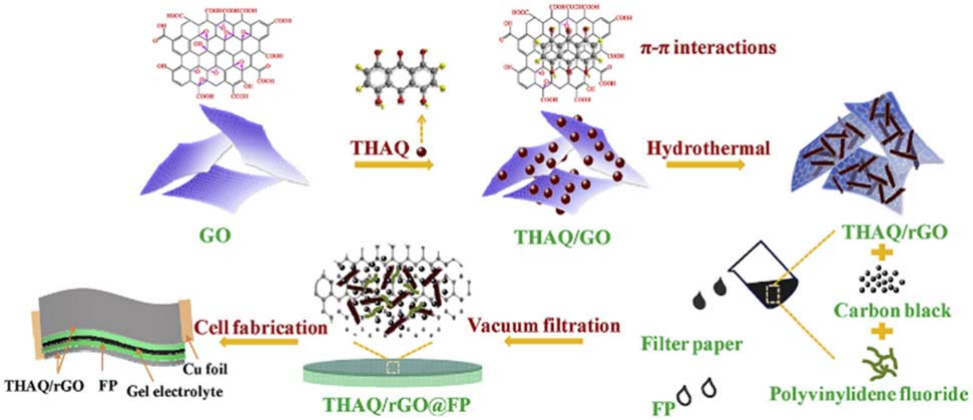
Schematic presentation of the method for the fabrication of the THAQ/rGO and flexible THAQ/rGO@FP electrodes. Reproduced from ref. 110 with permission from [Elsevier], Copyright [2018].

Theoretical calculations of AQs showed its energy storage capacity of 257 mA h g^−1^ in battery applications,^107^ making it an attractive and promising candidate for the fabrication of organic electrodes for next-generation SCs.^108^ In this case, in 2014, Wang and co-workers^108^ demonstrated the decoration of hierarchical porous carbon nanotubes (HPCNTs) using the AQ molecule. The as-fabricated AQ-HPCNT electrode in SC applications showed an excellent performance with *C*_sp_ of 710 F g^−1^ at 1 A g^−1^, which is higher than that of the unaltered HPCNTs (304 F g^−1^). It was also observed that at a higher current density, *e.g.* 20 A g^−1^, the *C*_sp_ retention was as high as 419 F g^−1^, implying the excellent rate capability of the device architecture. The percentage of AQ content plays a crucial role in the capacitance retention. This could be explained by means of the NET analysis of the electrode materials. The BET analysis using N_2_ adsorption–desorption isotherms of HPCNTs displayed a type IV hysteresis loop, suggesting the mesoporous structure of the material. The BJH method was utilized to demonstrate the pore size distribution of HPCNTs, which showed prominent peaks for micropores at 1.9 nm and mesopores at 3.7 nm. The SSA area derived from the BET analysis was found to be 2080 m^2^ g^−1^ with a pore volume of 1.23 cm^3^ g^−1^. Furthermore, the AQ-anchored HPCNTs with a 5 : 5 proportion displayed the characteristic type V isotherm. It is notable that upon the adoption of AQ molecules in the micropores and mesopores of HPCNTs, the as-fabricated AQ-HPCNT composite electrode material exhibited a decrease in SSA by up to 50 m^2^ g^−1^. This could result in the disappearance of most of the micropores and small mesopores. AQ-HPCNTs 5 : 5 displayed the total pore volume of 0.13 cm^3^ g^−1^, which is approximately 10-times smaller than of HPCNTs (1.23 cm^3^ g^−1^). An increase in AQ content in the AQ-HPCNT material resulted in a decrease in capacitance retention. The AQ-HPCNT electrode underwent a reversible reaction to yield charge storage properties. Its excellent performance could be attributed to the π–π stacking interaction between the AQ organic molecular scaffold and HPCNTs, which led to a strong positive synergistic effect between them. Further, to explore molecular core-modified AQs for SC applications, Lei and co-workers fabricated AQ, 1-AAQ-CC2 and 2-AAQ-CC2 electrodes using anthraquinone (AQ), 1-amino anthraquinone (1-AAQ) and 2-aminoanthraquinone (2-AAQ) by modifying the carbon materials *via* the absorption method.^109^ They found that the 1-AAQ-CC2 composite electrode in the SC device displayed an excellent pseudocapacitive performance with the *C*_sp_ of 328 F g^−1^ at 0.5 A g^−1^ and excellent cycling stability after 5000 cycles at 3 A g^−1^ with 95% retention of its initial *C*_sp_ value. It is noticeable that in the applied voltage window of 0 to 1.8 V, the SSC device with 0.5 mol per L Na_2_SO_4_ aqueous electrolyte showed an energy density as high as 14.8 W h kg^−1^ at a power density of 240 W kg^−1^.^109*a*^ The BET analysis based on the N_2_ adsorption/desorption profile displayed a decrease in the SSA of 1-AAQ-CC2 to 1214.1 m^2^ g^−1^ compared with that of CC2 of 1573.5 m^2^ g^−1^. The decrease in the SSA of 1-AAQ-CC2 could be ascribed to the adsorption of 1-AAQ in the micropores and mesopores of CC2. Accordingly, the author concluded that the modification of the surface of CC2 with 1AAQ resulted in the disappearance of most of the micropores and mesopores. This resulted in the total pore volume of 0.88 cm^3^ g^−1^ for 1-AAQ-CC2. Thus, the total pore volume of AAQ-CC2 was smaller than that of CC2 (1.13 cm^3^ g^−1^). Owing to the presence of micro/meso/macroscale pores in the composite electrode materials, they are ideal materials for SC applications. The actual charge storage occurs in the micropores and small mesoporous, whereas the macropores facilitate faster electrolyte ion transportation.^109*b*^ These charge storage results indicate the importance of organic–inorganic composite electrode materials for SC applications. The influence of the multi-substituent functional group at the AQ core on the pseudocapacitive properties was investigated by Li and co-workers in 2018.^110^ They demonstrated that 1,4,5,8-tetrahydroxy-functionalized anthraquinone (THAQ) with rich –C=O functional groups was an active redox-active organic electrode material for SCs. Initially, they prepared the active electrode material by anchoring THAQ on reduced graphene oxide (rGO) sheets *via* π–π stacking interactions. Further, using the vacuum-filtration method, a flexible electrode material was prepared using THAQ/rGO on filter paper (Fig. 16).^110^ The as-fabricated flexible electrode displayed high mechanical strength due to the filter paper (FP) and high electronic conductivity due to the rGO sheets. The rGO surface acted as an electronic transport medium to enhance the pseudocapacitive properties of THAQ. At 1 A g^−1^, the THAQ/rGO electrode exhibited a *C*_sp_ of about 259 F g^−1^ with 97.9% retention of its initial value after 10 000 cycles at a current density of 20 A g^−1^. Notably, the flexible device of THAQ/rGO@FP showed an areal capacitance as high as 122.7 mF cm^−2^ at a current density of 0.1 mA cm^−2^. The THAQ/rGO electrode provides a good solution to enhance the electronic conductivity as well as capacitive performance with robust mechanical properties of composite electrodes, which is an issue for AQ-based organic electrode materials in PSC applications.

**Fig. 17:**
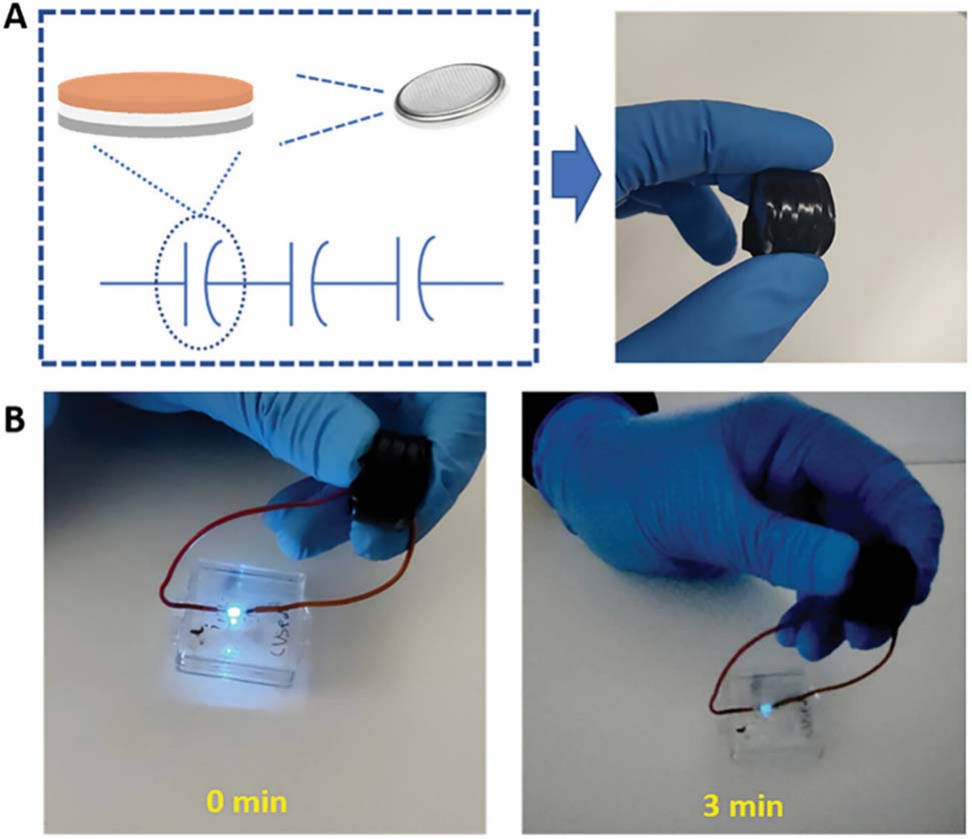
Bis-APDA/PANI-PPy/rGO ASC device in series: (A) diagram displaying three circularly shaped devices connected in series; (B) illumination of an LED lamp (30 mA forward current) after 0 and 3 min upon disconnecting the device. Reproduced from ref. 113 with permission from [John Wiley and Sons], Copyright [2023].

The preparation of inexpensive electrode materials for the development of cost-effective supercapacitor technology is a challenging task for researchers around the world. In this regard, naturally obtained cotton fabrics composed of cellulose fibers have emerged as low-cost materials for PSC applications. Jin and co-workers explored a carbonized cotton material as a substrate for the fabrication of electrodes.^111^ Initially, they decorated graphene sheets using 1,5-diaminoanthraquinone (DAQ) as an anchoring group *via* π–π stacking interactions. The obtained material was subjected to hydrothermal processing to achieve robust and flexible DAQ-CGH hydrogel electrode materials. The rGO material significantly acted as an electronic conducting medium and the carbonized cotton fiber substrate provided high mechanical strength to the electrode. The SC device displayed the *C*_sp_ of 490.2 F g^−1^ at 1 A g^−1^ with a specific capacitance retention of 89.93% after 10 000 cycles at 5 A g^−1^. Moreover, the device showed excellent mechanical flexibility with the *C*_sp_ retention of 92.15% of its initial value after 1000 bending cycles. At a current density of 0.5 A g^−1^, the highest energy density of 36.18 W h kg^−1^ was achieved at 125.02 W kg^−1^. In contrast, at 20 A g^−1^, the electrode maintained an energy density of 25.21 W h kg^−1^ at a power density of 2145.53 W kg^−1^ using the as-fabricated DAQ-2-CHG device architecture, indicating its potential for commercial use. In recent years, with the growing attention on green nanotechnology, the development of greener protocols for the synthesis of redox-active anthraquinone derivative-doped polyaniline (PANI) electrode material is required. In this case, Choi and co-workers reported a green route for the synthesis of high-surface area nanostructured materials with enhanced electrochemical performances using 9,10-anthraquinone-2-sulfonic acid sodium salt (AQSA) and PANI, denoted as PANI_AQSA.^112^ The AQSA molecular scaffold played a key role in regulating the PANI chain growth and morphology of the nanostructures. The as-fabricated PANI_AQSA nanotubular structures showed pseudocapacitive behaviour due to their faradaic reversible redox reactions. PANI_AQSA (1.5 concentration ratio of AQSA) in a three-electrode configuration in 1 M H_2_SO_4_ at 1 A g^−1^ exhibited the enhanced *C*_sp_ of 440 F g^−1^. In contrast, the PANI_AQSA//PVA_H_2_SO_4_//PANI_AQSA SSC device at 1 A g^−1^ showed an enhanced *C*_sp_ of 391 F g^−1^ with 93% retention of its *C*_sp_ initial value after 10 000 cycles. The higher performance of the PANI_AQSA electrode is attributed to the redox behaviour of AQSA and synergistic effect of PANI. It is also notable that the incorporation of AQSA in PANI widened the applied potential voltage window and cycling stability of the devices. To overcome the limitations and challenges posed by organic electrode materials and achieve practical breakthroughs using organic pseudocapacitor technologies, researchers are developing newer materials. In this regard, Jelinek and co-workers synthesized the polydiacetylene-anthraquinone (bis-APDA) monomer using PDAs and AQ subunits.^113^ The complementary diacetylenic monomer upon polymerization to polydiacetylene exhibited chromatic characteristics. Due to its photopolymerization network,^114^ the conjugated polymeric network bearing AQs^115^ enhanced the electronic conductivity of the electrode materials, which in turn enhanced the charge storage characteristics of the SC devices. They systematically demonstrated the synthesis and electrochemical properties of bis-APDA.^113^ The bis-APDA/PANI-based SC device configuration displayed a *C*_sp_ as high as ≈720 F g^−1^ at 1 A g^−1^.^113^ The bis-APDA/PANI cathode in combination with PPy/rGO as the anode in an ASC cell in the applied potential window in nonaqueous ionic liquid electrolyte (EMIM^+^HSO_4_^−^) in DMF exhibited a specific capacitance of 67 F g^−1^ at 1 A g^−1^ and an energy density as high as 36.2 W h kg^−1^ at a power density of 995 W kg^−1^. Herein, the synergistic effect of the redox and electronic conducting properties of AQ and PDA, respectively, enhanced the charge-storage, energy density and cycling stability characteristics of the SC devices. Further, in an ionic liquid electrolyte, the bis-APDA/PANI-PPy/rGO device was utilized for illuminating a blue light-emitting diode (LED) (30 mA forward current) with a 3-minute retention time.^113^ Thus, the as-fabricated electrode-based ASC cell showed an excellent energy density (Fig. 17). The present work demonstrated a new organic electrode design and its application in ASC devices with a higher energy density, which can be useful for the construction of a new generation of SC devices.

**Fig. 18:**
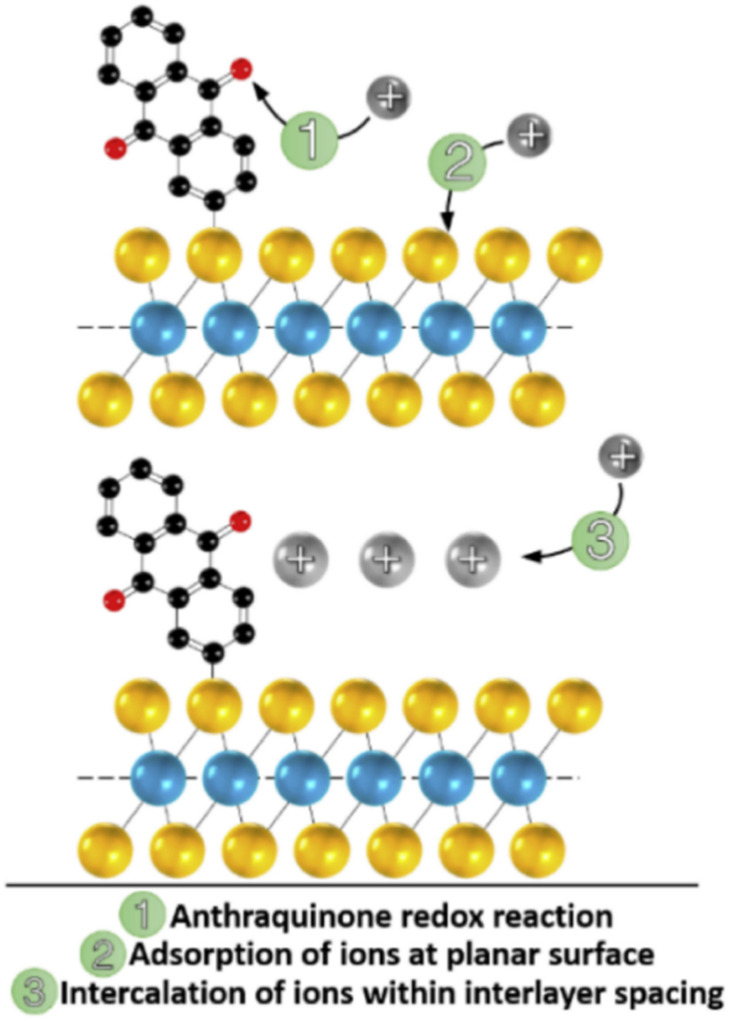
Proposed charge storage mechanism of the AQ-MoS_2_ electrode in aq. 1 M H_2_SO_4_. Reproduced from ref. 119 with permission from [Elsevier], Copyright [2024].

Although organic electrode materials are inexpensive, easy to synthesize and can their redox properties can be manipulated, the enhancement of the energy density of SC devices is a challenging task.^116^ Thus, to achieve a higher energy density in the SC cell configuration, the electrode materials should cover a wider voltage window profile. Bhosale and co-workers synthesized 2,3-bis(4-(3,6-di-*tert*-butyl-9*H*-carbazol-9-yl)phenyl)naphtho[2,3-*f*]quinoxaline-7,12-dione (DTCz-Pyz-AQ) molecular scaffolds based on donor and acceptor subunits, which were further utilized as active organic electrode materials for SC applications.^117^ They demonstrated that the as-fabricated DTCz-Pyz-AQ/GF electrode worked well in the potential voltage window of −0.4 to +0.4 V. The three-electrode SC configuration at 0.5 A g^−1^ yielded an impressive *C*_sp_ of 304.37 F g^−1^. In the DTCz-Pyz-AQ/GF//DTCz-Pyz-AQ/GF SSC device, the electrode in the applied potential window of 0 to 1.2 V exhibited a *C*_sp_ of 106.30 F g^−1^ at 0.5 A g^−1^ and displayed the 93% *C*_sp_ retention after 6000 GCD cycles at 2 A g^−1^. It is notable that this SSC device showed an excellent energy density of 15.94 W h kg^−1^ at the power density of 899.71 W kg^−1^. Thus, the present donor–acceptor electrode materials pave the new way to design newer organic molecular architectures for SC applications with a wider potential window, which in turn can be helpful to enhance the energy density of devices, and ultimately their electrochemical performance. To fulfill the increasing demand for high-performance SCs, researchers have devoted their efforts to searching for advanced electrode materials. For the development of high-performance electrodes capable of operating at high char-discharge rates, molybdenum disulfide (MoS_2_) has emerged as an attractive two-dimensional transition metal dichalcogenide (2D-TMDs) material.^118^ An enhancement in its specific capacitance can be achieved *via* the functionalization of the MoS_2_ material with organic redox-active moieties.^118^ Then the covalent functionalization of nanostructured MoS_2_ with redox-active anthraquinone (AQ) was achieved *via* diazonium chemistry.^119^ To examine the process of MoS_2_ functionalization with the AQ organic material, BET analysis was performed using N_2_ sorption measurements. The AQ-MoS_2_ electrode material exhibited a type II isotherm, which is characteristic of mesoporous samples.^119*b*^ The SSA of AQ-MoS_2_ increased upon the modification of MoS_2_ from 39 to 64 m^2^ g^−1^, suggesting the presence of AQ molecules within the interlayer spacing and on the surface of MoS_2_. The pore size distribution of the AQ-MoS_2_ electrode material remained comparable. The shift in onset of microporous range in the structure from 0.4 to 0.8 nm implies that the microporosity of MoS_2_ was blocked by the AQ organic molecules. The SC device based on the AQ-MoS_2_ electrode displayed a faradaic reversible redox-reaction response in 1 M H_2_SO_4_ electrolyte, indicating its pseudocapacitive behaviour. At 0.2 A g^−1^, the *C*_sp_ was enhanced from 191 F g^−1^ to 263 F g^−1^ after grafting AQ on the MoS_2_ surface.^119^ In the hybrid cell configuration (−)AQ-MoS_2_‖BP2000(+), in the applied potential window of −0.2 to 1.2 V, the device delivered a specific cell capacitance of 49 F g^−1^ at 0.1 A g^−1^. The proposed charge storage mechanism is displayed in Fig. 18. The blocking of the interlayer spacing of the MoS_2_ surface by AQ molecular entities resulted in an insignificant disruption in the ion transportation process during intercalation in aqueous media (Fig. 18), which enhanced the specific capacitance of the SC device.

**Fig. 19:**
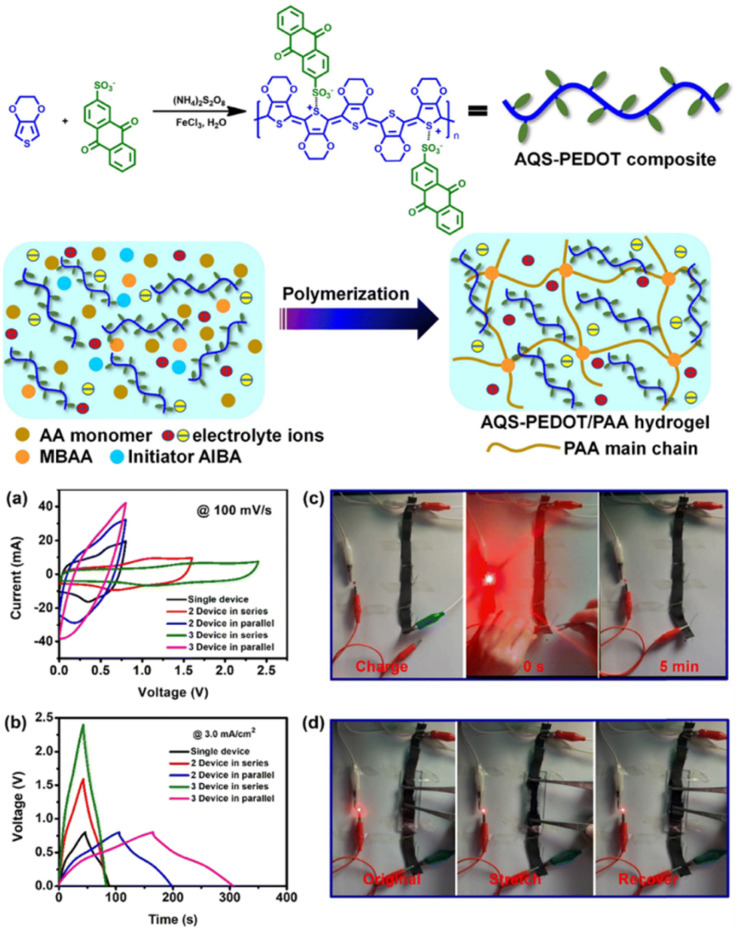
Preparation of the AQS-PEDOT composite and AQS-PEDOT/PAA hydrogel as active electrode materials; (a) CV profiles and (b) GCD profiles of the SCs based on PEDOT/PAA hydrogel electrode in series and parallel. Photograph of (c) red LED powered using the tandem SC group and (d) stretching of the SC cell configuration during the process of turning the LED. Reproduced from ref. 123 with permission from RSC.

To enhance the electrochemical properties of organic electrode materials, complementary electroactive subunits can be incorporated on the backbone of the AQ chemical structure. This can help increase the π-conjugation of the AQ subunit. Jagdale *et al.* demonstrated the synthesis and charge-storage properties of 2,6-bis((*E*)-(4-hydroxyphenyl)diazenyl) anthracene-9,10-dione (AZOAQ).^120^ The as-fabricated AZOAQ/GF composite electrode was utilized in three- and two-electrode SC configurations. The AZOAQ/GF//AZOAQ/GF SSC device delivered a *C*_sp_ of 159.12 F g^−1^ at 0.5 A g^−1^ with an excellent energy density of 28.64 W h kg^−1^ at a power density of 1080.02 W kg^−1^. Moreover, the device displayed 93.22% *C*_sp_ retention after 10 000 GCD cycles. The reported inexpensive organic material-based SSC device will be an attractive alternative to the existing inorganic metal oxide-based SSC devices to fulfill the demand of society in the coming years. EDLC electrode materials based on carbon exhibit a high electrochemical rate performance.^121^ Moreover, these carbon-based electrodes display good temperature tolerance. In contrast, at lower temperatures, pseudocapacitive carbon-based electrodes have not been thoroughly studied. To examine carbon electrodes for SC applications, Lai and co-workers investigated in detail the fabrication of pseudocapacitive materials based on redox-active molecular deposition at the inner pores of carbon electrodes.^122^ They deposited anthraquinone-2-sulfonic acid (AQS) on the inner pore surface of hierarchical porous carbon nanospheres (H-PC) to synthesize the H-PC@AQS electrode. To examine the pore structure and pore size of H-PC, 1D-PC, and B-PC, N_2_ adsorption–desorption measurements were carried out at low temperature. The pore size distribution was evaluated by means of the BHJ model. The vertical upward shape of the adsorption and desorption isotherms was observed, suggesting the higher ratio of micropores. The adsorption curve displayed a type-IV hysteresis loop, indicating the presence of micropores and mesopores in the materials. The calculated average pore diameters were found to be 2.9 nm, 2.8 nm, and 2.3 nm for the H-PC, 1D-PC, and B-PC materials, respectively. The highest BET SSA of 590 m^2^ g^−1^ was exhibited by 1D-PC. The BET analysis displayed the diameter of 250–400 nm with a 3D hierarchical structure for the H-PC nanospheres. The 3D structure was composed of macro/meso–micro/mesopores and expected to deliver the faster ion transportation and diffusion on the surface of the electrode materials and inside as well. The electrochemical performance of H-PC and H-PC@ AQS was estimated in two-electrode SC systems in 50% H_2_SO_4_ in the applied voltage window of 0 to 1.0 V at various temperatures. The H-PC based SSC device at a current density 2 mA cm^−2^ exhibited the *C*_sp_ of 81.2, 70.2, 56.6, and 54.3 mF cm^−2^ at temperatures of 0 °C, −20 °C, −40 °C, and −50 °C, respectively. At 2 mA cm^−2^, the calculated *C*_sp_ values for the SSC device based on the H-PC@AQS electrode at 25 °C, −20 °C, −40 °C, and −50 °C were 127.2, 102.4, 87.1, and 79.8 mF cm^−2^, respectively. The H-PC@AQS- and H-PC-based SSC cell configurations at 0.5 mA cm^−2^ displayed *C*_sp_ values of 185.4 and 121.5 mF cm^−2^, respectively, at room temperature. Moreover, at the temperature of −50 °C, the *C*_sp_ values for H-PC@AQS and H-PC were 89.1 and 68.9 mF cm^−2^ at a current density of 0.5 mA cm^−2^, respectively. In contrast, at 5 mA cm^−2^, H-PC@AQS and H-PC displayed the *C*_sp_ of 53.8 and 47.3 mF cm^−2^, respectively at −50 °C. According to these results, it was observed that at lower temperature, the H-PC@AQS-based SSC showed a better electrochemical performance. Further, the excellent specific areal energy of 25.7 μW h cm^−2^ was found for H-PC@AQS compared to H-PC. Thus, the incorporation of AQS in the inner micropores of H-PC resulted in an improvement in specific capacitance as well as stable performance ranging from room temperature to −50 °C. The excellent performance could be attributed to the rapid redox reactions displayed by AQS, which could be transferred to the carbon-based conducting materials. This work adds valuable insight into the design of materials for application in next-generation pseudocapacitors at a lower temperature. For practical application in SCs,^123^ the performance of the redox-active anthraquinone-2-sulfonic acid sodium (AQS)-doped conductive polymer poly(3,4-ethylenedioxythiophene) (PEDOT)-based AQS-PEDOT/PAA hydrogel electrode was tested to illuminate an LED light for a single SC at 0.8 V and in the expanded potential window of 1.6 to 2.4 V for SCs connected in series for almost 5 min (Fig. 19).^123^

#### 4.1.7. Anthraquinone polymers and covalent organic frameworks for supercapacitors

**Fig. 20:**
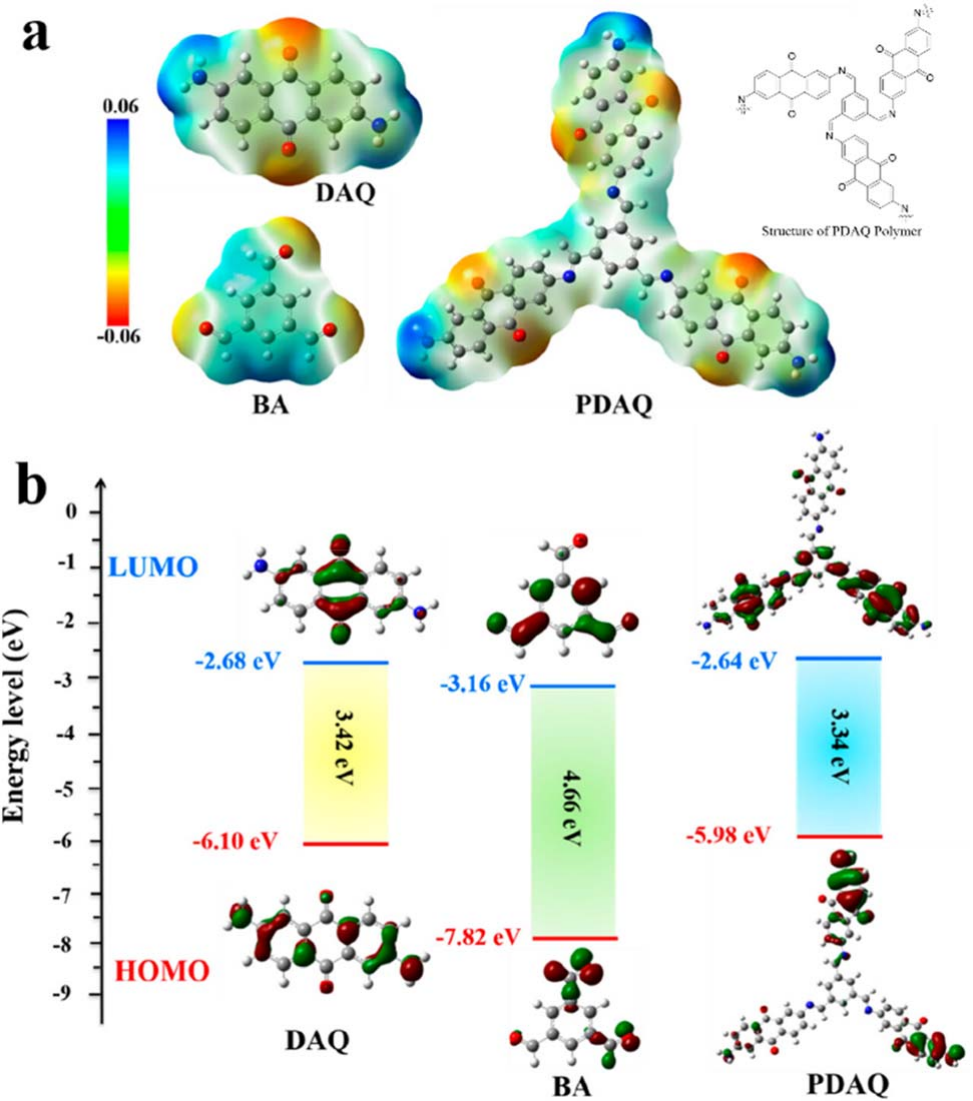
(a) MESPs of DAQ, BA, and a unit for PDAQ. (b) HOMO and LUMO distributions of DAQ, BA, and PDAQ, together with their band gaps and energies. Reproduced from ref. 129 with permission from [the American Chemical Society], Copyright [2024].

In recent years, although anthraquinone-based small organic molecule electrode materials with low cost, diverse structures, tunable redox-characteristics and environmentally friendly nature have been widely explored, the biggest challenge is the lack of larger conjugated systems.^124^ This can result in poor charge transfer capability and solubility of the active organic molecules in the electrolyte compared to polymer-based electrodes.^125^ These challenges have been tackled by researchers by incorporating redox-active small organic molecules in the polymer skeleton to form polymer electrodes.^126^ It is important to note that molecular architecture design is the most important for the design of polymers for the fabrication of electrode materials. The theoretical capacitance of polymer electrode materials can be estimated based on the active site density per unit weight of redox-active organic molecule.^127^ Anthraquinone-enriched polymers have been designed and developed to improve the performance of the fabricated electrodes. In the case of a multi-electron redox reaction, polymers bearing a high density of C=O and C=N are attractive scaffolds.^128^ This has attracted increasing attention from researchers to create new polymer-bearing anthraquinone materials as key building blocks and investigate their electrochemical performance. In this regard, Hu and co-workers reported the design, synthesis and charge storage characteristics of the PDAQ polymer.^129^ The PDAQ polymer was obtained *via* the condensation reaction between 2,6-diaminoanthraquinone (DAQ) and 1,3,5-benzenetricarboxaldehyde (BA). Initially, DFT calculations were performed to establish the electronic properties of a unit of PDAQ polymer, which could be utilized to comprehend its charge storage performance. The frontier molecular orbitals, *i.e.* HOMO and LUMO energy level, of the DAQ, BA and PDAQ polymers are shown in Fig. 20. The HOMO energy level in the PDAQ structure is delocalized on the C=O and C=N functional subunits. Therefore, the PDAQ polymer displayed a conjugation effect.^130^ In contrast, compared to the DAQ (−2.68) and BA (−3.16 eV) molecular entities, PDAQ displayed a decrease in LUMO energy level (−2.64 eV) (Fig. 20). The lower LUMO energy level of PDAQ implies an improved theoretical electron affinity.^131^ The estimated energy gap between the HOMO and LUMO level of PDAQ was 3.34 eV, suggesting the higher conductivity of the polymer compared to the individual DAQ and BA moieties.^132^ The charge storage properties of the pristine rGO, DAQ/rGO and PDAQ/rGO (with different ratio) were tested in a three-electrode SC cell using 1 M H_2_SO_4_ electrolyte. The rGO, DAQ/rGO, PDAQ/rGO-0.2, PDAQ/rGO-0.3, and PDAQ/rGO-0.5 electrodes exhibited the *C*_sp_ of 188, 325, 524, 622, and 445 F g^−1^, respectively, at 5 mV s^−1^. They observed that the PDAQ/rGO-0.5 electrode showed the highest specific capacitance among the examined electrodes, as described above. Owing to the presence of insufficient PDAQ material, the PDAQ/rGO-0.2 electrode displayed a decrease in pseudocapacitance, whereas PDAQ/rGO-0.5 with excessive PDAQ polymer enhanced the resistance for electrolyte ions to reach the electrode sample. Moreover, the GCD measurements showed the specific capacitance retention of 84% for PDAQ/rGO-0.3 after 10 000 cycles, which is superior compared to the other tested electrodes. The higher charge storage performance and cycling stability exhibited by PDAQ/eGO-0.3 could be attributed to (i) the molecular skeleton of the PDAQ π-conjugated system remaining stable when the subunits undergo reversible redox process; (ii) larger π-conjugated structure of PDAQ facilitating easier charge transfer during the charge–discharge process; (iii) the stable anchoring of PDAQ on the surface of rGO *via* π–π stacking interaction avoids the self-discharge yielded by the redox-active subunits and (iv) the 3D nanosheet network of the electrode material facilitates ion diffusion and exposes the active C=O functional groups, thus enhancing the effective use of the redox-active subunits within the PDAQ polymer and ensuring ion accessibility. Further, the authors demonstrated the fabrication of the PDAQ/rGO-0.3//DBQ/rGO ASC device and examined its charge storage properties in the applied potential voltage window of 1.8 V. The ASC devices showed 88% *C*_sp_ retention after 10 000 GCD cycles at 3 A g^−1^. The specific energy density of PDAQ/rGO-0.3//DBQ/rGO reached as high as 32.97 W h kg^−1^ at a power density of 605.57 W kg^−1^. Moreover, two tandem ASCs illuminated 63 LED lamps in an actual application. Thus, the use of these polymers for SC application expands the basis for designing new electrode materials for constructing high-performance ASCs.

**Fig. 21:**
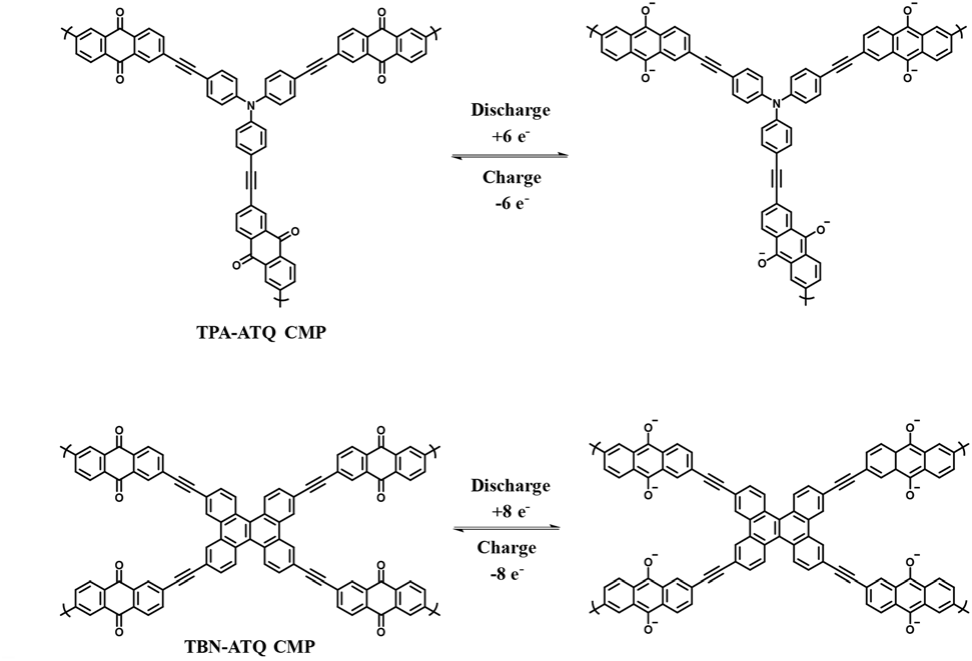
Reversible redox-mechanism of TPA-ATQ CMP and TBN-ATQ CMP.

Kuo and co-workers reported the preparation of two conjugated microporous polymers named TPA-ATQ CMP and TBN-ATQ CMP (Fig. 21) for charge storage applications.^133^ The BET characterization of the TPA-ATQ CMP and TBN-ATQ CMP electrode materials displayed type-III and type-I isotherms, respectively, using N_2_ adsorption–desorption measurements. The estimated SSA for TBN-ATQ CMP was found to be 161 m^2^ g^−1^, which is larger than that of TPA-ATQ CMP (35 m^2^ g^−1^). Between these two CMP electrode materials, TBN-ATQ CMP in a three-electrode system using 1 M KOH electrolyte in the potential window of −0.7 to 0 V displayed a *C*_sp_ of 393 F g^−1^ at a current density of 1 A g^−1^, which is higher than that of TPA-ATQ CMP (*C*_sp_ = 99 F g^−1^). The TBN-ATQ CMP retained a *C*_sp_ of 74.2% after 5000 GCD cycles. Further, in an SSC device, the TBN-ATQ CMP delivered a *C*_sp_ of 175 F g^−1^ at 1 A g^−1^ with 92.8% retention after 2000 GCD cycles. The higher electrochemical performance of the TPA-ATQ CMP could be attributed to its redox-active ATQ subunit, higher surface area and greater total pore volume (0.63 cm^3^ g^−1^), as determined by BET measurements, facilitating efficient ion transport and storage. Yang and co-workers developed phosphazene-anthraquinone-based (HD-1) covalent organic polymers (COPs) and examined their charge-storage properties.^134^ The SSA and porous structure of HD-1 were examined by means of BET analysis using N_2_ adsorption–desorption measurements at 77 K. HD-1 displayed a type-IV isotherm, suggesting its mesoporous properties. Its SSA evaluated using BET analysis was found to be 249.2 m^2^ g^−1^, suggesting that its larger SSA of HD-1 could enhance its charge-storage properties. The *C*_sp_ of the HD-1 polymer at a current density of 0.5 A g^−1^ was 125.6 F g^−1^ in 6.0 M KOH electrolyte. The SC device showed 92.8% *C*_sp_ retention of its initial value after 2000 GCD cycles. The excellent electrochemical characteristics shown by the HD-1 polymer could be attributed to the strong covalent bond connectivity and redox-active entities present in its structure.

**Fig. 22:**
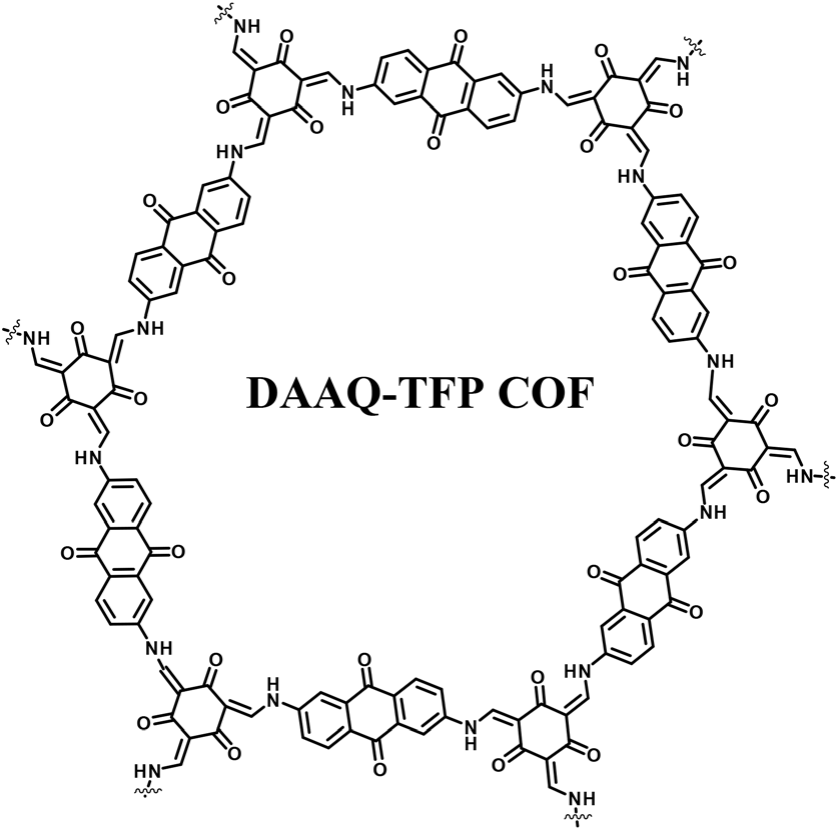
Structure of covalent organic framework of DAAQ-TFP.

To establish the charge-storage performance of anthraquinone, Dichtel and co-workers prepared the DAAQ-TFP COF covalent organic framework (COF) (Fig. 22).^135^ The BET analysis of the DAAQ-TFP COF displayed an SSA of about 1124 m^2^ g^−1^ ± 422 (average of 5 samples). The highest SSA among the examined five samples was 1800 m^2^ g^−1^, which approaches the values of 2340 m^2^ g^−1^ for the Connelly framework surface area. The estimated value was found to be higher than that of the literature reported SSA values for imine- or β-ketoenamine-linked 2D COFs.^135*b*^ The larger SSA of COFs contributes to their good electrochemical properties as electrode materials. The electrochemical performance of the DAAQ-TFP COF was compared with DAB-TFP COF bearing 1,4-diamino benzene. The DAAQ-TFP COF-modified electrode in 1 M H_2_SO_4_ electrolyte yielded a *C*_sp_ of 48 ± 10 F g^−1^, which after 10 GCD cycles stabilized at 40 ± 10 F g^−1^. Furthermore, the authors found that there was no significant decrease in specific capacitance after 5000 GCD cycles. The higher capacitance value of DAAQ-TFP COF compared to DAB-TFP COF could be ascribed to its 2D layered architecture with large-area and the presence of redox-active anthraquinone subunits. The same group fabricated thin film of DAAQ-TFP COF on an Au substrate, resulting the formation of an oriented crystalline thin film.^136^ The GCDC experiments on the DAAQ-TFP COF oriented film displayed an improvement in charge storage characteristics from 0.4 to 3 mF cm^−2^. These results indicate that controlling the COF morphology yielded the desired energy storage properties. To improve the electrical conductivity of the redox-active DAAQ-TFP 2D COF, Dichtel and co-workers electropolymerized 3,4-ethylenedioxythiophene (EDOT) within its pores.^137^ The resulting COF films incorporated with poly(3,4-ethylenedioxythiophene) (PEDOT) showed a significant enhancement in electrochemical properties as an electrode. The PEDOT-functionalized COF films exhibited a 10-fold higher current compared to the unmodified DAAQ-TFP COF film and displayed a stable specific capacitance over 10 000 cycles.

**Fig. 23:**
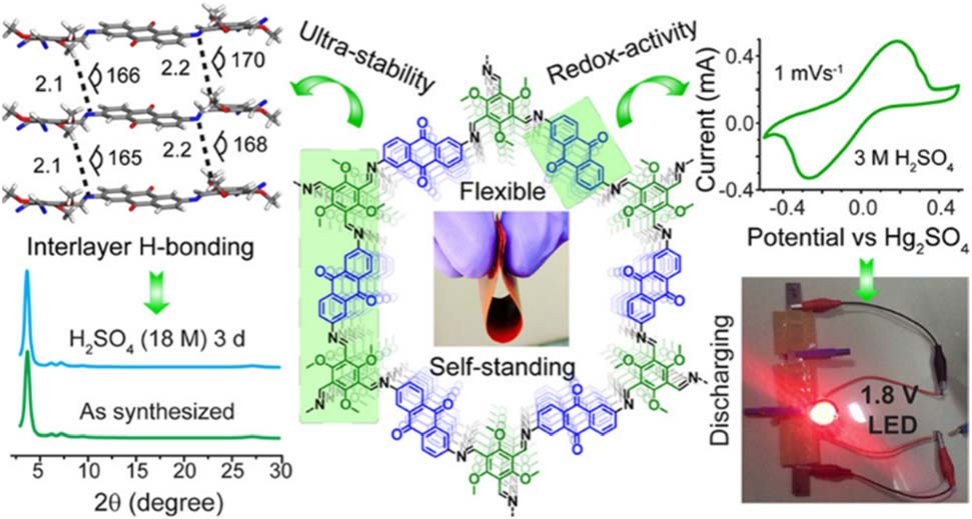
Structure of TpOMe-DAQ COF, interlayer H-bonding, XRD and illumination of LED light at 1.8 V. Reproduced from ref. 138 with permission from [the American Chemical Society], Copyright [2018].

Similarly, to tackle the sluggish electrochemical performance, Halder *et al.* reported the preparation of an imine-functionalized redox-active TpOMe-DAQ COF.^138^ The successful synthesis of the TpOMe-DAQ COF (Fig. 23) was achieved starting from small organic building blocks such as 2,4,6-trimethoxy-1,3,5-benzenetricarbaldehyde (TpOMe) and 2,6-diaminoanthraquinone (DAQ). The TpOMe-DAQ COF displayed the highest BET SSA of 1734 m^2^ g^−1^ (average 1531 m^2^ g^−1^) and the evaluated pore diameter of 2.3 nm, suggesting its mesoporous nature as an electrode material. The larger SSA and higher pore diameter play a vital role in the better electrochemical performance of the electrode by holding electrolyte ions effectively. In extreme acidic and basic electrolyte solutions such as strong acids (18 M H_2_SO_4_ and 12 M HCl) and bases (9 M NaOH), respectively, TpOMe-DAQ COF displayed ultrahigh chemical stability. This can be ascribed to the presence of interlayer C–H⋯N H-bonding between the C–H of methoxy and the ‘N’ atom of imine in the adjacent layers (Fig. 23), which was confirmed by XRD measurements in its pristine form and after treatment with 18 M H_2_SO_4_ solution for 3 days.

**Fig. 24:**
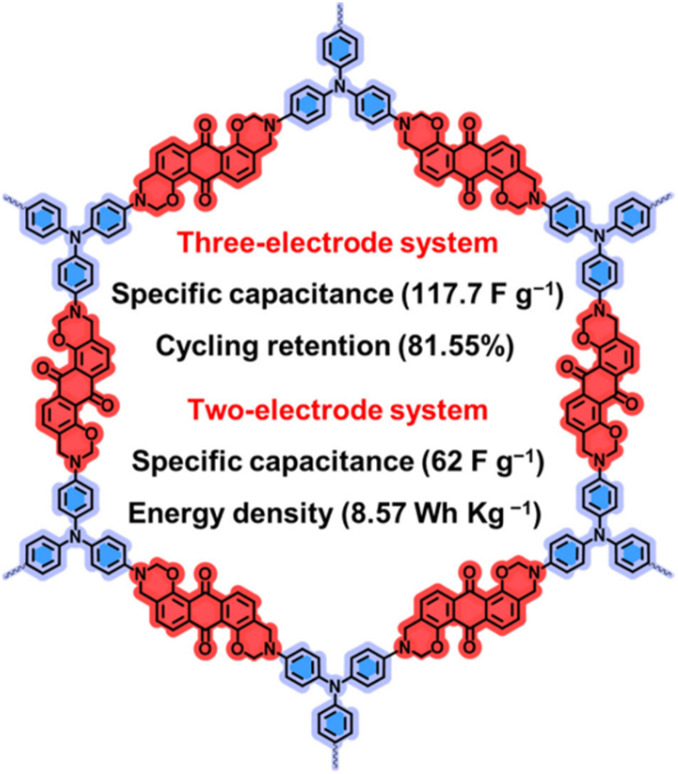
Structure of An-TPT porous organic polymer (POP) and its properties in three-electrode and two-electrode SC systems. Reproduced from ref. 140 with permission from [Elsevier], Copyright [2024].

The GCD measurement of the pristine TpOMe-DAQ COF at a current density of 3.3 mA cm^−2^ (0.35 A g^−1^) showed an increase in the areal capacitance from 1280 mF cm^−1^ (135 F g^−1^) to 1600 mF cm^−1^ (169 F g^−1^) when moving from 2 to 3 M H_2_SO_4_ electrolyte solution.^138^ The increase in areal capacitance could be due to the availability of H^+^ ions at the C=O of redox-active quinone centres. Furthermore, they demonstrated the fabrication of a symmetric solid-state supercapacitor device using 2 M aq. H_2_SO_4_/PVA gel (poly(vinyl alcohol)) as the electrolyte and two 1 cm^2^ thin sheets of pristine TpOMe-DAQ COF, which exhibited an areal capacitance as high as 84 mF cm^−2^ (8.8 F g^−1^). The solid-state SSC device also showed an energy density as high as ∼2.9 μW h cm^−2^ at a power density of ∼61.8 μW cm^−2^. The device also exhibited ∼65% capacitance retention of its initial value after 50 000 GCD cycles at a current density of 5 mA cm^−2^. Moreover, to examine its real-world application, using three solid-state devices connected in series, the authors lit an LED light at 1.8 V for 20 s (Fig. 23). These SCs provide new insight into the design and fabrication of electronic devices for modern applications. To establish the charge-storage capability of anthraquinone as an organic electrode, Lei and co-workers prepared 2D COF_DAAQ-BTA_ starting from 2,6-diamino-anthraquinone and benzene-1,3,5-tricarbaldehyde through a Schiff base condensation reaction.^139^ The as-prepared COF in combination with a graphene composite electrode displayed a *C*_sp_ of 31.7 mF cm^−2^. The CGD cycling stability experiments revealed a reduction in capacitance, which could be ascribed to the loss of the COF material and charge–discharge electrostatic repulsion, yielding poor electrical conductivity. Very recently, in 2025, Kuo and co-workers prepared a new COF material (Fig. 24) based on heteroatom-rich anthraquinone-based benzoxazine-linked porous organic polymers and showed their charge-storage properties in SC applications.^140^ The An-TPA POP in a three-electrode SC system displayed a *C*_sp_ of 117.7 F g^−1^ at 1.0 A g^−1^. After 10 000 GCD cycles, the cycling stability exerted by An-TPA POP was found to be 81.55% (Fig. 24). In a two-electrode SSC system, the An-TPA POP electrode showed the *C*_sp_ of 62 F g^−1^ at 1.0 A g^−1^ and at 1 A g^−1^, the *C*_sp_ retention of its initial value after 5000 GCD cycles was 95.71%. It is noticeable that the two-electrode SSC device also displayed an outstanding energy density (8.57 W h kg^−1^) at a power density of 500 W kg^−1^. The higher charge-storage characteristics and longer cycling stability shown by the An-TPA POP electrode could be ascribed to the presence of abundant nitrogen and oxygen heteroatoms and redox-behaviour of the organic subunits present in the POPs. The present results demonstrate the importance of POPs, enriched with heteroatoms, for next-generation SC applications, offering higher charge-storage properties and cycling stability.

The electrochemical properties of anthraquinone (AQ)-based small molecule and composite electrode materials, polymers and COFs are summarized in Table 4. Among the reported results, it was proven that the AQ-based AQ-HPCNT^108^ electrode displayed the highest *C*_sp_ of 710 F g^−1^ at 1 A g^−1^, whereas SSPANI_AQSA_1.5 exhibited the highest energy density of about 38 W h kg^−1^ at 1 A g^−1^ and 158 W kg^−1^ at 1 A g^−1^.^112^ The best characteristics for supercapacitor applications, in particular regarding energy density, was achieved by AQ derivatives. In rent years, significant efforts have been invested in the combination of polymers and COFs with redox-active AQs. The redox activity of the polymer COFs originate from the organic part of the composite electrode. Among the reported AQ-based polymers and COFs, TpOMe-DAQ delivered the highest *C*_sp_ of 1600 mF cm^−1^ (169 F g^−1^) at 3.3 mA cm^−2^ (0.35 A g^−1^), whereas the An-TPA POP showed the maximum energy density of 8.57 W h kg^−1^ at 500 W kg^−1^. Thus, the investigation of the electrochemical properties of small AQs and polymer and COF materials derived from AQs revealed that these electrodes appeared to be more suitable for use in pseudocapacitors, given that the *C*_sp_ increased upon repeated faradaic reversible redox processes.

**Table 4:** Comparison of the electrochemical properties of anthraquinone-based small molecules, polymers and covalent organic frameworks (COFs)

Compound code	Electrolyte	Type of working electrode	Specific capacitance (*C*_sp_)	Energy density (ED)	Power density (PD)	Ref.
**Anthraquinone-based small molecules**						
AQ-HPCNTs	1 M H_2_SO_4_	Three-electrode	710 F g^−1^ at 1 A g^−1^	—	—	108
1-AAQ-CC2	0.5 M Na_2_SO_4_	Two electrode SSC	328 F g^−1^ at 0.25 A g^−1^	14.8 W h kg^−1^ at 0.25 A g^−1^	240 W kg^−1^ at 0.25 A g^−1^	109
THAQ/rGO@FP	PVA/H_2_SO_4_ gel electrolyte	Two electrode	122.7 mF cm^−2^ at 0.1 mA cm^−2^	17.0 μW h cm^−2^	164.0 μW cm^−2^	110
DAQ-CGH	PVA/H_2_SO_4_ gel electrolyte	Two electrode SSC	490.2 F g^−1^ at 1 A g^−1^	36.18 W h kg^−1^ at 0.5 A g^−1^	2145.53 W kg^−1^ at 20 A g^−1^	111
SSPANI_AQSA_1.5	PVA/H_2_SO_4_ gel electrolyte	Two electrode SSC	396 F g^−1^ at 1 A g^−1^	38 W h kg^−1^ at 1 A g^−1^	158 W kg^−1^ at 1 A g^−1^	112
Bis-APDA/PANI	(EMIM^+^HSO_4_^−^) in DMF	Two electrode ASC	67 F g^−1^ at 1 A g^−1^	36.2 W h kg^−1^	995 W kg^−1^	113
DTCz-Pyz-AQ/GF	1 M H_2_SO_4_	Two electrode SSC	106.30 F g^−1^ at 0.5 A g^−1^	15.94 W h kg^−1^	899.71 W kg^−1^	117
AQ-MoS_2_	1 M H_2_SO_4_	Two electrode ASC	49 F g^−1^ at 0.1 A g^−1^	—	—	119
AZOAQ/GF	1 M H_2_SO_4_	Two electrode SSC	159.12 F g^−1^ at 0.5 A g^−1^	28.64 W h kg^−1^	1080.02 W kg^−1^	120
H-PC@AQS	50 wt% H_2_SO_4_	Two electrode SSC	127.2 mF cm^−2^ at 25 °C at 2 mA cm^−2^	25.7 μW h cm^−2^	—	122
AQS-PEDOT/PAA	1.0 M H_2_SO_4_ + 3.0 M KCl	Two electrode SSC	466.5 mF cm^2^ at 1 mA cm^2^	41.47 mW h cm^2^	400 mW cm^2^	123

**Anthraquinone-based polymers and COFs**						
PDAQ/rGO-0.3	1 M H_2_SO_4_	Two electrode ASC	—	32.97 W h kg^−1^	605.57 W kg^−1^	129
TPA-ATQ and TBN-ATQ CMP	1 M KOH	Two electrode SSC	53 F g^−1^ and 175 F g^−1^ at 1 A g^−1^	—	—	133
HD-1	6 M KOH	Three electrode	125.6 F g^−1^ at 0.5A g^−1^	—	—	134
DAAQ-TFP	1 M H_2_SO_4_	Three electrode	48 ± 10 F g^−1^ at 0.1 A g^−1^	—	—	135
PEDOT-modified DAAQ-TFP films	0.5 M H_2_SO_4_	Three electrode	350 F cm^−3^	—	—	137
TpOMe-DAQ	2 M H_2_SO_4_	Three electrode	1600 mF cm^−1^ (169 F g^−1^) at 3.3 mA cm^−2^ (0.35 A g^−1^)	—	—	138
	3 M H_2_SO_4_	Three electrode	1280 mF cm^−1^ (135 F g^−1^) at 3.3 mA cm^−2^ (0.35 A g^−1^)			
TpOMe-DAQ	2 M H_2_SO_4_/PVA	Two electrode	84 mF cm^−2^ (8.8 F g^−1^) at 0.25 mA cm^−2^	∼2.9 μW h cm^−2^	∼61.8 μW cm^−2^	138
COF_DAAQ-BTA_-3DG	1 M KOH	Three electrode	31.7 mF cm^−2^ at 0.5 mA cm^−2^	—	—	139
An-TPA POP	1 M KOH	Three electrode	117.7 F g^−1^ at 1.0 A g^−1^	—	—	140
		Two electrode	62 F g^−1^ at 1.0 A g^−1^ and at 1 A g^−1^	8.57 W h kg^−1^	500 W kg^−1^	

Therefore, quinone, naphthoquinone and anthraquinone and their derivatives afford a simple and convenient route to obtain organic redox-active electrodes for pseudocapacitive applications in SCs as well as for the illumination of LED lamps. The application of these PSCs is interesting for fabrication of wearable and portable electronic devices in the coming years. Thus, organic electrode materials will be promising candidates in the next generation of PSC real-world applications.

### 4.2. Imide derivatives

The polycyclic hydrocarbons known as rylene (R) are obtained by connecting the peri positions in naphthalene. At the rylene (R) end, two imide functional groups are present, which are called rylene diimide (RD).^141^ Between R and RD, owing to the molecular structure of RD bearing two imide functionalities exhibiting electron-withdrawing characteristics, it is the most important chromophore used in the dyestuff industry.^142^ RD displays a lower lowest unoccupied molecular orbital (LUMO), which in turn enhances its electron-accepting ability, and also its stability.^143^ Moreover, it exhibits a larger extinction coefficient, higher quantum yield and widely utilized in organic optoelectronic applications.^144^ The rylene diimide molecular structure displays advantages over the traditional optoelectronic materials due to the easy manipulation of its chemical structure and its tunable optoelectronic and electrochemical properties.^145^ Thus, rylene chromophores such as perylene-3,4,9,10-tetracarboxylic diimide (PDI) and naphthalene diimide (NDI) bearing two imide functional groups show reversible redox reactions and high electron affinity.^146^ The faradaic reversible redox-process of PDIs and NDIs make them attractive candidates for electrical energy storage (EES) devices.^147^

#### 4.2.1. Perylene diimide (PDI)-based supercapacitors

**Fig. 25:**
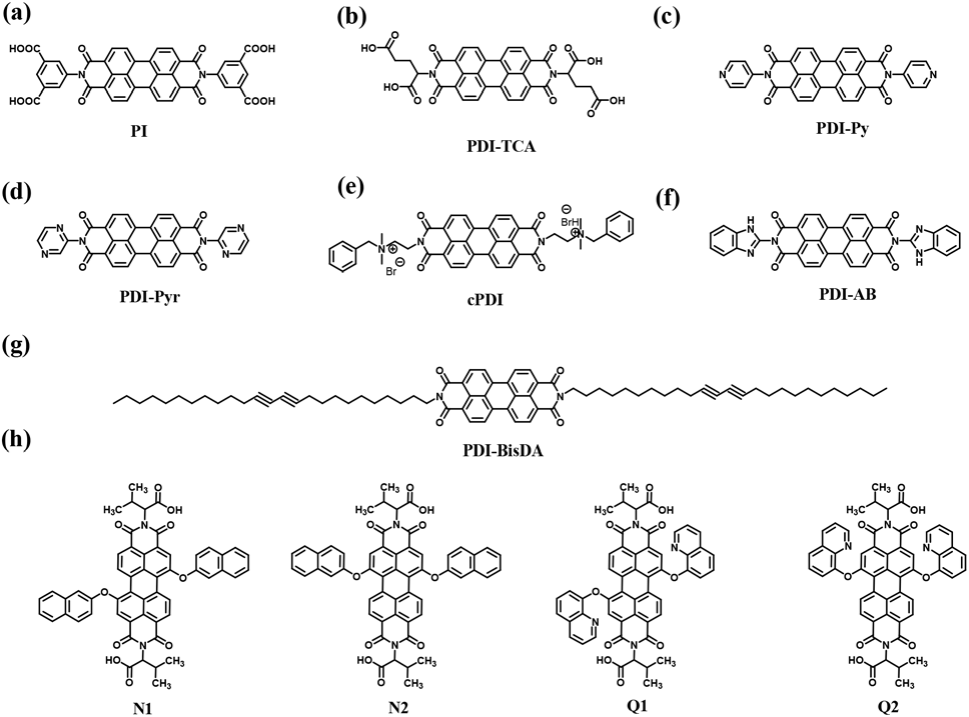
Molecular structure of PDI based small molecules (a) PI, (b) PDI-TCA, (c) PDI-Py, (d) PDI-Pyr, (e) cPDI, (f) PDI-AB, (g) PDI-BisDA, (h) N1, N2, Q1 and Q2 utilized for supercapacitor applications.

One of the higher analogues of RDs is the perylene diimide molecular entity. Given that they are inexpensive, PDI derivatives are extensively utilized in industry.^148^ Due to their reversible properties and physical and chemical stability, PDIs have been extensively investigated for use in electrochemical systems.^149^ However, although PDI and their derivatives have been employed in battery applications, their supercapacitor applications (Fig. 25) are rarely investigated. To explore the small molecular entity PDI for SC applications, Zhang and co-workers demonstrated the use of 5,5′-(1,3,8,10-tetraoxo-1,3,8,10-tetrahydroanthra[2,1,9-def:6,5,10-d′e′f′]diisoquinoline-2,9-diyl) diisophthalic acid (PI) for fabricating an active-carbon material.^150^ The obtained carbon nanofibers (CNFs) (Fig. 25a) was used as an electrode material for SC applications. The CNFs with a BET surface area of 520 m^2^ g^−1^ prepared using a non-ionic surfactant (Pluronic F-127) displayed the *C*_sp_ of 192 F g^−1^ at 1 A g^−1^. The CNF electrode retained a *C*_sp_ of 226 F g^−1^ after 1000 GCD cycles at 4 A g^−1^. The stability results are surprising. The method demonstrated here for the preparation of porous carbon could be utilized to doped different nanofibrous electrode materials with the desired pore structure and fiber diameter, which can be attractive for EES applications. Srinivasan and co-workers reported the use of perylenediimide tetracarboxylic acid (PDITCA) in SC applications (Fig. 25b).^151^ To explore PDITCA in SC application, they fabricated the PDI-doped PANI-H_2_SO_4_-PDITCA-50 electrode material using a simple preparation protocol. The BET analysis of the as-fabricated electrode materials was carried out using N_2_-adsorption and desorption isotherms. The SSA of 18.3, 21.4, and 9.0 m^2^ g^−1^ was found for the PANI-H_2_SO_4_-PDITCA-25, PANI-H_2_SO_4_-PDITCA-50, and PANI-H_2_SO_4_ materials, respectively. The larger SSA of PANI-H_2_SO_4_-PDITCA-50 provides the basis for effective ion transportation, which yielded a larger specific capacitance value. The PANI-H_2_SO_4_-PDITCA-50 composite electrode exhibited a *C*_sp_ of 460 F g^−1^ at 0.3 A g^−1^ and energy density as high as 23 W h kg^−1^ at a power density of 200 W kg^−1^. It is noticeable that the as-fabricated SC device was examined for its cycling stability, where at a current density of 3.3 A g^−1^ after 85 000 cycles, it showed 77% *C*_sp_ retention of its initial 200 F g^−1^ value. Maheshwari and co-workers derived new composite electrode materials based on perylene diimide in combination with activated carbon prepared from pineapple peel.^152^ The BET analysis of the N_2_ adsorption–desorption isotherm of the PPAC and PPAC/PDI composites displayed type II and type IV isotherms, respectively. These isotherms are related to the presence of macropores and mesopores structures in PPAC and PPAC/PDI, respectively. The BET examination of the materials exhibited the SSA of 1643, 840, 636 and 558 m^2^ g^−1^ for the PPAC, PPAC/PDI-0.5, PPAC/PDI-1 and PPAC/PDI-2 electrode materials, respectively. Moreover, the BJH analysis also indicated the presence of meso- and macropores. These hierarchical porous structures in electrode composite materials are crucial for higher charge-storage properties due to the unrestricted ion transportation and diffusion. The fabricated PDI/PPAC composite electrode was used in SC applications, which delivered a *C*_sp_ of 617 F g^−1^ at 0.5 A g^−1^. Moreover, the PPAC/PDI-1//PPAC ASC cell configuration exhibited an outstanding energy density of 62.3 W h kg^−1^ at a power density of 455 W kg^−1^ with the *C*_sp_ retention of 91.4% after 10 000 GCD cycles. The higher electrochemical characteristics of the as-fabricated ASC device could be ascribed to the effective ion diffusion and reversible faradaic redox-reactions at the electrode surface. The present investigation provides a platform to fabricate novel composite electrodes using organic PDI molecules in conjugation with carbon materials derived from the natural resources. Sheshanath Bhosale and co-workers fabricated the pyridine-functionalized perylene diimde (PDI-Py)/GF (Fig. 25c) electrode material and used it in SSC applications.^153^ The SSA of NDI-Pyr and PDI-Pyr were determined by means of BET analysis to be 1.6977 m^2^ g^−1^ and 10.015 m^2^ g^−1^, respectively. It is noticeable that PDI-Pyr displayed a higher SSA compared to NDI-Pyr. Moreover, PDI-Pyr showed a higher pore volume of 0.074 cm^3^ g^−1^ than that of NDI-Pyr (0.005 cm^3^ g^−1^). In addition, PDI-Pyr and NDI-Pyr displayed the mean pore diameter of 29.606 nm and 13.741 nm, respectively. The higher SSA, pore volume and pore diameter demonstrated by PDI-Pyr compared to NDI-Pyr indicate the superiority of the former for energy storage applications. The two-electrode PDI-Py/GF//PDI-Py/GF SSC device at a current density of 1 A g^−1^ displayed a *C*_sp_ 197 F g^−1^, which is higher than that of the NDI-pyridine based NDI-Py/GF (132 F g^−1^) electrode materials. The Py/GF electrode showed pseudocapacitive behaviour together with surface reactions in SC applications. Moreover, the PDI-Py/GF//PDI-Py/GF SSC device at 1 A g^−1^ exhibited excellent the energy density of 46 W h kg^−1^ at a power density of 3060 W kg^−1^. The PDI-Py molecular structure undergoes reversible redox-reaction changes with the contribution of four electrons and four protons, displaying pseudocapacitive behaviour as an electrode. The pyridine-functionalized PDI-based organic electrode material opens up a new molecular design basis to fabricate SSC devices with a longer cycling life. The same group investigated the pyrazine-functionalized perylene diimide (PDI-Pyr) (Fig. 25d) organic molecular structure in combination with graphite foil as an active-electrode material for SC and SSC applications.^154^ The PDI-Pyr/GF//PDI-Pyr/GF SSC device showed the excellent *C*_sp_ of 192 F g^−1^ and energy density of 54 W h kg^−1^ at a power density of 2700 W kg^−1^ and current density of 1 A g^−1^. The excellent performance could be attributed to the redox-active, *i.e.* pseudocapacitive, behaviour of pyrazine as well as PDI with the contribution of six electrons and six protons during the reversible process, availability of a higher molecular surface area, and high surface crystallinity. These results are impressive to build new molecular entities based on the redox-active heterocyclic-functionalized PDI structural architecture for next-generation SCs. The fabrication of organic material-based electrode materials *via* self-assembly can be an innovative strategy to construct binder-free electrodes for charge-storage applications over conventional electrodes. In this case, PDI (Fig. 25e) in combination with pseudocapacitive inorganic 2D functional materials such as Ti_3_C_2_T_*x*_ MXene was utilized for the preparation of hybrid electrodes in a binder-free system.^155^ In divalent electrolyte metal ions, *e.g.* Mn^2+^, Zn^2+^, and Ca^2+^, the pseudocapacitive Ti_3_C_2_T_*x*_@cPDI electrode displayed a 3-fold increase in charge-storage capacity over the Ti_3_C_2_T_*x*_ pristine electrode materials. This investigation led by Kurra and co-workers^155^ paves the new way for the development of redox-active organic–inorganic hybrid electrode architectures for the design of high-rate, high-capacity and multivalent metal-ion storage cell configurations.

**Fig. 26:**
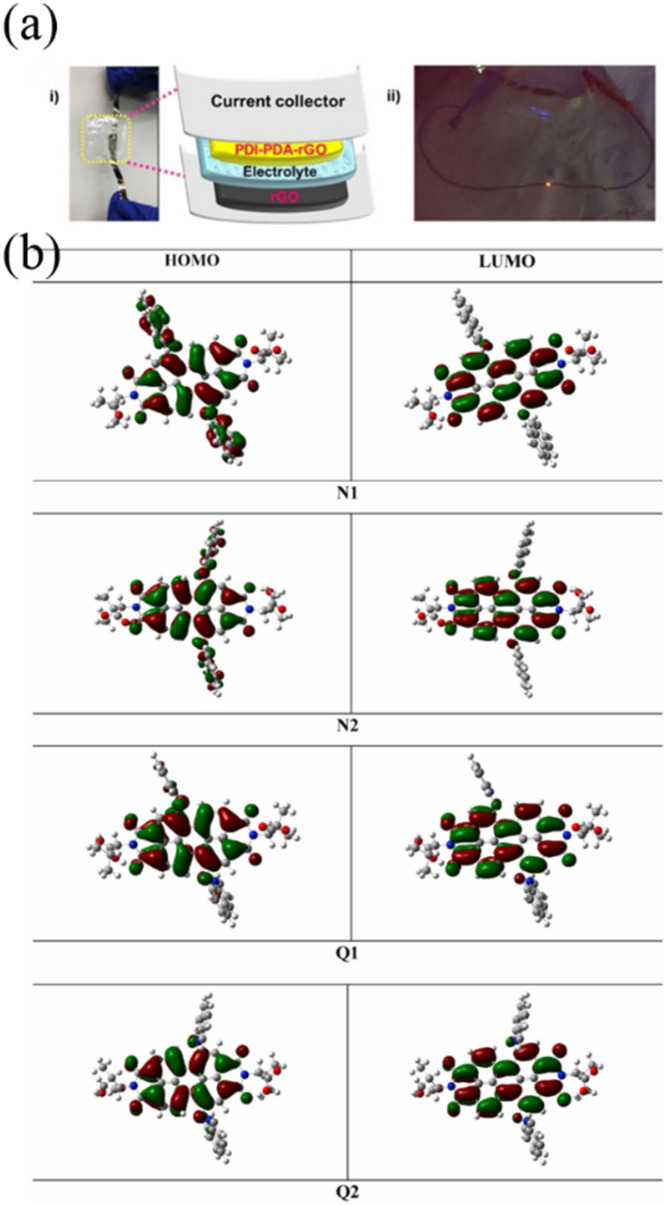
(a) (i) Schematic design and photograph of the flexible PDI-PDA-rGO//rGO ASC. (ii) Illumination of a yellow light-emitting diode using ASC. Reproduced from ref. 157 with permission from [John Wiley and Sons], Copyright [2020]. (b) Frontier molecular orbitals of N1, N2, Q1 and Q2. Reproduced from ref. 158 with permission from [Elsevier], Copyright [2025].

For the development of reversible energy storage systems, electrode materials based on organic π-conjugated donor–acceptor materials have emerged as interesting candidates due to their good electrical conductivity, excellent charge-transfer ability and high electrical dipole moment. The 2-amino-benzimidazole-substituted PDI material named PDI-AB (Fig. 25f) was developed to build active-electrode materials.^156^ Moreover, the estimated band gap of PDI-AB was found to be 2.45 eV, which is lower than that of the pristine PDI (2.99 eV). PDI-AB with an extended π-conjugation molecular structure and lower band gap yielded enhanced redox-behaviour, which is useful for enhancing the electrochemical performance of SC and SSC devices. The ITO/PDI-AB//PMMA-LiClO_4_-PC//PDI-AB/ITO SSC device at a current density of 0.5 mA g^−1^ showed a *C*_sp_ of 33.87 ± 0.66 mF g^−1^ with an energy density as high as 12.04 ± 0.23 mW h kg^−1^ at a power density of 1.6 ± 0.03 W kg^−1^. Moreover, the SSC device displayed cycling stability of 93.9% after 2000 GCD cycles. Further, the flexible SSC device exhibited the *C*_sp_ of 32.68 ± 0.44 mF g^−1^ with 11.62 ± 0.15 mW h kg^−1^ energy density at a power density of 1.6 ± 0.02 W kg^−1^. The device under white light illumination (439 mW cm^−2^) further yielded 0.8 μA W^−1^ under photocurrent generation. It is noticeable that the ITO/PDI-AB//PMMA-LiClO_4_-PC//PDI-AB/ITO flexible SSC device was successfully utilized for the illumination of a red LED light. The present investigation offers a new platform for the development of all-organic active electrode materials for lightweight flexible SSC and optoelectronic devices. Organic perylene diimde polydiacetylene (PDI-PDA) microfibers were prepared using diacetylene-functionalized PDI (PDI-BisDA) monomer (Fig. 25g) under 254 nm UV light.^157^ The PDI-PDA microfibers in combination with rGO conducting material were used to fabricate porous electrode materials. The BET examination of the PDI-PDA-rGO composite electrode material showed its higher SSA and pore size compared with that of the PDI-PDA microfiber, suggesting that this material is useful to achieve higher charge-storage characteristics. PDI-PDA played a key role in enhancing the electrochemical characteristics of the SC and ASC devices. It was observed that the PDI-PDA/rGO electrode in a three-electrode SC cell configuration displayed the outstanding *C*_sp_ of 610 F g^−1^ at 1 A g^−1^. In contrast, the two-electrode ASC device exhibited the *C*_sp_ of 310 F g^−1^ at 1 A g^−1^ together with a longer cycling life and high power density. A functional SC device was fabricated, which could successfully light a yellow LED lamp at 1.6 V (Fig. 26a).^157^ This study confirms the potential utility of this electrode as a charge-stage platform.

Besides imide substitution in the PDI molecular structure, bay-substituted PDIs are interesting electrode materials for supercapacitor applications. In this case, Durga and co-workers successfully synthesised four molecular scaffolds, N1, N2, Q1 and Q2 (Fig. 25h), *via* the structural manipulation of perylene diimde at its bay-positions.^158^ They performed DFT calculation for designing these molecular structures. The HOMO and LUMO frontier molecular orbitals of N1, N2, Q1 and Q2 are displayed in Fig. 26b.^158^ The HOMO energy level was delocalized over the PDI core together with 1,6- and 1,7-susbtitutions of N1 and N2, respectively. The energy level distribution implies the HOMO to LUMO charge transfer process takes place *via* the movement of charges from the substituent moieties to the PDI central core. According to the HOMO and LUMO energy levels distribution, the strong delocalization of electrons occurs inside the system. The estimated HOMO/LUMO energy level values are −6.136/−3.834 eV, −6.194/−3.827 eV, −5.893/−3.596 eV and −5.930/−3.610 eV for N1, N2, Q1 and Q2, respectively.^158^ The calculated band gap for N1, N2, Q1 and Q2 molecules is 2.302, 2.367, 2.297 and 2.320 eV, respectively. The 1,7-regioisomers display slightly higher band gap values compared to the 1,6-regioisomers. The SSC device based on Q1 (cell-3) in 1 M H_3_PO_4_ electrolyte solution exhibited the *C*_sp_ of 146.54 F g^−1^ (*via* impedance analysis at 10 mHz) and 118.33 F g^−1^ (*via* CV at 5 mV s^−1^). The as-fabricated PDI exhibiting redox-active characteristics with energy generation and charge-storage capability can be utilized in hybrid devices including photo-capacitor and photo-battery applications.

**Fig. 27:**
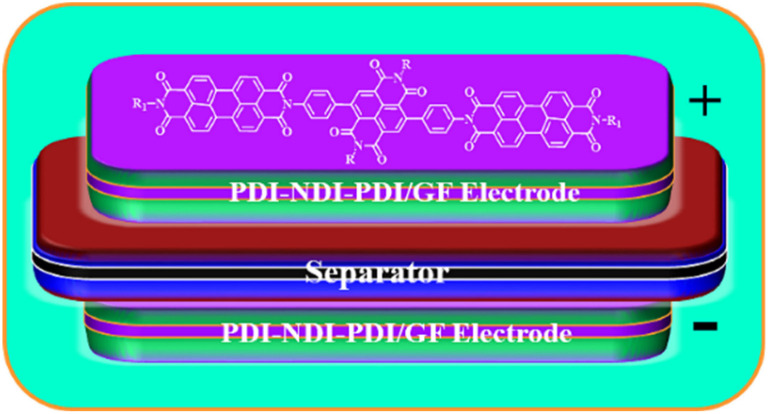
Schematic presentation of symmetric supercapacitor device based on the PD-NDI-PDI/GF electrode system. Reproduced from ref. 159 with permission from [Elsevier], Copyright [2024].

Very recently, we explored the importance of the hybrid molecular architecture based on perylene diimide and naphthalene diimide in electrochemical applications.^159^ The BET analysis based on the nitrogen adsorption–desorption isotherms of PDI-NDI-PDI displayed an SSA of 3.810 m^2^ g^−1^, pore volume of 0.01680 cm^3^ g^−1^ and pore diameter of 19.06 nm, suggesting that the as-fabricated electrode material is suitable to enhance the charge-storage properties of the supercapacitor cell configurations. The as-fabricated PDI-NDI-PDI/GF electrode was used in SC and SSC applications (Fig. 27). The electrode displayed pseudocapacitive behaviour due to its faradaic reversible redox-reactions. The SSC device based on PDI-NDI-PDI/GF as the anode and cathode in 1 M H_2_SO_4_ electrolyte in the applied voltage window of −0.2 to 1.0 V at 0.5 A g^−1^ current density exhibited the *C*_sp_ of 193.33 F g^−1^ with the excellent energy density of 34.80 W h kg^−1^ at the power density of 1079.98 W kg^−1^. The present investigation paves the way for the design of n-type electrode materials based on two different redox-active imide systems.

#### 4.2.2. PDI-polymer supercapacitors

**Fig. 28:**
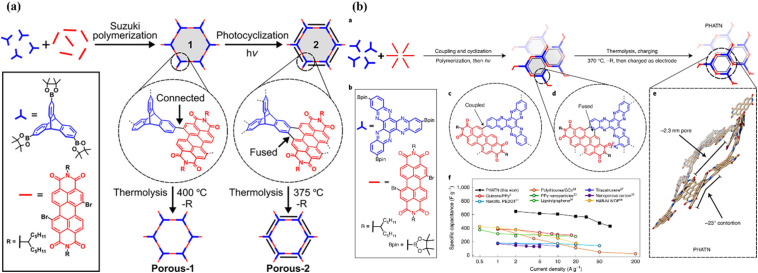
(a) Schematic illustration of the method for the preparation and use of PDI-triptycene to construct Porous-1 and Porous-2 isomers. Reproduced from ref. 164 with permission from [the American Chemical Society], Copyright [2018]. (b) Organic building blocks and their use for the construction of PHATN, their DFT and *C*_sp_*vs.* current density and comparison with materials reported in the literature. Reproduced from ref. 165 with permission from [Springer Nature], Copyright [2021]. (For detailed references refer to https://doi.org/10.1038/s41563-021-00954-z).

**Fig. 29:**
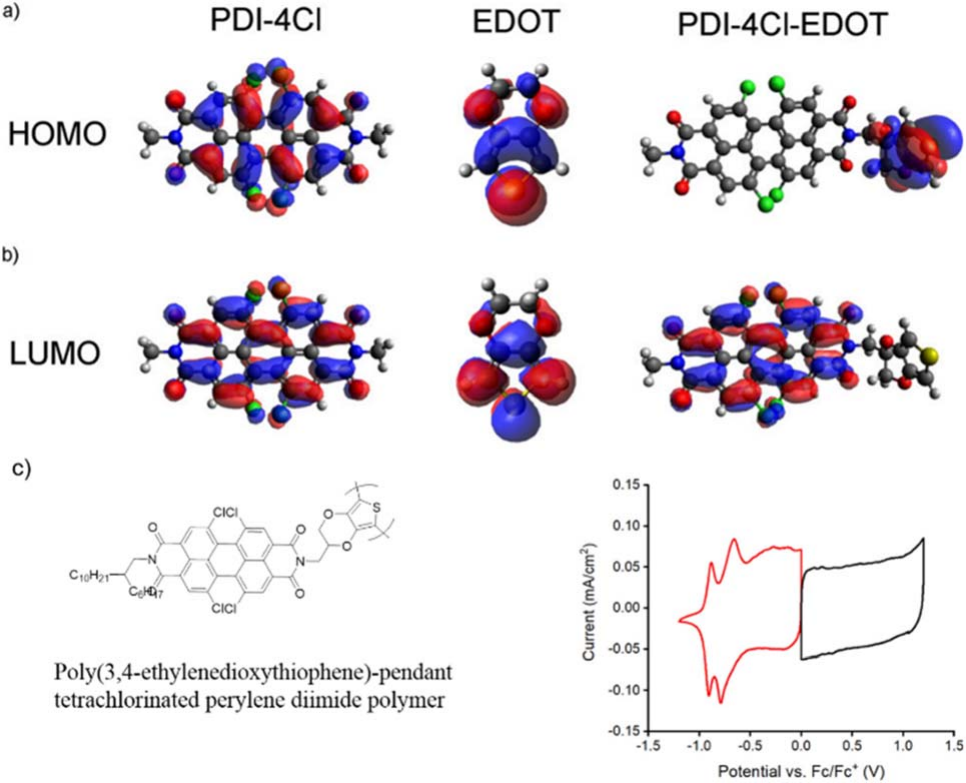
(a and b) Frontier molecular orbital diagrams of the PDI-4Cl core, EDOT monomer and PDI-4Cl-EDOT monomer. (c) Polymer derived from PDI-4Cl-EDOT monomer. (d) CV of PEDOT/PDI polymer film in CAN with 0.1 M TBAPF_6_ at 10 mV s^−1^ sweep rate. Reproduced from ref. 166 with permission from [the American Chemical Society], Copyright [2020].

Presently, owing to the advancement of energy storage technology, it is necessary to develop conjugated polymer materials derived from redox-active small organic molecules capable of storing and rapidly distributing energy. Pseudocapacitors based on these redox-active conjugated polymers store charges *via* faradaic reversible redox-reactions.^160^ Polymeric materials based on electron-donor and acceptor-properties are required to achieve the widest potential voltage window to fabricate pseudocapacitive devices.^161^ The donor (p-type)^162^ and acceptor (n-type)^163^ moieties display charge storage through oxidative and reductive process, respectively. Nuckolls and co-workers designed and successfully synthesized porous materials, *i.e.* Porous-1 or Porous-2, derived from perylene diimide (PDI) and triptycene organic subunits (Fig. 28).^164^ To investigate their charge-storage performance, they fabricated Porous-1/Ni foam and Porous-2/Ni foam electrodes using carbon black and polytetrafluoroethylene (Fig. 28a).^164^ The electrochemical performance of the Porous-1/Ni foam and Porous-2/Ni foam electrodes was investigated in 1 M Na_2_SO_4_ electrolyte solution. The Porous-1/Ni foam electrode displayed pseudocapacitance with the *C*_sp_ and capacity of 350 F g^−1^ and 59 mA h g^−1^, respectively, at 0.2 A g^−1^, which are higher compared to that of the Porous-2/Ni foam electrode. Alternatively, the Porous-2/Ni foam electrode showed a higher *C*_sp_ than that of the Porous-1/Ni foam electrode above 1 A g^−1^, *i.e.* a higher current density. The cycling stability displayed by these electrodes was very high for >10 000 GCD cycles. These electrochemical results establish the polymeric material prepared from electron-accepting redox-active organic scaffolds as a powerful tool for constructing next-generation tunable energy-storage devices. In 2021, the same group demonstrated a new polymeric PHATN (perylene diimide-hexaazatrinaphthylene) framework derived from perylene diimide (PDI) and hexaazatrinaphthylene (HATN) (Fig. 28b).^165^ The BET analysis using CO_2_ adsorption and desorption isotherms at −78 °C for the contorted PHATN displayed an SSA of 131 m^2^ g^−1^, which is higher compared to that of PA-PDI of 12 m^2^ g^−1^. The contortion of PHATN enhanced the accessible surface area of the electrode material to facilitate ion diffusion, which is crucial to enhance the electrochemical properties of pseudocapacitive devices. The as-fabricated polymeric framework showed an excellent performance with *C*_sp_ of 689 F g^−1^ at 0.5 A g^−1^ and long cycling life of over 50 000 cycles. The present PHATN framework undergoes reversible redox-process through the involvement of 4 electrons. The pseudocapacitive nature of the SC device could be attributed to the combination of the complementary redox-active organic subunits PDI and HATN in the PHATN electrode materials. The molecular contortion of PHATN facilitated the faster diffusion of the electrolyte ions and long-range charge-distribution. These electrochemical results provide the basis for the design of organic high-performance charge-storage materials with great potential in next-generation SC systems. The fabrication of low-cost pseudocapacitive polymers is an attractive alternative to the pseudocapacitive metal oxide materials. The SC devices derived from these polymeric materials can offer high energy density and good stability. To achieve higher energy density pseudocapacitive electrode materials, the development of polymeric materials with a wider voltage window and store charge with the involvement of positive and negative charge storage capability, is necessary. Seferos and co-workers developed the PDI-4Cl-EDOT monomer from PDI-4Cl core and EDOT monomer based on their HOMO and LUMO energy levels (Fig. 29) according to DFT calculations, which were performed using the B3LYP functional and 6-31G(d) basis set.^166^ The hybrid alkyl chain of PDI-4Cl-EDOT was truncated to a methyl change to perform the calculations. The HOMO (Fig. 29a) and LUMO (Fig. 29b) energy levels are delocalised on the EDOT and PDI-Cl core, respectively, of PDI-4Cl-EDOT.^166^ The HOMO and LUMO energy levels indicate that the positive charge could be stored on the PEDOT backbone, whereas the negative charge could be stored by the tetrachlorinated PDI subunit in the PDI-4Cl-EDO monomer. According to the DFT calculations, the molecular structure of PDI-4Cl-EDOT demonstrates that it is a donor–acceptor type of the monomer, which is converted into a donor–acceptor polymer.^167^ The poly(3,4-ethylenedioxythiophene)-pendant tetrachlorinated perylene diimide polymer (PEDOT/PDI polymer) (Fig. 29c) was prepared starting from the PDI-4Cl-EDOT-O12 monomer *via* oxidative electrochemical polymerization.^167^ The PEDOT/PDI polymer was capable of storing positive and negative charges, which was supported by performing CV measurements separately in both the positive and negative regions (Fig. 29d).^166^ At a current density of 0.5 A g^−1^, the PEDOT/PDI polymer film exhibited a *C*_sp_ of 78.6 F g^−1^ and 73.1 F g^−1^, in the positive and negative regions, respectively.^166^ In the SSC device using a gel polymer electrolyte, the PEDOT/PDI polymer at a current density of 1 A g^−1^ in the applied potential voltage window of 2.2. V showed an energy density as high as 8.95 W h kg^−1^ at a power density of 76.8 kW kg^−1^.^166^ These electrochemical results based on the polymer bearing donor-node-acceptor architecture can provide the basis for designing ambipolar polymers exhibiting longer cycling stability.

**Fig. 30:**
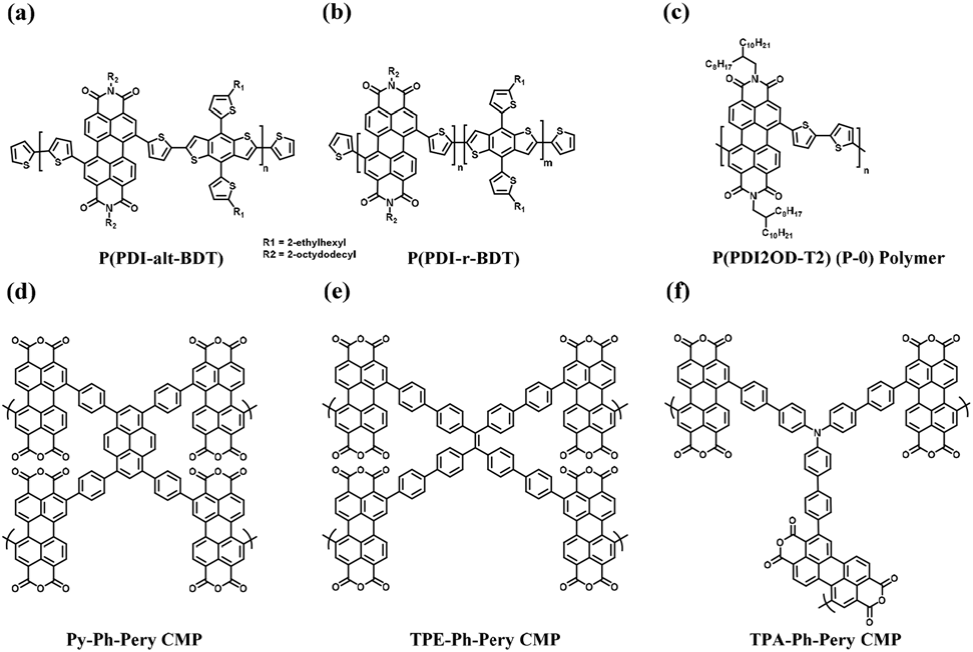
PDI based polymer structures (a) P(PDI-*alt*-BDT), (b) P(PDI-r-BDT), (c) P(PDI2OD-T2) (P-0) polymer, (d) Py-Ph-Pery CMP, (e) TPE-Ph-Pery CMP and (f) TPA-Ph-Pery CMP.

The π-conjugated polymers (Fig. 30) based on donor-accepter subunits provide an exciting possibility of charge-storage properties involving positive and negative electrodes. The presence of donor and acceptor subunits can enhance the operating voltage window, resulting in a higher energy density and higher specific capacitance together with higher power density. Shrama *et al.* demonstrated the synthesis of donor–acceptor π-conjugated polymers P(PDI-*alt*-BDT) (Fig. 30a) and P(PDI-*r*-BDT) (Fig. 30b) based on perylene diimide (PDI) as an acceptor and its donor complementary subunit benzodithiophene (BDT).^168^ The as-fabricated composite electrodes were utilized in type III SCs. Compared to its P(PDI-*r*-BDT) and NDI-based polymer counterparts, P(PDI-*alt*-BDT) in a single-electrode setup in the organic electrolyte PC-LiClO_4_ exhibited *C*_sp_ of 113 F g^−1^ at 0.5 A g^−1^ with 100% retention of its initial value after 4000 GCD cycles. Moreover, the device displayed an energy density of 9.1 W h kg^−1^ at a power density of 82 kW kg^−1^. Further, the flexible device displayed a higher areal capacitance of 35 mF cm^−2^ at 0.5 mA cm^−2^ compared to similar donor–acceptor π-conjugated polymers reported in the literature. Finally, the device was successfully applied for its practical utility to light an LED lamp at 2.5 V. The same group reported the fabrication of the P(PDI2OD-T2)/MWCNT composite electrode using the donor–acceptor π-conjugated polymer P(PDI2OD-T2) (Fig. 30c) and its use in SSC applications in the presence of liquid organic (LE-P-2‖P-2) and quasi-solid-state gel (QSS-P-2‖P-2) electrolytes.^169^ N_2_ adsorption/desorption isotherms were recorded to demonstrate the pore size and SSA of the MWCNT, P-0 and P-2 polymers. The pore size distribution of P-0 was found to be predominantly microporous, whereas MWCNT displayed both micro-/mesopores. It was observed that as the proportion of MWCNT increased in the P-2 polymer, the mesoporous area increased compared to P-0. All three materials, P-0, P-2, and MWCNT, showed type III isotherms. The estimated SSA area of MWCNT, P-0 and P-2 was found to be 206.0 m^2^ g^−1^, 11.3 m^2^ g^−1^ and 84.2 m^2^ g^−1^, respectively. The *in situ* incorporation of MWCNT increased the surface area of the P-2 polymer composite compared to P-0. The authors claimed that the higher SSA and hierarchical porous morphology easily allowed the transport of the electrolyte ions, increasing the capacity of the electrode for charge storage. At a current density of 0.25 A g^−1^, the P(PDI2OD-T2)/MWCNT//P(PDI2OD-T2)/MWCNT SSC device in (LE-P-2‖P-2) and flexible quasi-solid-state device in (QSS-P-2‖P-2) electrolytes in the operating voltage window of 3.1 V exhibited the estimated *C*_sp_ values of 85.4 and 84.2 F g^−1^, respectively. The SSC device showed 70% *C*_sp_ retention after 45 000 GCD cycles. The flexible cell configuration exhibited the outstanding energy density of 112.4 W h kg^−1^ at a power density of 18 600 W kg^−1^. These results suggest the practical applicability of these polymeric electrode materials in real-world applications.

**Fig. 31:**
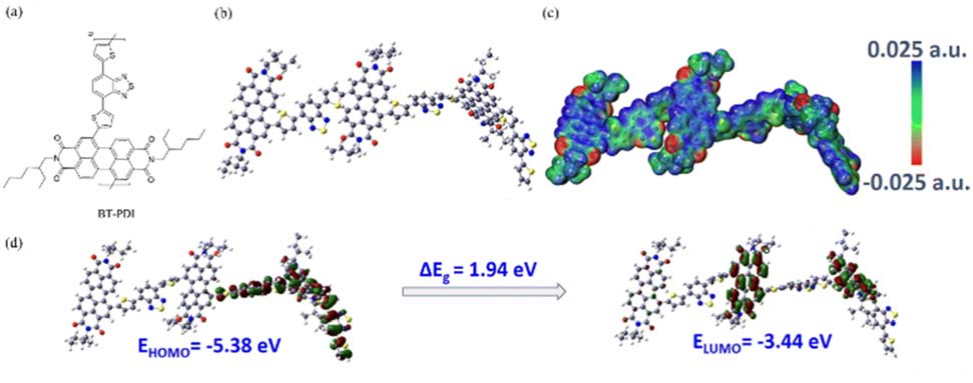
(a) Molecular structure of BT-PDI polymer; (b) optimized structure of PDI-BT-PDI trimer; (c) MSEP of PDI-BT-PDI and (d) frontier molecular structures of PDI-BT-PDI trimer. Reproduced from ref. 174 with permission of RSC.

Porous organic polymers bearing π-conjugation are termed conjugated microporous polymers (CMPs).^170^ The availability of diverse small organic π-conjugated building blocks and their simple preparation method make them attractive scaffolds. CMPs are efficient for the fabrication of electrode materials and their application in electrical energy storage devices, *e.g.* SCs. The CMPs constructed using redox-active small organic molecular architectures display interesting properties such as (i) faradaic reversible redox reactions, (ii) insoluble in the aqueous electrolyte, (iii) high cycling stability and (iv) high energy density. Kuo and co-workers developed three CMPs based on PDIs in combination with pyrene (Py), tetraphenylethylene (TPE) and triphenyl amine (TPA) to yield Py-Ph-Pery (Fig. 30d), TPE-Ph-Pery (Fig. 30e), and TPA-Ph-Pery (Fig. 30f), respectively, *via* a Suzuki–Miyaura coupling reaction.^171^ N_2_ adsorption and desorption tests of Py-Ph-Pery, TPE-Ph-Pery, and TPA-Ph-Pery CMPs at 77 K were carried out to estimate their DDA, total pore volume and pore size diameters. The as-prepared Py-Ph-Pery, TPE-Ph-Pery, and TPA-Ph-Pery CMPs showed detectable hysteresis, indicating the presence of mesoporous framework structures. Moreover, these CMPs displayed type-II and type-IV isotherm curves, suggesting their porous structure. The SSA values of Py-Ph-Pery, TPE-Ph-Pery and TPA-Ph-Pery were 656 m^2^ g^−1^, 16 m^2^ g^−1^ and 12 m^2^ g^−1^, respectively. The total pore volumes of 0.09 cm^3^ g^−1^, 0.05 cm^3^ g^−1^ and 0.04 cm^3^ g^−1^ were observed for Py-Ph-Pery, TPE-Ph-Pery and TPA-Ph-Pery, respectively. In addition, the pore diameter of 1.78–2.3, 2.34, and 3.19 nm was obtained for Py-Ph-Pery, TPE-Ph-Pery, and TPA-Ph-Pery CMPs, respectively. The nanoscale pore size suggests the presence of mesoporosity in the Pery-CMP frameworks. They utilized a three-electrode and symmetric coin cell configuration to evaluate the performance of the electrodes based on Py-Ph-Pery, TPE-Ph-Pery, and TPA-Ph-Pery. At a current density of 0.5 A g^−1^, the SC device in 1.0 M KOH aqueous solution delivered the *C*_sp_ of 300, 82 and 68 F g^−1^ for Py-Ph-Pery, TPE-Ph-Pery and TPA-Ph-Pery CMPs, respectively. In the case of Py-Ph-Pery CMP, TPE-Ph-Pery CMP, and TPA-Ph-Pery CMP, their energy density values were observed to be 41.6, 11.3, and 9.44 W h kg^−1^, respectively. In the symmetric coin cell configuration at 1 A g^−1^, the *C*_sp_ of Py-Ph-Pery, TPE-Ph-Pery and TPA-Ph-Pery CMPs was observed to be 84, 26, and 23 F g^−1^, respectively. The energy densities of 23.33, 7.32, and 6.49 W h kg^−1^ were displayed by the Py-Ph-Pery, TPE-Ph-Pery and TPA-Ph-Pery CMPs, respectively. The analysis of the charge-storage properties revealed that the electrode materials displayed a combination of EDLC and pseudocapacitive behaviour. These results indicate that the as-synthesized CMPs bearing redox-active organic small molecules are promising electrode materials for SC applications. The present investigation paves a new way to utilize small organic molecules in the development of new CMPs for next-generation supercapacitors. The lowering of the energy band gap, faster ion diffusion, widening of the operational potential voltage window and electroactive surface of the electrode material could be achieved using the combination of p-type and n-type moieties in π-conjugated donor–acceptor-type polymers.^172,173^ These π-conjugated polymers are impressive for charge-storage applications. To explore these polymers for SC applications, Park and co-workers prepared two benzothiadiazole (BT)-functionalized polymers named BT-NDI and BT-PDI based on naphthalene diimide (NDI) and perylene diimide (PDI), respectively.^174^ The molecular structure, optimized geometry, corresponding molecular electrostatic potential (MESP) map and frontier molecular orbitals of BT-PDI are displayed in Fig. 31. The deviation from the 180° dihedral angle between BT and PDI makes BT-PDI (Fig. 31a) less linear and planar (Fig. 31b). According to the MSEP map (Fig. 31c), it was observed that although the thiophene moiety is present in the molecular structure of BT, it shows strong-electron-withdrawing nature, indicating its low positive electrostatic potential (greenish-blue color). In addition, PDI also displays a greater positive potential (blue color in the MSEP map). The peripheral PDI atoms display a slightly greenish-blue color, suggesting their lower positive potential. These MSEP observations for the BT-PDI oligomer imply an increase in π-conjugation along the BT-PDI polymer backbone, resulting in a higher electrochemical performance.^175^ Moreover, the HOMO (−5.38 eV) of the PDI-BT-PDI trimeric subunit exhibited localization of the electron density across the BT subunit, whereas the LUMO (−3.44 eV) energy level was delocalized over the PDI subunits (Fig. 31d). This could enhance the intramolecular charge transfer properties from the BT to PDI subunits. The estimated energy band gap was found to be 1.94 eV in the trimeric PDI-BT-PDI moiety, indicating an increase in electronic conductivity depending on the degree of the polymerization of the BT-PDI oligomer.^174^ The polymeric BT-PDI-based electrode displayed the *C*_sp_ of 196 F g^−1^ at 1 A g^−1^, where its higher specific capacitance could be attributed to its porous structure and lower energy band gap. The SC device showed the *C*_sp_ retention of 76% with respect to is initial value after 5000 GCD cycles at 5 A g^−1^. The SSC devices based on the BT-PDI polymer in organic electrolyte in the applied voltage window of 0–3 V showed an excellent energy density of 52.9 W h kg^−1^ at a power density of 2.9 kW kg^−1^ at a current density of 1 A g^−1^. Moreover, at a higher current density of 5 A g^−1^, a maximum power density of 14.9 kW kg^−1^ was found with an energy density of 15.82 W h kg^−1^, indicating the excellent supercapacitive behavior of the polymeric electrode.^174^ The obtained results based on the BT-PDI polymer were superior to that of the BT-NDI polymer-based electrode in SC configurations. Thus, donor–acceptor polymers are attractive electrode materials, which can be utilized in a high voltage window with better stability and overall better electrochemical performance, suggesting the significance of newer polymeric designs for SC applications.

**Fig. 32:**
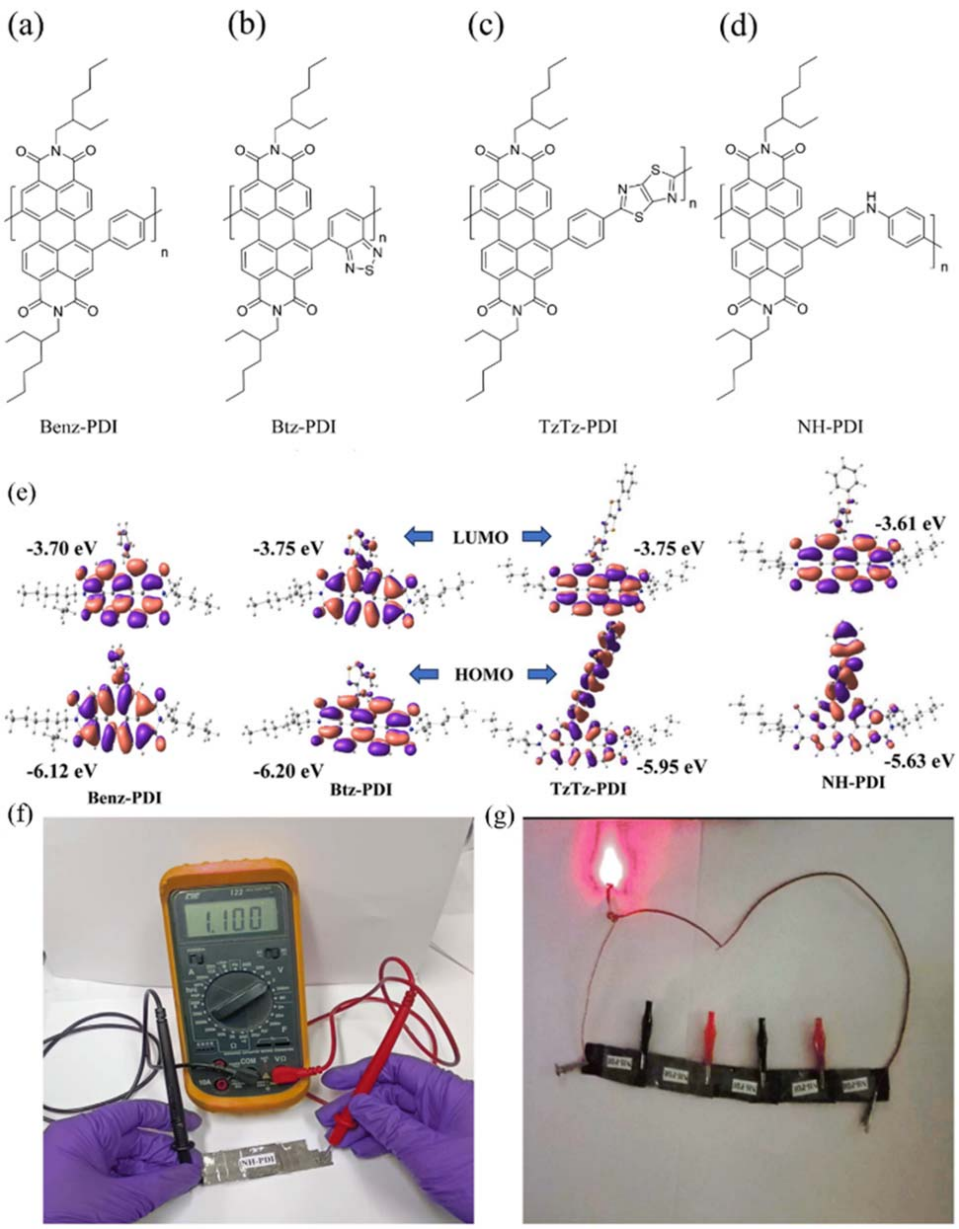
Polymer structures of (a) Benz-PDI, (b) Btz-PDI, (c) TzTz-PDI and (d) NH-PDI; (e) frontier molecular orbitals of Benz-PDI, Btz-PDI, TzTz-PDI and NH-PDI, (f) charged state of a single solid-state SC device and (g) illumination of an LED lamp by connecting five consecutive SC cells at 3.0 V. Reproduced from ref. 176 with permission from RSC.

Similarly, Malik and co-workers demonstrated the synthesis and electrochemical properties of four PDI-based polymers (Fig. 32) including Benz-PDI (Fig. 32a), Btz-PDI (Fig. 32b), TzTz-PDI (Fig. 32c) and NH-PDI (Fig. 32d) with increasing strength of donor–acceptor characteristics.^176^ The redox properties of these polymers were found to change with an enhancement of the donor–acceptor strength. The geometry optimizations were performed with the Becke–Johnson dispersion-corrected B3LYP functional (B3LYP-GD3BJ) and 6-31+g(d) basis set, and DFT calculations were carried out to establish the geometry optimization of the Benz-PDI, Btz-PDI, TzTz-PDI and NH-PDI polymers. As demonstrated in Fig. 32e, the HOMO energy level is localized over both PDI and linker subunits, whereas the LUMO energy level is delocalized over the PDI moieties only. The calculated energy gaps are 2.42, 2.44, 2.20 and 2.01 eV for Benz-PDI, Btz-PDI, TzTz-PDI and NH-PDI, respectively.^176^ In the case of NH-PDI, a lower energy gap was observed, suggesting prominent intramolecular charge transfer between its donor and acceptor subunits. Among the investigated polymers, at 0.5 A g^−1^, NH-PDI exhibited highest *C*_sp_ of 363 F g^−1^ and 134.2 F g^−1^, for the three-electrode SC (0.5 M H_2_SO_4_ electrolyte) and SSC devices (PVA + H_2_SO_4_ (1 : 1) gel electrolyte), respectively. Moreover, the SSC device achieved an energy density of 22.5 W kg^−1^ at a power density of 274.8 W kg^−1^ and 0.5 A g^−1^. The NH-PDI solid-state SC device in its charged state is shown in Fig. 32f. To explore the practical applicability of the NH-PDI electrode material, five consecutive solid-state SC configurations were connected in series to illuminate an LED light (Fig. 32g) at 3.0 V for 3 min after charging the device. These results indicate that the bay-substituted NH-PDI polymer with donor–acceptor characteristics is a suitable pseudocapacitive electrode material for high-performance SCs.

#### 4.2.3. PDI-MOF supercapacitors

**Fig. 33:**
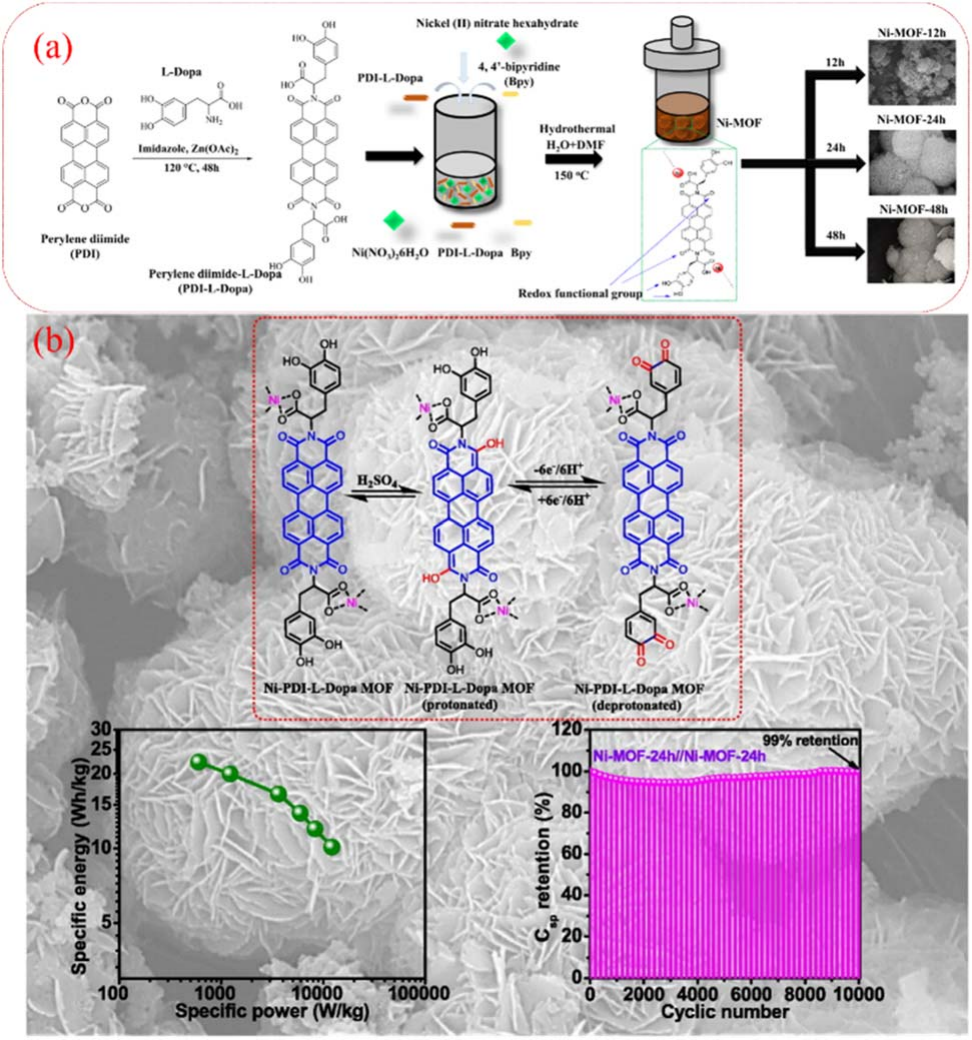
(a) Schematic illustration of the preparation of Ni-MOF, (b) redox-mechanism of Ni-MOF and Ragone plot and cycling stability. Reproduced from ref. 178 with permission from [Elsevier], Copyright [2023].

In recent years, metal organic frameworks (MOFs) generated from the incorporation of metal ions/metal complexes in organic ligands have been utilized for various applications, *e.g.* catalysis, gas storage and separation, wearable sensors, chemical/biological sensors, batteries and supercapacitors.^177^ Compared to conventional polymers, MOFs display unique characteristics such as porosity, tunable pore size, and high surface area, which provide newer possibilities of electrode materials for developing high-performance supercapacitors. In this regard, manipulation of the organic ligands and metal ions in MOFs offers improved surface charge density, dielectric constant and charge distribution of the electrode materials. Although several examples have been reported in the literature on MOF-based electrodes for SC applications, research on PDI-based MOFs is in its infancy and requires further investigation in this field. Kale *et al.* demonstrated the synthesis of Ni-MOF and its SC applications (Fig. 33).^178^l-dopa-functionalized perylene diimide (PDI) named PDI-l-Dopa was treated with nickel nitrate hexahydrate to yield the Ni-MOF architecture (Fig. 33a). The BET analysis of the N_2_ adsorption–desorption measurements showed the typical type-IV isotherms for the Ni-MOF-12 h and Ni-MOF-48 h samples. The hysteresis loop suggests that the samples are mesoporous in nature. The maximum SSA of 132.80, 55.12 and 52.38 m^2^ g^−1^ was found for Ni-MOF-24 h, Ni-MOF-12 h and Ni-MOF-48 h, respectively. The SSA of Ni-MOF-24 h is larger than that of the other two samples. In addition, the pore volume of the Ni-MOF-24 h, Ni-MOF-12 h and Ni-MOF-48 h electrode materials was found to be 0.3977, 0.2061 and 0.1502 cm^3^ g^−1^, respectively. It has been well documented that a larger SSA indicates a larger number of active sites available for interacting with the electrolyte ions. Moreover, a mesoporous nature with larger pore volume facilitates the transportation of the electrolyte ions. Thus, the higher SSA and larger pore volume of the Ni-MOF-24 h mesoporous materials helped increase its charge-storage capability as an electrode material.^178*b*^ The flower-like microspheres of Ni-MOF displayed better electron transport, resulting in higher conductivity. At 1 A g^−1^, in the three-electrode configuration, Ni-MOF-24 h yielded the *C*_sp_ of 198 F g^−1^. The Ni-MOF-24 h electrode in the Ni-MOF-24 h//Ni-MOF-24 h SSC system delivered the *C*_sp_ of 60 F g^−1^ at 1 A g^−1^. Furthermore, the SSC device displayed the cycling stability of 99% after 10 000 GCD cycles (Fig. 33b). An excellent energy density of 23 W h kg^−1^ at a power density of 600 W kg^−1^ was found (Fig. 33b). The electrode material displayed pseudocapacitive behaviour with the involvement of six electrons and six protons during the reversible redox-processes (Fig. 33b). The present study gives in-depth information on a newer MOF design and its application in long-term stable SC devices.

Some of the most intensively investigated organic materials for pseudocapacitor applications are perylene diimde (PDI)-based small molecules, polymers, porous polymers and MOFs. As shown in Table 5, among the reported small molecule structure-based materials, the PPAC/PDI-1 electrode in three-electrode SCs delivered the highest *C*_sp_ of 617 F g^−1^ at 0.5 A g^−1^.^152^ In addition, PPAC/PDI-1 in a two-electrode setup exhibited the highest *C*_sp_ of 310 F g^−1^ (specific capacity: 69 mA h g^−1^) and maximum energy density of 62.3 W h kg^−1^ at a power density of 455 W kg^−1^.^152^ Similar results were found for the PDI-PDA electrode in aqueous and gel elctrolytes.^157^ In addition, polymers and porous polymers derived from PDI demonstrated a significant improvement in the electrochemical properties of PSCs. Although the PHATN^165^ electrode displayed the highest *C*_sp_ of 363 F g^−1^ (110 mA h g^−1^) at 1 A g^−1^ (two-electrode small pouch cell), its energy density was found to be average of 16.1 W h kg^−1^ at 145 W kg^−1^ power density. In this case, the BT-PDI^174^ electrode in an SSC device displayed an excellent energy density of 52.9 W h kg^−1^ at a power density of 2.9 kW kg^−1^. With the long-term objective of fabricating PDI-based SCs, the Ni-MOF-24 h electrode was prepared, which also displayed an excellent energy density of 23 W h kg^−1^.^178^ Thus, the incorporation of PDI in polymers, porous polymers and MOFs significantly improved the specific capacitance, energy density and power density. Therefore, the utilization of PDIs is an attractive scaffold to improve the charge-storage capacity of polymers and MOFs. The major contribution of the specific capacitance of these electrode materials appeared from faradaic reversible redox-processes.

**Table 5:** Comparison of the electrochemical properties of perylene diimide (PDI)-based small molecules, polymers, covalent organic frameworks (COFs), and metal organic frameworks (MOFs)

Compound code	Electrolyte	Type of working electrode	Specific capacitance (*C*_sp_)	Energy density (ED)	Power density (PD)	Ref.
**PDI-based small molecules**						
F-127 templated CNFs	2 M H_2_SO_4_	Three-electrode	226 F g^−1^ at 4 A g^−1^	13 W h kg^−1^	Approximately ∼200 W kg^−1^, see Fig. 6	150
PANI-H_2_SO_4_-PDITCA-50	1 M H_2_SO_4_	Two-electrode	460 F g^−1^ at 0.3 A g^−1^	23 W h kg^−1^	200 W kg^−1^	151
PPAC/PDI-1	1 M H_2_SO_4_	Three-electrode	617 F g^−1^ at 0.5 A g^−1^	—	—	152
		Two-electrode ASC	310 F g^−1^ (specific capacity: 69 mA h g^−1^)	62.3 W h kg^−1^	455 W kg^−1^	
PDI-Py/GF	1 M H_2_SO_4_	Two-electrode SSC	197 F g^−1^ at 1 A g^−1^	46 W h kg^−1^ at	3060 W kg^−1^	153
PDI-Pyr/GF	1 M H_2_SO_4_	Two-electrode SSC	192 F g^−1^ at 1 A g^−1^	54 W h kg^−1^	2700 W kg^−1^	154
Ti_3_C_2_T_*x*_@*c*PDI	1 M ZnCl_2_	Three-electrode	Sp. capacity 67 mA h g^−1^ at 5 mV s^−1^	—	—	155
	1 M MnCl_2_		Sp. capacity 51 mA h g^−1^ at 5 mV s^−1^			
	1 M CaCl_2_		Sp. capacity 75 mA h g^−1^ at 5 mV s^−1^			
ITO/PDI-AB	PMMALiClO_4_–acetonitrile–PC gel	Two-electrode SSC	33.87 ± 0.66 mF g^−1^ at 0.5 mA g^−1^	12.04 ± 0.23 mW h kg^−1^	1.6 ± 0.03 W kg^−1^	156
		Flexible SC	32.68 ± 0.44 mF g^−1^	11.62 ± 0.15 mW h kg^−1^	1.6 ± 0.02 W kg^−1^	
PDI-PDA	1 M H_2_SO_4_	Three electrode	610 F g^−1^ at 1 A g^−1^	—	—	157
	Gel electrolyte prepared by dissolving carboxymethyl cellulose (1.5 g) and Na_2_SO_4_ (2 g) in water	Two-electrode ASC	310 F g^−1^ at 1 A g^−1^	Approximately ∼150 W h kg^−1^ see Fig. 5e	Approximately ∼9000 W kg^−1^ see Fig. 5e	
GS/Q1 (cell-3)	1 M H_3_PO_4_	Two-electrode SSC	146.54 F g^−1^ (*via* impedance analysis at 10 mHz) and 118.33 F g^−1^ (*via* CV at 5 mV s^−1^)	—	—	158
PDI-NDI-PDI	1 M H_2_SO_4_	Two-electrode SSC	193.33 F g^−1^ at 0.5 A g^−1^	34.80 W h kg^−1^	1079.98 W kg^−1^	159

**PDI based polymers and porous polymers**						
Porous-1/Ni	1 M Na_2_SO_4_	Three electrode	352 F g^−1^ at 0.2 A g^−1^ specific capacity 59 mA h g^−1^	—	—	164
Porous-2/Ni			238 F g^−1^ at 0.2 A g^−1^			
PHATN	6 M KOH	Three-electrode	689 F g^−1^ at 0.5 A g^−1^	—	—	165
		Two-electrode small pouch cell	363 F g^−1^ (110 mA h g^−1^) at 1 A g^−1^	16.1 W h kg^−1^	145 W kg^−1^	
PEDOT/PDI polymer film exhibits	0.1 M TBAPF_6_	Two electrode SSC	78.6 F g^−1^ (positive region) and 73.1 F g^−1^ (negative region) at 0.5 A g^−1^	8.95 W h kg^−1^	76.8 kW kg^−1^	166
P(PDI-*alt*-BDT)	1 M PC-LiClO_4_	Single-electrode setup	113 F g^−1^ at 0.5 A g^−1^	9.1 W h kg^−1^	82 kW kg^−1^	168
P(PDI2OD-T2)/MWCNT	1 M PMMA LiClO_4_	Two-electrode SSC	85.4 F g^−1^ at 0.25 A g^−1^			169
		Flexible quasi-solid-state device	84.2 F g^−1^ at 0.25 A g^−1^	112.4 W h kg^−1^	18 600 W kg^−1^	
Py-Ph-Pery	1.0 M KOH	Symmetric coin cell configuration	84 F g^−1^	23.33 W h kg^−1^	—	171
TPE-Ph-Pery			26 F g^−1^	7.32 W h kg^−1^	—	
TPA-Ph-Pery			23 F g^−1^	6.49 W h kg^−1^	—	
BT-PDI	0.1 M TBAPF_6_	Three electrode	196 F g^−1^ at 1 A g^−1^	—	—	174
		Two-electrode SSC	42.33 F g^−1^ at 1 A g^−1^	52.9 W h kg^−1^	2.9 kW kg^−1^	
NH-PDI	0.5 M H_2_SO_4_	Three electrode	363 F g^−1^ at 0.5 A g^−1^			176
	PVA + H_2_SO_4_ (1 : 1)	Two-electrode SSC	134.2 F g^−1^ at 0.5 A g^−1^	22.5 W kg^−1^ at 0.5 A g^−1^	274.8 W kg^−1^ at 0.5 A g^−1^.	

**PDI-based MOFs**						
Ni-MOF-24 h	1 M H_2_SO_4_	Three electrode	198 F g^−1^ at 1 A g^−1^	—	—	178
		Two electrode SSC	60 F g^−1^ at 1 A g^−1^	23 W h kg^−1^	600 W kg^−1^	

### 4.3. Naphthalene diimide (NDI) supercapacitors

**Fig. 34:**
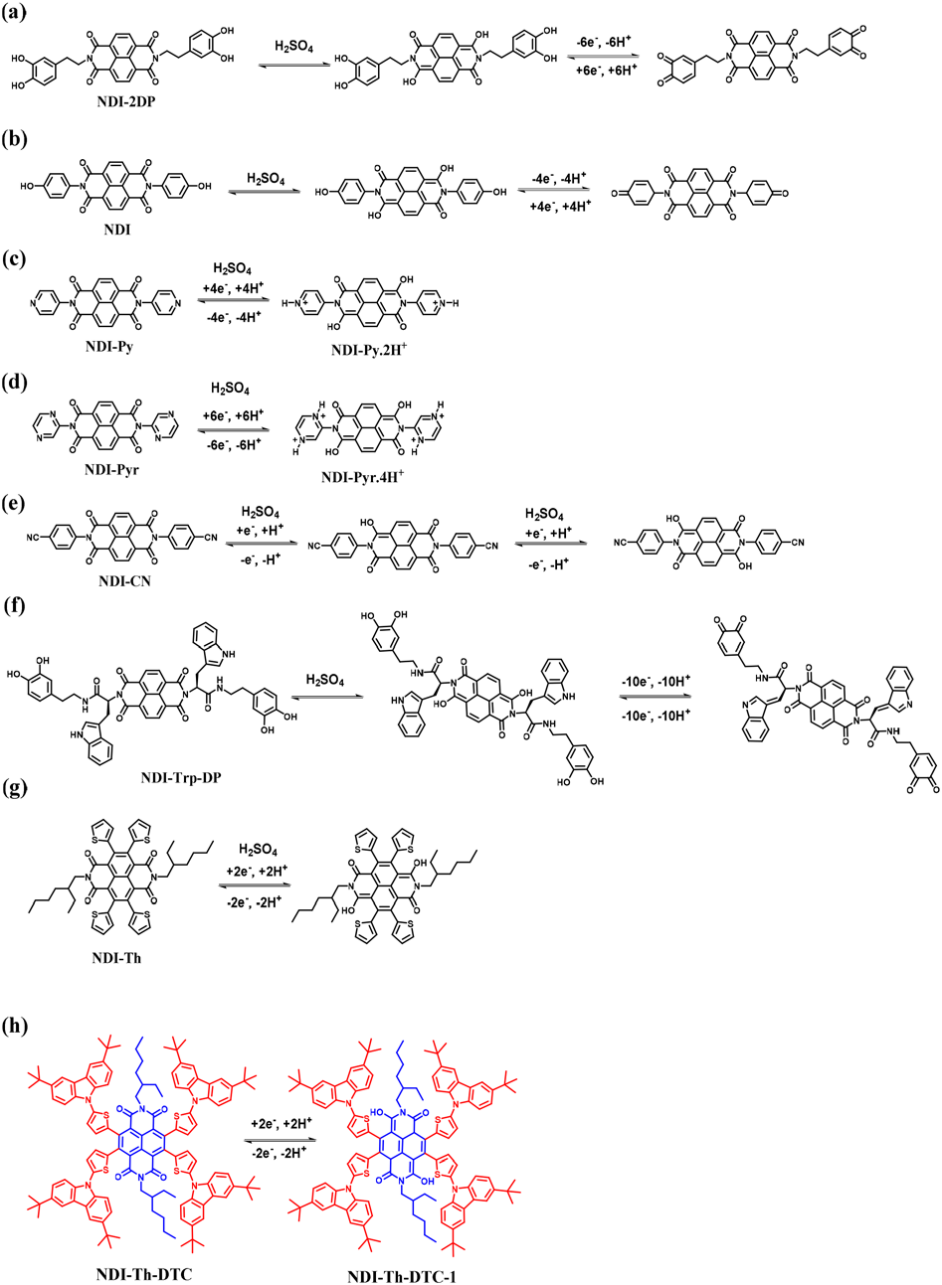
Molecular structure of redox-active naphthalene diimide derivatives: (a) NDI-2DP, (b) NDI, (c) NDI-Py, (d) NDI-Pyr, (e) NDI-CN, (f) NDI-Trp-DP, (g) NDI-Th and (h) NDI-Th-DTC and their plausible redox-properties for pseudocapacitor applications.

Naphthalene diimide (NDI) is a smaller rylene diimide (RD) homologue and exhibits high electron-acceptor properties, good charge carrier characteristics and physical, chemical and thermal stability.^179^ Moreover, NDI and its derivatives display reversible redox-reactions. The imide and core functionalization of NDI makes it an attractive candidate for sensing and optoelectronic applications.^180–182^ Owing to these properties, NDIs (Fig. 34) have been recently utilized in energy storage applications, particularly in pseudocapacitor^183^ and batteries^147^ and pseudocapacitors. Thus, to tackle challenges such as solubility in the electrolyte solution and reversible side reactions posed by organic electrode materials, Bhosale and co-workers reported the synthesis of an organic redox-active material, *i.e.* NDI-2DP (Fig. 34a),^184^ derived from the redox-active NDI and dopamine^185^ subunits. The as-fabricated NDI-2DP/carbon paper (CP) electrode in a three-electrode system showed an impressive *C*_sp_ of 195.9 F g^−1^ at 0.5 A g^−1^ in the potential window of 0 to 1 V, which is higher compared to that of the mono-dopamine NDI derivative (NDI-1DP) (137.2 F g^−1^). In a three-electrode SC configuration, NDI-2DP displayed excellent cycling stability with 96% retention of its initial *C*_sp_ value after 10 000 GCD cycles. Notably, it was found that the solid-state symmetric supercapacitor device with the NDI-2DP/CF electrode exhibited 83.1 F g^−1^ at 5 mV s^−1^ and 73.1 F g^−1^ at 0.5 A g^−1^. The organic supercapacitor device based on the NDI-2DP/CP composite electrode also displayed an energy density as high as 10.1 W h kg^−1^ at 0.49 kW kg^−1^. The redox-reaction of NDI-2DP involved six electrons and six protons and showed pseudocapacitive behaviour (Fig. 34a). The present investigation implies that organic electrode materials derived from NDI with redox-active functionalization can pave a new way to design and develop SC cell configurations with the manipulation of the NDI structure at the imide positions. The redo-behaviour of the organic molecular architecture can be manipulated using Coulomb interaction, which can be achieved by adding electron-withdrawing/donating subunits and functionalizing the organic scaffolds. This type of modification will help widen the applied potential voltage window.^186^ The tuning of the polarity of organic molecules can be helpful for the modification of their physical and chemical characteristics. This modification is helpful for efficiently utilizing the active sites in organic electrode materials. In addition, an enhancement in the π-conjugation of the core organic subunits can be utilized to enhance the surface redox-reactions. Hu and co-workers demonstrated the imide-functionalization of naphthalene dianhydride (NDA) with *p*-aminophenol to yield naphthalene diimide (NDI) (Fig. 34b) with extended conjugation.^187^ Further, they fabricated the NDI/rGO organic electrode material (OEM) *via* the non-covalent functionalization of rGO. In the three-electrode SC configuration, the NDI/rGO electrode exhibited the *C*_sp_ of 354 F g^−1^ and 433 F g^−1^ at 5 mV s^−1^ and 1 A g^−1^, respectively. Also, it showed 87.2% specific capacitance retention after 8000 cycles at 3 A g^−1^. Further, they reported the fabrication of the GH-DN//rGO-NDI ASC device using GH-DN 1 : 2, and rGO-NDI 1 : 2 as the positive and negative electrode, respectively, in 1 M H_2_SO_4_ electrolyte. The ASC device showed a *C*_sp_ as high as 111.3 F g^−1^ at 5 mV s^−1^. The GCD profile of the ASC exhibited the maximum energy density of 26.3 W h kg^−1^ at a power density of 0.6 kW kg^−1^. NDI involves four electrons and four protons during the reversible redox-reaction and exhibits pseudocapacitive behaviour (Fig. 34b). It is important to note that NDI-Py (Fig. 34c) and NDI-Pyr (Fig. 34d), lower homologues of rylene diimde, displayed an inferior electrochemical performance compared to their higher homologues PDI-Py (Fig. 25c)^153^ and PDI-Pyr (Fig. 25d),^154^ respectively. NDI-Py and NDI-Pyr undergo reversible redox reactions during the electrochemical process involving 4e^−^/4H^+^ (Fig. 34c) and 6e^−^/6H^+^ (Fig. 34d), respectively. Deshmukh *et al.* reported the preparation of newer electrode materials based on a benzonitrile-appended NDI organic electrode material denoted as NDI-CN (Fig. 34e).^188^ The presence of an electron-withdrawing subunit can help build a system with multi-electronic redox reactions. In addition, the additional aromatic units may help increase the π-conjugation. This is beneficial for achieving active OEMs with an enhanced operational voltage window and higher energy density. Using the NDI-CN molecular scaffold and reduced graphene oxide (rGO), they fabricated the rGO/NDI-CN composite electrode material. At a current density of 0.5 A g^−1^, the SC device based on the rGO/NDI-CN composite electrode displayed a *C*_sp_ as high as 336 F g^−1^. The reversible redox-reaction of NDI-CN displayed pseudocapacitive behaviour and the involvement of two electrons and two protons during the reversible electrochemical process (Fig. 34e). The rGO/NDI-CN OEM showed long cycling stability of 80% specific capacitance retention after 10 000 GCD cycles at 10 A g^−1^. The higher performance of the rGO/NDI-CN composite electrode could be attributed to the enhanced charge transport characteristics of rGO due to the strong π–π interaction between the NDI-CN subunit with higher conjugation and rGO structure. To enhance the operation potential voltage window, Bhosale and co-workers reported the design and synthesis of an acceptor–donor–acceptor–donor–acceptor (A–D–A–D–A) molecular skeleton using NDA, tryptophan and dopamine, denoted as NDI-Trp-DP (Fig. 34f).^189^ The NDI-Trp-DP active-organic material was used in combination with graphite foil to fabricate the NDI-Trp-DP/GF electrode material. The applied potential window was established as 0 to 1.0 V. The pseudocapacitive NDI-Trp-DP/GF in 1 M H_2_SO_4_ electrolyte in a three-electrode SC displayed the *C*_sp_ of 323 F g^−1^ at 0.5 A g^−1^ and 152 F g^−1^ in the NDI-Trp-DP/GF//NDI-Trp-DP/GF SSC device at the same current density. The SSC device delivered an energy density of 19 W h kg^−1^ at a power density of 900 W kg^−1^. Moreover, the device displayed the excellent *C*_sp_ retention of 97.76% after 10 000 GCD cycles. The performance of the device could be ascribed to the reversible faradaic redox-reactions involving ten electrons and ten protons (Fig. 34f) in the electrochemical process exhibited by NDI-Trp-DP and synergistic influence shown by CP with their higher conductivity. Therefore, the organic electrode materials derived from rich renewable resources may be helpful to reduce the cost of electrode materials, and in turn the cost of PSC cell configurations. This investigation will be helpful for the design of newer electrode materials for real-world applications of the SCs. The core-substituted NDI molecular architecture is also an important analogue for SC applications. Very recently, thiophene-functionalized NDI (NDI-Th) (Fig. 34g) was synthesized and employed in SC applications.^190^ The NDI-Th/GF-based SSC device delivered a *C*_sp_ of 77.76 F g^−1^ at 0.5 A g^−1^ and cycle life of 84.83% *C*_sp_ after 10 000 GCD cycles with an energy density of about 11.66 W h kg^−1^ at a power density of 899.92 W kg^−1^. The reversible faradaic process involves two electrons and two protons (Fig. 34g) during the electrochemical process. These cost-effective D–A organic redox-active materials can contribute to pseudocapacitive advancement for the new generation of SCs. In continuation of this, Bhosale and co-workers developed a new organic material, 4,5,9,10-tetrakis(5-(3,6-di-*tert*-butyl-9*H*-carbazol-9-yl)thiophen-2-yl)-2,7-bis(2-ethylhexyl)benzo[lmn][3,8]phenanthroline-1,3,6,8(2*H*,7*H*)-tetraone (NDI-Th-DTC), to investigate the influence of a strong electron-donor group on the charge-storage properties of the naphthalene diimide core. The as-fabricated electrode based on NDI-Th-DTC/GF in a three-electrode supercapacitor cell configuration at a current density of 0.5 A g^−1^, displayed a *C*_sp_ of 128.03 F g^−1^. In addition, they examined the practical application of the electrode using a two-electrode NDI-Th-DTC/GF//NDI-Th-DTC/GF symmetric supercapacitor (SSC) device. The SSC delivered the *C*_sp_ of 86.03 F g^−1^ at 0.5 A g^−1^. It is noticeable that the SSC device also exhibited an energy density as high as 12.90 W h kg^−1^ at a power density of 899.65 W kg^−1^. At a current density of 2 A g^−1^, NDI-Th-DTC/GF//NDI-Th-DTC/GF displayed cycling stability with the capacity retention of 77.67% and 99.71% coulombic efficiency after 4000 GCD cycles.

**Fig. 35:**
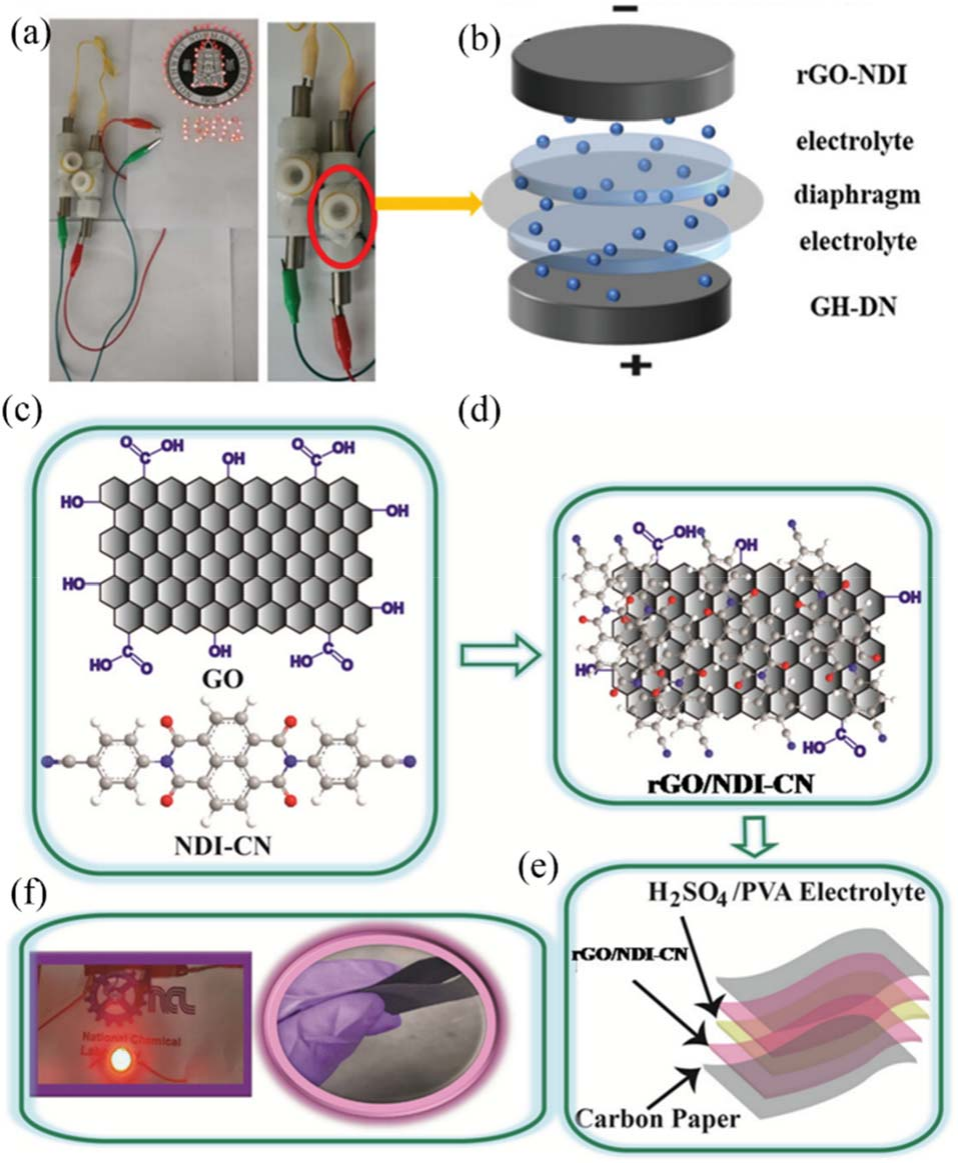
(a) Photographic image of glowing 81 LED lamps powered by (b) as-assembled GH-DN//rGO-NDI ASC component. Reproduced from ref. 187 with permission from [John Wiley and Sons], Copyright [2021]. (c) NDI-CN and GO used for the preparation of (d) rGO-NDI-CN composite electrode materials, (e) schematic presentation of flexible device and (f) picture of LED light illumination. Reproduced from ref. 188 with permission from [Elsevier], Copyright [2022].

To demonstrate the real-world practical applicability of NDI-based small organic molecules as active-electrode materials, the as-fabricated rGO-NDI electrode (Fig. 34b) based on two GH-DN//rGO-NDI ASC devices in series was utilized by Hu and co-workers to illuminate 81 LED lamps.^187^ After charging, the LED lamp was continuously lit for 13 s (Fig. 35a). The ASC device constructed from a positive electrode, negative electrode, electrolyte and diaphragm, as shown in Fig. 35b. Similarly, two SC flexible solid-state devices based on rGO/NDI-CN derived from the organic-redox moiety (Fig. 34e) in series were utilized for glowing an LED lamp (Fig. 35b).^188^ These practical applications indicate that organic molecule-based composite electrodes are novel candidates as next-generation green energy storage materials with potential applications in portable devices.

#### 4.3.1. NDI polymer supercapacitors

**Fig. 36:**
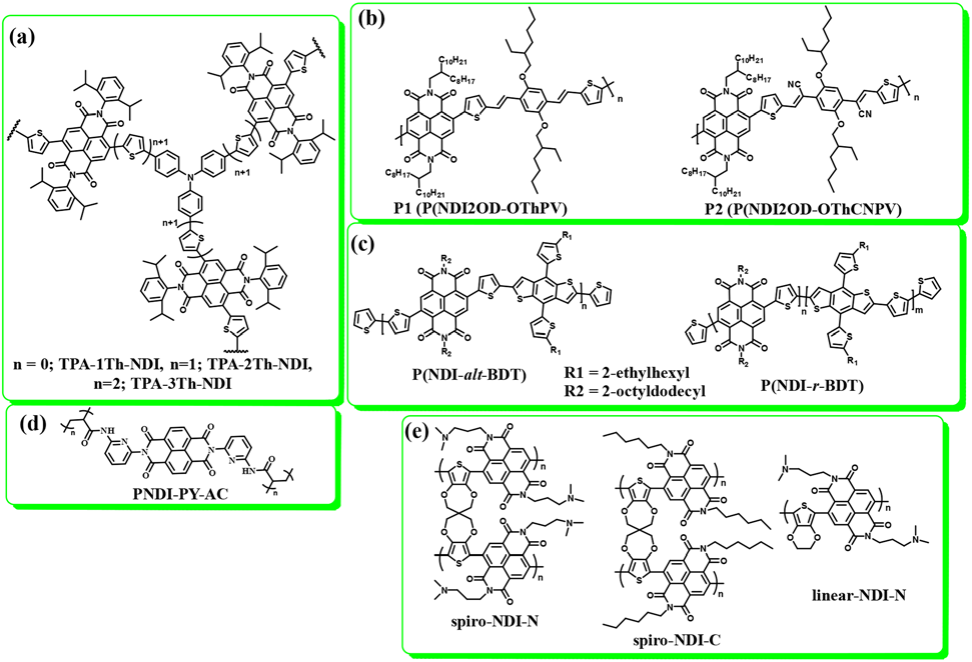
Molecular structure of various polymers (a) TPA-1Th-NDI, TPA-2Th-NDI, TPA-3Th-NDI, (b) P1 (P(NDI2OD-OThPV)), P2 (P(NDI2OD-OThCNPV)), (c) P(NDI-*alt*-BDT), P(NDI-*r*-BDT), (d) PNDI-Py-AC and (e) spiro-NDI-N, spiro-NDI-C, linear-NDI-N based on naphthalene diimide (NDI).

Among the pseudocapacitor materials, linear polymers, which display π–π-stacking interactions, are used in SC applications. These polymers show good π–π-stacking and exhibit high conductivity. However, although the conductivity shown by these polymers is high they also hinder electrolyte diffusion, which can limit the charge-storage properties. Thus, compared to activated carbon, liner polymer-based SCs display limited charge-storage capacities.^191^ Thus, to overcome these limitations, Luscombe and co-workers developed n-type hyperbranched polymers named TPA-1Th-NDI, TPA-2Th-NDI and TPA-3Th-NDI based on naphthalene diimde (NDI) and triphenylamine (TPA) subunits.^192^ Subsequently, asymmetric supercapacitors based on the TPA-1Th-NDI, TPA-2Th-NDI and TPA-3Th-NDI polymers (Fig. 36) as the cathode and activated carbon as the anode were fabricated, which displayed the *C*_sp_ of 22.0, 4.92, and 4.94 F g^−1^, respectively. The ASC cell configuration exhibited excellent stability after 500 GCD cycles. Further, the SSC device based on the TPA-1Th-NDI polymer showed a *C*_sp_ as low as ∼5 F g^−1^, together with excellent stability over 500 GCD cycles with only 10% loss of its charge-storage capacity. The present work demonstrates the design and use of synthetic chemistry to obtain polymeric materials with a controlled structure and successful utilization in type III polymeric SCs. Asha and co-workers demonstrated the synthesis and charge-storage capacity of two polymers named P1 (P(NDI2OD-OThPV)) and P2 (P(NDI2OD-OThCNPV)) (Fig. 36b).^193^ The P1 and P2 polymers exhibited intramolecular charge transfer (ICT) properties according to their absorption spectra. The P1 and P2 polymer-based symmetric polymer composite supercapacitors in the applied voltage window of −0.7 to 0.5 V displayed the *C*_sp_ of 84 and 124 F g^−1^. The SC device showed 100% specific capacitance retention after 5000 cycles. Further, in 0.5 M H_2_SO_4_ as the electrolyte, the performance of P2 was examined in a full cell device configuration, which delivered a capacitance of 40 F g^−1^ at 0.5 A g^−1^. The present work investigated by the Asha group is a step forward for the fabrication of type III SC devices using donor–acceptor polymers in the EES field. The same group demonstrated the use of D–A polymers, *e.g.* P(NDI-*alt*-BDT) and P(NDI-*r*-BDT) (Fig. 36c), based on NDI subunits.^168^ The charge storage results obtained from the P(NDI-*alt*-BDT) and P(NDI-*r*-BDT) (Fig. 36c) polymers are inferior compared to P(PDI-*alt*-BDT) (Fig. 30a) and P(PDI-*r*-BDT) (Fig. 30b).^168^ Biradar *et al.* reported the synthesis of the three-dimensional PNDI-PY-AC polymer based on the redox-active pyridine-functionalized NDI.^194^ The BET analysis was performed using nitrogen adsorption–desorption isotherms to estimate the SSA and pore size distribution of the PNDI-PY-AC polymer electrode material. The as-fabricated PNDI-PY-AC composite electrode displayed a type-IV isotherm. The evaluated SSA and pore diameter were found to be 12.73 m^2^ g^−1^ and 7.99 nm, respectively, indicating the mesoporous nature of the material with a pore volume of 0.0498 cm^3^ g^−1^. This material is suitable for enhanced charge-storage applications. The SSC device based on the PNDI-PY-AC/GF polymer (Fig. 36d) exhibited a *C*_sp_ of 202.85 F g^−1^ at a current density of 0.5 A g^−1^. It is important to note that the SSC configuration at 0.5 A g^−1^ showed an excellent energy density of 49.69 W h kg^−1^ at a power density of 1259.99 W kg^−1^. After 5000 GCD cycles, the SSC device showed 92.86% *C*_sp_ retention at the current density of 2 A g^−1^. Thus, the cross-linked polymer with two electron withdrawing groups exhibited a pseudocapacitive performance, suggesting the importance of this architecture in EES applications. Very recently, Bazan and co-workers reported the preparation of new cross-linked n-type conjugated spiro-NDI-N, spiro-NDI-C and linear-NDI-N polymers (Fig. 36e).^195^ The BET analysis of the three polymers based on N^2^ adsorption isotherms at 77 K suggested that they are mesoporous (2–50 nm) structures. The spiro-NDI-N and spiro-NDI-C cross-linked polymers displayed an SSA of 80 and 76 m^2^ g^−1^, respectively, which are higher than that of linear-NDI-N (40 m^2^ g^−1^). This can facilitate rapid ion-electronic coupling, resulting in higher pseudocapacitive charge storage. The spiro-NDI-N polymer with polar tertiary amine side chains in aqueous pH neutral electrolyte exhibited the *C*_sp_ of 532 F g^−1^ at 5 A g^−1^. They found that spiro-NDI-N (532 F g^−1^) exhibited a superior rate capability compared to linear-NDI-N (198 F g^−1^) and spiro-NDI-C (104 F g^−1^) at 5 A g^−1^. In 2 M NaCl aqueous electrolyte solution, the lower specific capacitance exerted by spiro-NDI-C could be attributed to its hydrophobic side chains. The presence of hydrophobic chains results in poor interfacial contact with the aqueous electrolyte solution, which lowers the ionic conductivity in the polymer network. Also, spiro-NDI-N displayed 83% *C*_sp_ retention after 5000 GCD cycles at a current density of 100 A g^−1^, which is slightly higher than that of linear-NDI-N (75% *C*_sp_ retention). Moreover, at a current density of 350 A g^−1^, spiro-NDI-N maintained a rate capability of 307 F g^−1^.

**Fig. 37:**
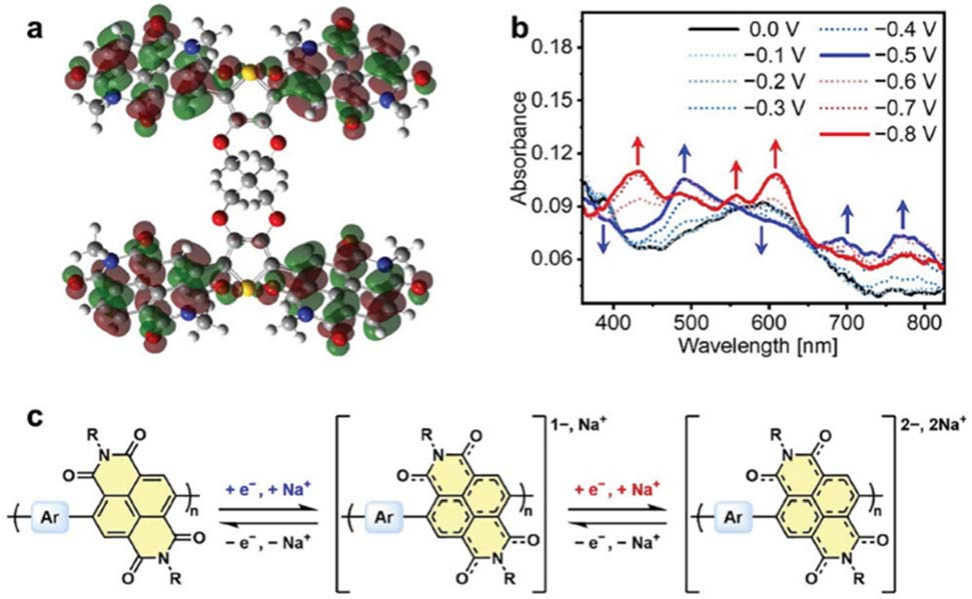
Spiro-NDI-N-based pseudocapacitive energy storage mechanism. (a) LUMO of spiro-NDI-N; (b) spectroelectrochemical measurements of spiro-NDI-N film in aqueous 2 M NaCl electrolyte at an applied potential in the range of 0.0 V to −0.8 V; and (c) proposed pseudocapacitive charge storage mechanism of polymers based on NDIs. Reproduced from ref. 195 with permission from [John Wiley and Sons], Copyright [2024].

Further, the charge storage mechanism of spiro-NDI-N (Fig. 36e)^195^ was examined by means of DFT (Fig. 37). The calculation of the lower unoccupied molecular orbital (LUMO) energy level distribution using the B3LYP/6-31G(d,p) level of theory was found to be delocalized over the NDI subunits (Fig. 37a). This implies that the charge-storage is predominantly exhibited by the NDI moiety, which was revealed by *in situ* spectroelectrochemistry experiments (Fig. 37b). As shown in Fig. 37c, the NDI subunits present in the polymer showed a reversible one-electron reduction process to yield electron-polaron of NDI and further electron-bipolaron processes.^196^ The absorption profile of an NDI film on ITO-coated glass was examined by sweeping at the applied potential in the range of 0 to −0.8 V. Dual band absorption peaks were observed at 345 and 620 nm (Fig. 37b, black line) at the applied potential of 0 V. With a decrease in the applied potential from 0 to −0.5 V, the absorption peaks at 345 and 620 nm were found to decrease, and at the same time new peaks appeared at 490, 693 and 773 nm (Fig. 37b, blue line), indicating the formation of the electron-polaron singly reduced state of the NDI subunit.^197^ It is noticeable that a reduction in the applied voltage from 0 to −0.8 V resulted in formation of new peaks at 430, 557 and 609 nm (Fig. 26b, red line), which could be attributed to the formation of electrons-bipolarons due to the second reduced state of NDI.^197^ According these results, the charge-storage mechanism was established, which can be ascribed to the participation of a two-electron reversible redox-reaction process. The cross-linked polymer derived from the redox-active small NDI molecules displayed high charge storage capabilities, which could be ascribed to the larger electroactive surface area accessible by electrolyte ions and enhancement of the ion transportation and diffusion within the polymer network. The present investigation offers a general strategy for the development of organic pseudocapacitors with higher performance.

#### 4.3.2. NDI-based COF supercapacitors

**Fig. 38:**
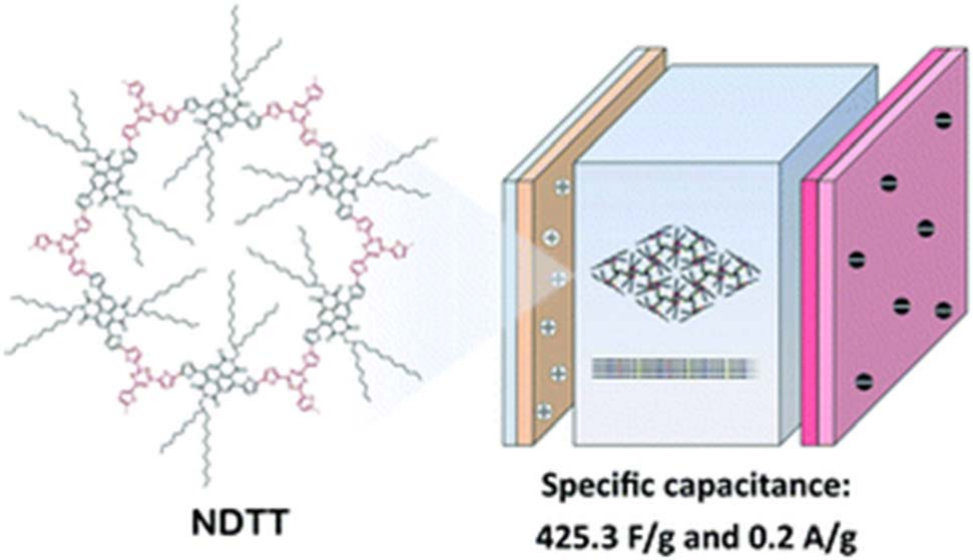
Molecular structure of COF and schematic illustration of pseudocapacitor device architecture. Reproduced from ref. 200 with permission from RSC.

In recent years, the fabrication of covalent organic frameworks (COFs) using redox-active organic building blocks has attracted attention from researchers due to their higher surface areas, abundant pores and low densities.^198^ COFs have shown importance in the fields of optoelectronics, gas storage and separation, drug delivery and energy storage.^199^ Owing to their importance, in 2022, Tan and co-workers developed a COF-like conjugated framework denoted as NDTT using NDI and 2,4,6-tri(thiophen-2-yl)-1,3,5-triazine (3TT) bearing strong-electron withdrawing 1,3,5-triazine groups *via* a C–C single bond linkage (Fig. 38).^200^ The BET surface area of the NDTT COF was observed to be 32.5 m^2^ g^−1^. Its lower in SSA compared to traditional COFs could be attributed to the presence of long alkyl chains in its structure. This was also observed in the COF bearing long glycol chains. The electrochemical performance of NDTT with nickel foam as the conducting material in the three-electrode SC configuration was examined in 1 M KOH. The GCD measurements in the operational potential window of 0.3 V to 0.45 V displayed the *C*_sp_ of 425.3 F g^−1^ at 0.2 A g^−1^ with an excellent energy density of 33.2 W h kg^−1^. The excellent electrochemical characteristics of NDTT COF are ascribed to the reversible faradaic reactions of its conjugated system bearing abundant redox-active sites within the COF. The present GDTT results are competitive compared to the literature reports based on COFs without doping.

This research work paves the way to design and synthesize new COF-like redox-active materials for the construction of SCs with higher performances.

#### 4.3.3. NDI-based metal organic framework (MOF) supercapacitors

**Fig. 39:**
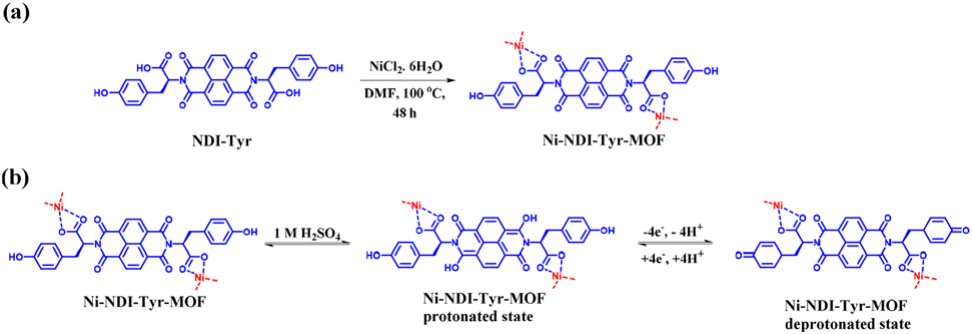
(a) Method for the preparation and (b) redox-mechanism of Ni-Tyr-NDI-MOF.

MOFs have emerged as a new family of electrode materials for SC applications.^201^ The performance of MOFs is impressive compared to traditional materials. However, their low potential window, limited cycling stability and poor rate performance are the challenges that need to be addressed for the development of develop MOF-based SCs for practical applications. Therefore, there is a need for develop newer organic ligands that can enhance the charge storage performance of MOFs. In this regard, Li and co-workers reported three novel MOFs based on the NDI ligand in combination with nickel, calcium and magnesium metal ions named [Ni(H_2_L1)(DMF)] complex 1, [Ca(H_2_L2)(DMF)] complex 2 and [Mg(H_2_L2)] complex 3.^202^ Complex 1 and complex 2 were composed of 2D polymers and complex 3 exhibited a 3D coordination network. The charge-storage performance of the electrodes based on these complexes were investigated in 0.5 M TADPF_6_ electrolyte. In the three-electrode cell configuration, the calculated *C*_sp_ of complex 1, and complex 2 and complex 3 in the operational potential window of −2.5 to 1.0 V was 214 F g^−1^, 141 F g^−1^, and 127 F g^−1^ at 1 A g^−1^, respectively. Among them, complex 1 showed the highest performance, retaining 80% of its initial *C*_sp_ value after 6000 GCD cycles. Also, an SSC device, complex 1 at 0.5 A g^−1^ exhibited the highest *C*_sp_ of 102 F g^−1^ and excellent specific energy density of 28 W h kg^−1^ among the complexes. It is notable that at a current density of 8 A g^−1^, the SSC device based on complex 1 displayed 98% specific capacitance retention after 5000 cycles, suggesting the importance of MOFs in SC applications. Inspired by the use of MOFs in pseudocapacitive energy storage applications, Bhosale and co-workers developed newer Ni-Tyr-NDI-MOF redox-active materials (Fig. 39) based on n-type conducting tyrosine-functionalized NDI in combination with nickel metal ions. The Ni-Tyr-NDI-MOF (Fig. 39a)-based Ni-Tyr-NDI-MOF/GF electrode was utilized in three- and two-electrode supercapacitor device configurations.^203^ The nitrogen adsorption–desorption isotherm-based BET analysis of Ni-Tyr-NDI-MOF showed that it possessed a surface area of 11.23 m^2^ g^−1^ and pore volume of 29.98 nm. These BET parameters play a crucial role in enhancing the charge-storage characteristics of the Ni-Tyr-NDI-MOF electrode materials. In the three-electrode device architecture, the Ni-Tyr-NDI-MOF/GF electrode in aqueous 1 M H_2_SO_4_ electrolyte in the potential window of 0.0 to 1.4 V (*vs.* Ag/AgCl) at 5 mV s^−1^ and 1 A g^−1^ showed the *C*_sp_ of 294.20 F g^−1^ and 330.71 F g^−1^, respectively. In contrast, the Ni-Tyr-NDI-MOF/GF//Ni-Tyr-NDI-MOF/GF SSC cell in the applied potential window of 0 to 1.4 V at 0.5 A g^−1^ exhibited the calculated *C*_sp_ of 180 F g^−1^. Moreover, the SSC device exhibited an excellent energy density of 44.1 W h kg^−1^ at 0.5 A g^−1^ with the power density of 1265.02 W kg^−1^. The SSC device displayed 82.3% specific capacitance retention and 91.97% coulombic efficiency after 10 000 GCD cycles, implying the impressive performance of the MOF-based SSC device. The pseudocapacitive performance of the Ni-Tyr-NDI-MOF/GF electrode is ascribed to the reversible faradaic-redox reactions in 1 M H_2_SO_4_ electrolyte exhibited by the NDI-Tyr organic ligand (Fig. 39b). Herein, the authors presumed that the faradaic process involves 4e^−^/H^+^ during the reversible reactions. Thus, the Ni-Tyr-NDI-MOF materials derived from renewable resources are attractive scaffolds and useful for designing newer materials for next-generation PSC applications.

Similar to PDIs, NDI-based small molecule-, polymer-, COF- and MOF-based electrode materials were examined for their electrochemical properties and the results are summarized in Table 6. Among the tested NDIs for SC applications, NDI/rGO^187^ displayed the highest *C*_sp_ of 433 F g^−1^ at 1 A g^−1^ and excellent energy density of 26.3 W h kg^−1^. Among the reported polymers, PNDI-PY-AC/GF^194^ derived from NDI showed an excellent *C*_sp_ and energy density of 202.85 F g^−1^ at 0.5 A g^−1^ and 49.69 W h kg^−1^, respectively. The NDTT^200^ COF displayed the highest *C*_sp_ of 425.3 F g^−1^ at 0.2 A g^−1^ and maximum energy density of about 33.2 W h kg^−1^. In addition, our literature search revealed only one Ni-Tyr-NDI-MOF^203^ based on NDI, which displayed an excellent specific capacitance 180 F g^−1^ at 0.5 A g^−1^ as well as the outstanding energy density of 44.1 W h kg^−1^ at a power density of 1265.02 W kg^−1^. Thus, NDIs have been successfully utilized to prepare polymers, COFs and MOFs. These materials possess a porous morphology, increased specific area and electronic conductivity, leading to enhanced electrochemical properties. Subsequently, the energy density is significantly improved. Therefore, NDI-based materials are attractive alternatives to traditional electrode materials for the fabrication of next-generation SCs.

**Table 6:** Comparison of the electrochemical properties of naphthalene diimide (NDI)-based small molecules, polymers, covalent organic frameworks (COFs), and metal organic frameworks (MOFs)

Compound code	Electrolyte	Type of working electrode	Specific capacitance (*C*_sp_)	Energy density (ED)	Power density (PD)	Ref.
**NDI-based small molecules**						
NDI-2DP/CP	1 M H_2_SO_4_	Three electrode	195.9 F g^−1^ at 0.5 A g^−1^	—	—	184
		Two-electrode solid-state SSC	73.1 F g^−1^ at 0.5 A g^−1^	10.1 W h kg^1^	0.49 kW kg^1^	
NDI/rGO	1 M H_2_SO_4_	Three electrode	354 F g^−1^ at 5 mV s^−1^ and 433 F g^−1^ at 1 A g^−1^	—	—	187
		Two-electrode ASC	111.3 F g^−1^ at 5 mV s^−1^	26.3 W h kg^−1^	0.66 kW kg^−1^	
rGO/NDI-CN	1 M H_2_SO_4_	Three electrode	336 F g^−1^ at 0.5 A g^−1^	16.8 W h Kg^−1^ at 0.5 A g^−1^	149.6 W kg^−1^ at 0.5 A g^−1^	188
	(PVA)/H_2_SO_4_	Two-electrode flexible SSC	53 mF cm^−2^ at 0.5 mA cm^−2^	9.54 μW h cm^−2^	0.3 mW cm^−2^	
NDI-Trp-DP/GF	1 M H_2_SO_4_	Three electrode	323 F g^−1^ at 0.5 A g^−1^	—	—	189
		Two-electrode SSC	152 F g^−1^ at 0.5 A g^−1^	19 W h kg^−1^	900 W kg^−1^	
NDI-Th/GF	1 M H_2_SO_4_	Three electrode	173.33 F g^−1^ at 0.5 A g^−1^	—	—	190a
		Two-electrode SSC	77.76 F g^−1^ at 0.5 A g^−1^	11.66 W h kg^−1^	899.92 W kg^−1^	
NDI-Th-DTC	1 M H_2_SO_4_	Three electrode	128.03 F g^−1^ at 0.5 A g^−1^	—	—	190b
		Two-electrode SSC	86.03 F g^−1^ at 0.5 A g^−1^	12.90 W h kg^−1^	899.65 W kg^−1^	

**NDI based polymers**						
TPA-1Th-NDI	1 M TEATFB in 1 : 1 propylene carbonate: dimethyl carbonate	Two-electrode ASC	22.0 F g^−1^	—	—	192
TPA-2Th-NDI			4.92 F g^−1^	—	—	
TPA-3Th-NDI			4.94 F g^−1^	—	—	
P1 (P(NDI2OD-OThPV))	0.5 M H_2_SO_4_	Two-electrode SSC	84 F g^−1^ at 0.5 A g^−1^	—	—	193
P2 (P(NDI2OD-OThCNPV))			124 F g^−1^ at 0.5 A g^−1^	2 W h kg^−1^	22 kW kg^−1^	
PNDI-PY-AC/GF	1 M H_2_SO_4_	Three electrode	440.41 F g^−1^ at 0.5 A g^−1^	—	—	194
		Two-electrode SSC	202.85 F g^−1^ at 0.5 A g^−1^	49.69 W h kg^−1^	1259.99 W kg^−1^	
Spiro-NDI-N	2 M NaCl	Three-electrode	532 F g^−1^ at 5 A g^−1^	—	—	195
Linear-NDI-N			198 F g^−1^ at 5 A g^−1^	—	—	
Spiro-NDI-C			104 F g^−1^ at 5 A g^−1^	—	—	

**Naphthalene diimide based COFs**						
NDTT/nickel foam	1 M KOH	Three-electrode	425.3 F g^−1^ at 0.2 A g^−1^	33.2 W h kg^−1^	—	200

**NDI based MOFs**						
[Ni(H_2_L1)(DMF)] complex 1	0.5 M TBAPF_6_	Three-electrode	214 F g^−1^ at 1 A g^−1^	—	—	202
[Ca(H_2_L2)(DMF)] complex 2			141 F g^−1^ at 1 A g^−1^	—	—	
[Mg(H_2_L2)] complex 3			127 F g^−1^ at 1 A g^−1^	—	—	

**NDI MOF**						
Ni-Tyr-NDI-MOF	1 M H_2_SO_4_	Three-electrode	330.71 F g^−1^ at 0.5 A g^−1^	—	—	203
		Two-electrode SSC	180 F g^−1^ at 0.5 A g^−1^	44.1 W h kg^−1^ at 0.5 A g^−1^	1265.02 W kg^−1^ at 0.5 A g^−1^	

### 4.4. Heterocyclic-functionalized arylimide-based COFs

**Fig. 40:**
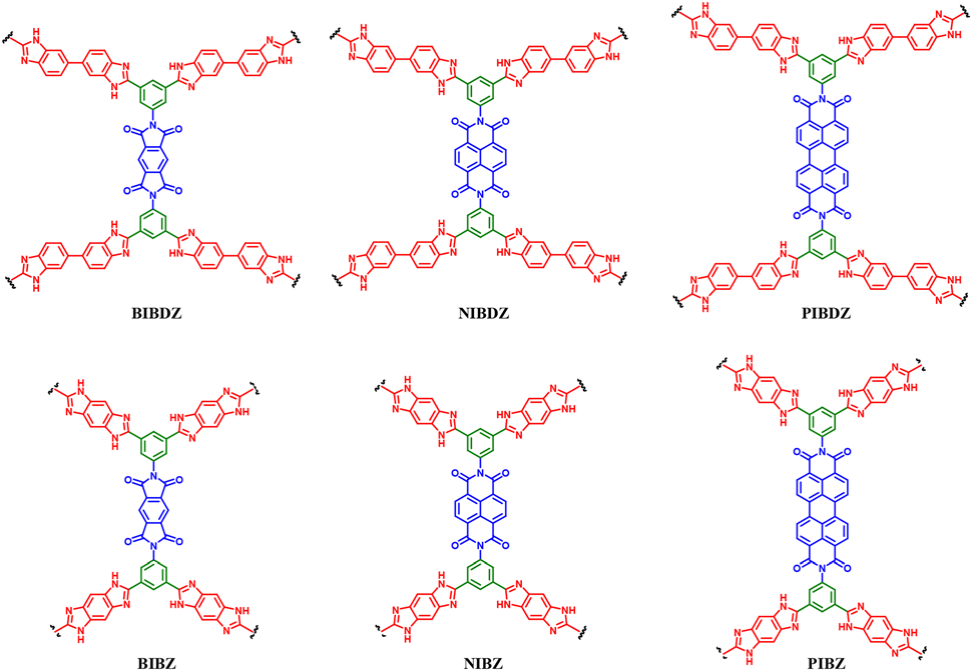
Porous cross-linked polymer structures of benzimidazole-linked arylimide constituted from 3,3′-diaminobenzedene (DAB) and (b) 1,2,4,5-benzenetetraamine (TAB).

Malik and co-workers demonstrated the preparation and application of heterocyclic compound benzimidazole-appended arylimide such as (pyromellitic, naphthalene and perylene)-based COFs such as BIBDZ, NIBDZ, PIBDZ, BIBZ, NIBZ and PIBZ (Fig. 40) *via* a condensation polymerization protocol.^204^ Among the synthesized COFs, BIBDZ displayed the highest BET surface area of 177.095 m^2^ g^−1^ with pore a diameter of 30–32 Å. The BIBDZ material in 1 M H_3_PO_4_ electrolytic solution showed a *C*_sp_ of 88.4 F g^−1^ at a current density of 0.5 A g^−1^. The device also exhibits 93.61% specific capacitance retention over 5000 GCD cycles. This investigation offers a new path to design and synthesize heterocyclic compound-appended π-conjugated materials for supercapacitor applications.

Thus, NDI-based small organic molecules, polymers, COFs and MOFs will be extensively utilized in SC applications.

### 4.5. Aza-based organic materials for SCs

**Fig. 41:**
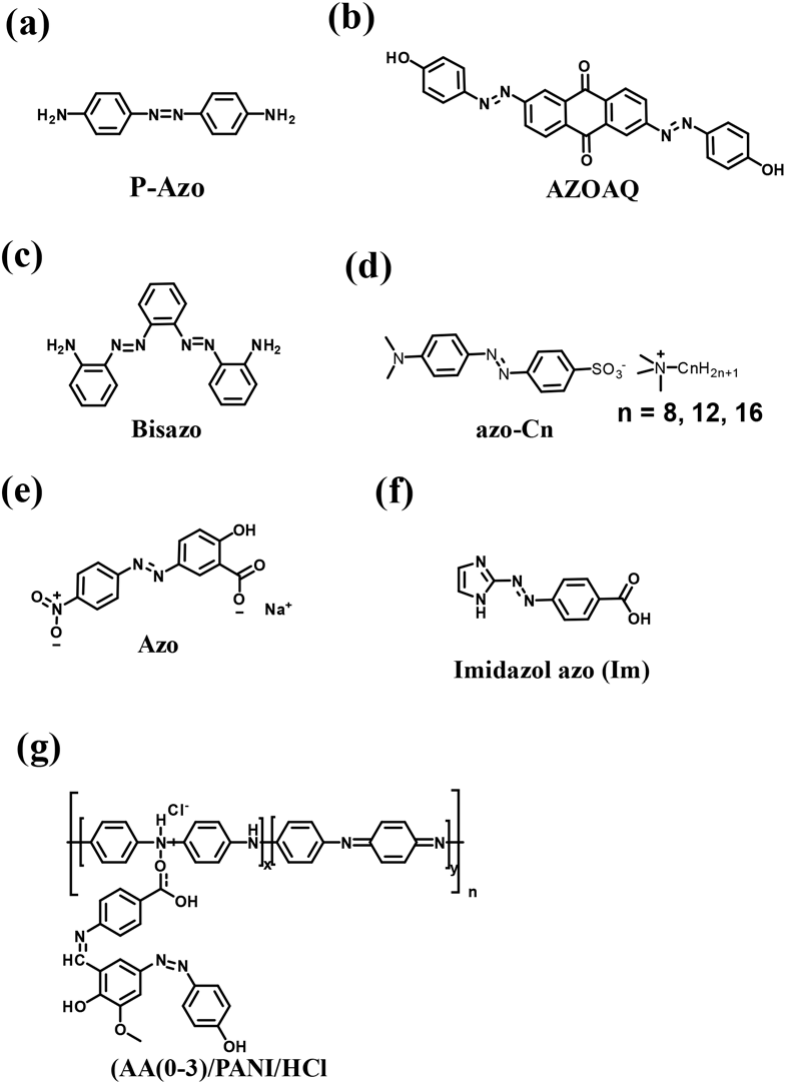
Structures of azo derivatives (a) P-Azo, (b) AZOAQ, (c) Bisazo, (d) azo-Cn, (e) AZO, (f) Imidazol azo (Im), (g) (AA (0-3)/PANI/HCl used for supercapacitor applications.

**Fig. 42:**
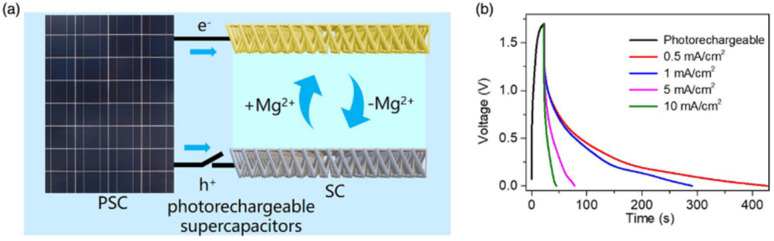
(a) Working principle of photo-rechargeable supercapacitors and (b) GCD profiles of photo-rechargeable supercapacitors. Reproduced from ref. 212 with permission from [John Wiley and Sons], Copyright [2023].

Organic electrode materials are extracted from abundant renewable sources^205^ at a low cost with simple processability and higher theoretical specific capacity,^206^ which can display higher rate kinetics due to their fast ion-transportation and diffusion.^207^ These OEMs are used for the fabrication of flexible supercapacitor cell configurations. Redox-active organic compounds bearing sulfide (S–S), carbonyl (C=O), and imine (C=N) functional groups as well as organic radicals (–O) are mainly employed for SC applications.^208^ However, the higher solubility of compounds bearing S–S small bonds in the electrolyte solution during the redox process limits their electrochemical properties.^209^ Although 2,2′,6,6′-tetramethylpiperidine-1-oxyl (TEMPO)-bearing organic radicals display higher conductivity and electrochemical rate, they exhibit fewer active sites, resulting in a lower energy density.^210^ Therefore, in recent years, organic compounds bearing C=N and C=O functional groups have appeared as important scaffolds to achieve higher charge-storage and release processes. In addition to this class of compounds, azo-based organic electrode materials with N=N functional groups have been reported to displayed high charge storage capacity *via* reversible redox processes.^206,208^ Organic compounds with N=N functional groups showed a lower LUMO energy level, resulting into faster electron transfer, and ultimately enhancing the conductivity of the active electrode materials.^208,211^ Moreover, N≡N in organic molecules (Fig. 41) undergoes two-electron transfer during redox-processes, which is helpful to enhance the energy density of SC devices. Considering all these facts, Liu and co-workers fabricated an asymmetric flexible SC device using P-azo (Fig. 41a) and activated carbon as electrodes.^212^ At a current density of 0.1 mA cm^−2^, the areal specific capacity was calculated to be 1195.88 mF cm^−2^ for P-Azo/MgCl_2_/AC FSC, which is higher than that for the P-Azo/ZnCl_2_/AC FSC flexible ASC device of 76.27 mF cm^−2^. Moreover, the P-Azo/MgCl_2_/AC FSC displayed a higher energy density of 425.2 mW h cm^−2^ (55.19 W h kg^−1^) at a power density of 80 mW cm^−2^ (10.38 W kg^−1^).^212^ The results for the flexible ASC device are impressive compared to the values reported in the literature for similar systems. P-Azo/MgCl_2_/AC FSC in its bending state showed 90.7% capacitance retention of its initial value over 80 000 cycles. These authors further demonstrated the application of the P-Azo/MgCl_2_/AC FSC to prepare photo-rechargeable supercapacitors (Fig. 42).^212^ The cathode and anode of the SC were connected to the cathode and anode of the PSC (Fig. 42a). At an intensity of 100 mW cm^−2^, the charging curve of the SC device was examined, followed by turning off the light-source to get the discharge profiles in the dark at various current densities (Fig. 42). The GCD measurements of the FSC at 0.5 mA cm^−2^ (Fig. 42b) showed energy storage of 47.77 mW h cm^−2^. At a higher current density, the charge and discharge times were relatively close (Fig. 42b). The photo-rechargeable SC at a current density of 1 mA cm^−2^ showed the conversion efficiency of 7%, which is competitive compared to the similar SCs reported in the literature thus far.^213^ Thus, the present investigation on the fabrication of a flexible SC configuration provides a new path to design and develop practical future wearable devices. Bhosale and co-workers reported the synthesis of an azo-functionalized anthraquinone derivative named 2,6-bis((*E*)-(4-hydroxyphenyl)diazenyl)anthracene-9,10-dione (AZOAQ) (Fig. 41b) and their utilization in three-electrode SC and two-electrode SSC devices.^214^ The AZOAQ/GF//AZOAQ/GF SSC configuration at 0.5 A g^−1^ displayed *C*_sp_ of 159.12 F g^−1^ with the excellent energy density of 28.64 W h kg^−1^ at a power density of 1080.02 W kg^−1^. The SSC device showed excellent cycling stability of 93.22% over 10 000 GCD cycles. These cheaper organic scaffolds appear as excellent materials for pseudocapacitor applications and provides the basis for designing new materials based on the N=N functional group.

Zhao and co-workers fabricated electrode materials based on 2,2′-diaminobis(*o*-azobenzene) (bisazo) (Fig. 41c) and reduced graphene oxide (rGO).^215^ The bisazo compound was anchored on rGO *via* covalent bonds to yield bisazo-rGO-3. The three-electrode SC device displayed a *C*_sp_ of 581 F g^−1^ at a current density of 1 A g^−1^ with an energy density as high as 24.5 W h kg^−1^ at a power density of 27 kW kg^−1^. The two N=N functional groups present in bisazo showed reversible four-electron transfer during the electrochemical process and displayed pseudocapacitive behaviour in combination with the EDLC mechanism. The best-performing cycling stability and its coin cell application makes bisazo-rGO-3 an appealing material for future real-world applications in EES technologies. In 2020, Chang *et al.* reported the preparation of self-assembled azobenzene complexes with surfactant denoted as azo-C_*n*_, where *n* = 8, 12 and 16 (Fig. 41d) for pseudocapacitor applications.^216^ The as-fabricated azo-C_*n*_ at 10 mV s^−1^ displayed a *C*_sp_ of 221.0 F g^−1^ through a reversible redox process. Azo-C8 showed the maximum *C*_sp_ of 204.5 F g^−1^ at a current density of 5.0 A g^−1^. In the case of the azo-C8-based electrodes, the estimated energy density was found to be 13.9 W h kg^−1^ at a power density of 1.5 kW kg^−1^ and maintained 4.4 W h kg^−1^ at 7.8 kW kg^−1^. Upon going from *n* = 8 to 16, the *C*_sp_ exhibited by the SC device gradually decreased. The performance of the SC based on azo-C_*n*_ (*n* = 8) was higher, which could be attributed to its better hydrophilic surface. Thus, these materials are useful for the fabrication of cost-effective and high-efficiency SC devices. Balakrishnan and co-workers reported the synthesis of the PANI/GO-Azo electrode material based on PANI-grafted graphene oxide (GO)-azopyridine (Azo) (Fig. 41e). The N=N functional group in the molecular structure acted as a linker between PANI and GO.^217^ The surface area of the as-prepared PANI/GO-Azo electrode material was determined using N_2_ adsorption–desorption isotherm. According to the BET analysis of the isotherms, the SSA of PANI/GO-Azo was 30.33 m^2^ g^−1^, which was higher compared to the PANI (13.33 m^2^ g^−1^) and PANI-GO (14.44 m^2^ g^−1^) electrode materials reported in the literature. The BJH analysis displayed the mesoporous configuration of PANI/GO-Azo with a pore size in the range of 2 to 20 nm. This large mesoporous size distribution provides the basis for faster ion transportation, resulting in electrical double layer characteristics. In addition, it can help enhance the interaction between the redox-active materials and electrolyte for better charge-storage properties in the SC device. In aqueous 1 M H_2_SO_4_ electrolyte, the PANI/GO-Azo nanocomposite electrode at a current density of 1 A g^−1^ displayed the *C*_sp_ of 426 F g^−1^. The SC device showed excellent cycling stability of 98.5% over 5000 GCD cycles. Furthermore, the PANI/GO-Azo//AC ASC device displayed the *C*_sp_ of 296.36 F g^−1^ at 0.5 A g^−1^. In contrast, the device at 0.5 A g^−1^ showed an areal capacitance of 592.7 mF cm^−2^. The PANI/GO-Azo//AC ASC device was successfully utilized to light a red LED light at a working voltage of 1.7 V. Chen *et al.* developed a new electrode material based on the carbonization of a 5-[(4-nitrophenyl)azo]salicylate-zinc complex from sodium 5-[(4-nitrophenyl)azo]salicylate (Fig. 41e) by mixing with zinc ions.^218^ The BET analysis of electrode materials displayed SSA and a total pore volume of 1177.2 m^2^ g^−1^ and 0.89 cm^3^ g^−1^, respectively. The higher surface area could help to enhance the electrochemical properties of the as-fabricated electrode material. The three-electrode SC device based on the as-fabricated electrode showed the *C*_sp_ of 266.2 F g^−1^ at 1 A g^−1^. In contrast, the two-electrode cell configuration showed the excellent energy density of 33.4 W h kg^−1^ at a power density of 0.5 kW kg^−1^. These electrode materials could be prepared using different metal ions and azo-bearing organic compounds. Conducting materials such as carbon nanotube (CNT) functionalization with organic active material brings new dimensions to electrode materials for SC applications. Balakrishnan and co-workers functionalized carbon nanotube-grafted polypyrrole (PPy) using imidazole azo (I_m_) (Fig. 41f) as the organic moiety.^219^ The electrochemical performance of the conductive CNT-based Im-CNT/PPy electrode was enhanced with the anchoring of I_m_. The as-fabricated electrode in 1 M H_2_SO_4_ electrolyte at a current density of 1 A g^−1^ showed the *C*_sp_ of 305 F g^−1^. The higher electrochemical performance is ascribed to the π–π stacking interactions between PPy and I_m_-CNT. The I_m_-CNT/PPy composite electrode material provided a higher surface area and stability during the electrochemical process.

To alter the electrical and optical properties of the polymer main chain, the azo-azomethine moiety can be incorporated.^220^ The azo-azomethine scaffolds bearing donor and acceptor subunits within the molecular structure displayed intramolecular charge transfer (ICT) properties. Aziz and co-workers demonstrated the doping of PANI using the azo-azomethine chromophore and its charge-storage properties upon varying the concentration of the chromophore.^221^ The as-prepared AA3/PANI/HCl (Fig. 41g) showed the *C*_sp_ of 816.9 F g^−1^, which is higher compared to the undoped pure polymer, exhibiting the *C*_sp_ value of 161.17 F g^−1^ at the scanning rate of 50 mV s^−1^. Thus, owing to the enhanced electrochemical conductivity of this doped polymer, it displays promise for SC applications.

#### 4.5.1. AZO polymer for supercapacitor applications

**Fig. 43:**
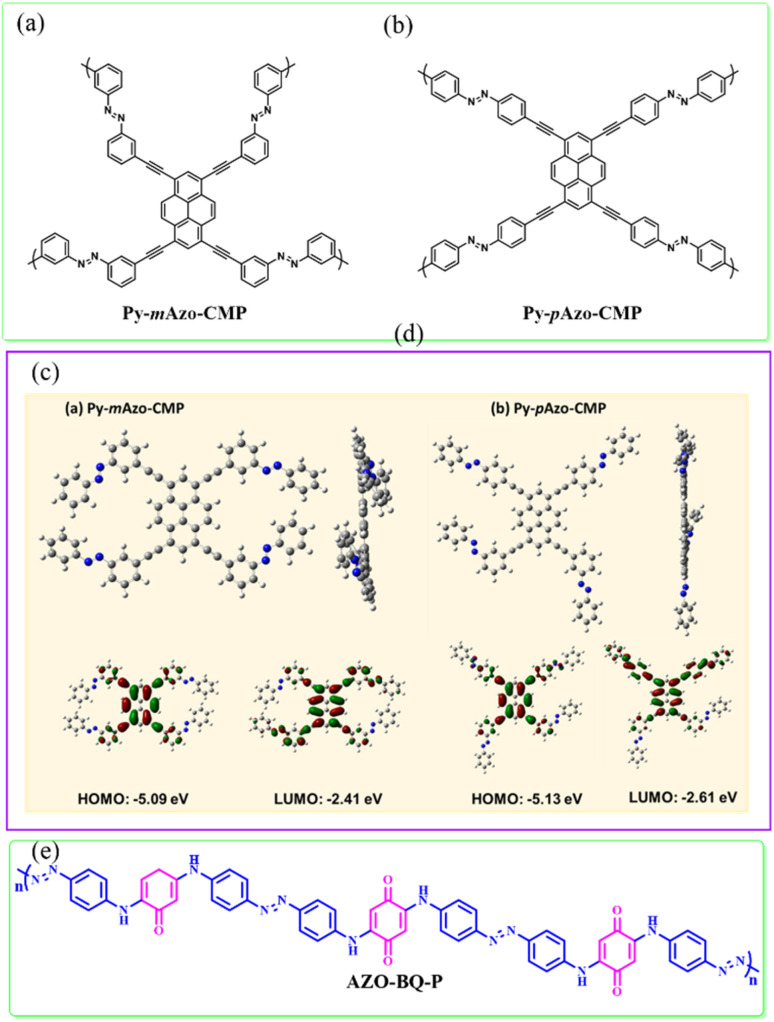
Structures of (a) Py-*m*Azo-CMP and (b) Py-*p*Azo-CMP, and frontier molecular orbitals of (c) Py-*m*Azo-CMP and (d) Py-*p*Azo-CMP. Reproduced from ref. 227 with permission from [the American Chemical Society], Copyright [2023]. (e) Structure of AZO-BQ-P polymer.

To address the escalating energy storage challenges, the their pore sizes and effective surface area of porous organic polymers (POPs) bearing high porosity^222^ can be manipulated, making them attractive candidates for the preparation of electrodes.^223^ The conjugated microporous polymers (CMPs) in the POP category offer various benefits such as microporous structure and π-conjugation network structures.^224^ These CMPs are utilized in energy storage applications due to their capacity to incorporate different redox-active components, potential to have 2D and 3D structures, diffusion of electrolyte ions, enlarged surface area and faster kinetic behaviour, enhancing the charge-storage properties.^225^ To examine CMPs bearing a two-electron redox-active azo (N=N) functional group and charge-storage characteristics,^226^ Kuo and co-workers demonstrated the synthesis and charge-storage properties of two CMPs (Fig. 43) named Py-*m*Azo-CMP (Fig. 43a) and Py-*p*Azo-CMP (Fig. 43b).^227^ The nitrogen adsorption and desorption studies were performed at 77 K to estimate the SSA, total pore volume and pore size distribution of the as-synthesized Py-*m*Azo and Py-*p*Azo-CMPs. The BET analysis of the isotherms demonstrated that the SSA and total pore volume of Py-*m*Azo-CMP and Py-*p*Azo-CMP are 176 m^2^ g^−1^ and 0.36 cm^3^ g^−1^ and 279 m^2^ g^−1^ and 0.39 cm^3^ g^−1^, respectively. The pore size distribution for Py-*m*Azo-CMP and Py-*p*Azo-CMP was centred at 8.094 and 5.66 nm, respectively. The hysteresis profile of the isotherms indicates the mesoporous framework of the Py-*m*Azo and Py-*p*Azo-CMPs structures. In accordance with the IUPAC classification, the Py-*m*Azo and Py-*p*Azo-CMP electrode material displayed type-I and type-IV isotherms, respectively, implying the presence of porous structures. The BET analysis indicated that Py-*m*Azo and Py-*p*Azo-CMPs are attractive materials for enhanced charge-storage applications. The redox-active azo group is located at the *meta*- and *para*-locations in Py-*m*Azo-CMP and Py-*p*Azo-CMP, respectively. Their main aim was to study the impact of azo substituents at different positions. The charge-storage properties of Py-*m*Azo-CMP and Py-*p*Azo-CMP were tested in a 1 M KOH electrolyte solution. At a current density of 1 A g^−1^, Py-*p*Azo-CMPs displayed the *C*_sp_ of 142 F g^−1^, which is higher than that of Py-*m*Azo (93 F g^−1^).^227^ Both the Py-*m*Azo and Py-*p*Azo-CMP electrodes showed excellent cycling stability, retaining 94.63% and 93% of their *C*_sp_, respectively, after 5000 GCD cycles at 10 A g^−1^. Moreover, Py-*p*Azo-CMP and Py-*m*Azo-CMP displayed an energy density as high as 19.72 W h kg^−1^ and 12.92 W h kg^−1^, respectively.^227^ The DFT calculations using the Gaussian 09W software at the B3LYP/6-31G(d) level were carried out to establish the electronic properties of Py-Azo-CMPs and their ability to store charge. The D3BJ dispersion correction was considered to have long-range and non-covalent interactions. The frontier molecular orbitals of Py-*m*Azo and Py-*p*Azo-CMPs are shown in Fig. 43c and d, respectively.^227^ It was observed that the LUMO energy level of the Py-*m*Azo and Py-*p*Azo-CMPs showed significant localization over their conjugated backbones. It was noticed that Py-*p*Azo-CMP displayed improved LUMO energy level delocalization, resulting in a lower energy band gap, which can be an important feature to exhibit an enhanced charge-storage performance. The present investigation highlights the effect of the presence and position of the redox-active azo functional group in the construction of porous Py-Azo-CMPs, displaying significant electrical energy storage (EES) characteristics. Very recently, Jagadale *et al.* reported the synthesis and pseudocapacitive properties of an azo-functionalized benzoquinone (AZO-BQ-P) polymer (Fig. 43e).^228^ The BET analysis based on the nitrogen adsorption–desorption isotherms of the AZO-BQ-P polymer displayed a type-III isotherm, indicating high adsorption capability. The BET parameters such as SSA and pore volume estimated from the isotherm were observed to be 14.94 m^2^ g^−1^ and 0.1474 cm^3^ g^−1^, respectively. These results demonstrate that the AZO-BQ-P polymer is suitable for charge-storage applications by enhancing the electrode/electrolyte contact. The AZO-BQ-P/GF composite electrode was prepared using active polymeric materials and graphite foil. The AZO-BQ-P/GF//AZO-BQ-P/GF SSC device displayed the *C*_sp_ of 200 F g^−1^ at 0.5 A g^−1^ with the energy density of 25.00 W h kg^−1^ at a power density 900.00 W kg^−1^. The SSC device showed the cycling stability of 86.18% retention of its *C*_sp_ initial value after 5000 cycles. The present polymer concept can be utilized for the construction of efficient SC devices.

#### 4.5.2. Aza COF supercapacitors

**Fig. 44:**
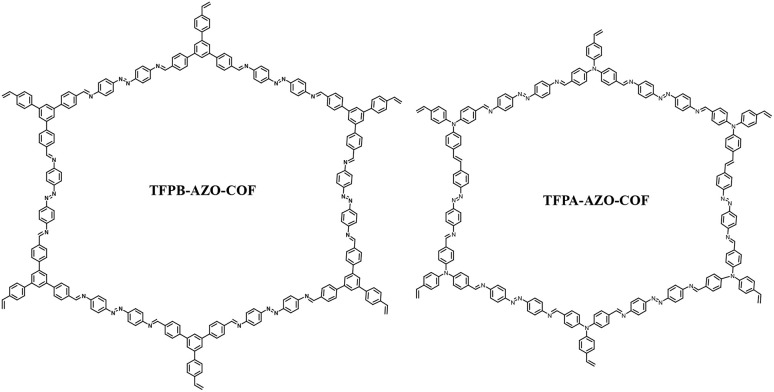
Chemical structures of TFPB-AZO-COF and TFPA-AZO-COF.

COFs are attractive candidates for electrochemical charge storage due to their nanoscale porosity, significant redox activity and charge transfer capability. It is important to note that the redox-active building blocks of COFs enhance the reversible faradaic process, suggesting that these materials are remarkable candidates for the fabrication of EES devices. In COFs, the electrode–electrolyte contact enhances the ion diffusion through their pores and the faster kinetics can be attributed to their π-conjugated system.^229^ Asadinezhad and co-workers successfully synthesized two COFs, *i.e.* COF (TFPA-AZO-COF) from azodianiline (AZO) and tris(4-formyl phenyl) amine (TFPA) and TFPB-AZO-COF from AZO and 1,3,5-tris(*p*-formyl phenyl) benzene (TFPB) (Fig. 44).^230^ The nitrogen adsorption–desorption analysis was performed to estimate the SSA, pore volume and pore diameter of the as-prepared TFPB-AZO-COF and TFPA-AZO-COF electrode materials. In the case of TFPB-AZO-COF, its total SSA, average pore diameter and pore volume distribution were 987 m^2^g^−1^, 1.65 nm and 0.8 cm^3^g^−1^, whereas that for TFPA-AZO-COF was 425 m^2^g^−1^, 1.73 nm and 1.34 cm^3^g^−1^, respectively. The BST parameters indicate that the as-prepared TFPB-AZO-COF and TFPA-AZO-COF displayed a crystalline topology with larger surface area and higher porosity. These parameters make these materials ideal for ion transportation during the charge/discharge processes. The Barrett–Joyner–Halenda (BJH) diagrams of TFPB-AZO-COF and TFPA-AZO-COF indicate that their pores are in the microscale range. Thus, the BJH analysis demonstrated that the COFs exhibited macroporous structures. The larger SSA and pore volume of these COFs make them potential organic electrode materials for SC applications. Their charge-storage properties were examined in a three-electrode system using alkaline electrolyte solution. At a current density of 1 A g^−1^, the TFPB-AZO-COF and TFPA-AZO-COF-based three-electrode SC devices exhibited the specific capacity of 450 F g^−1^ and 160 F g^−1^, respectively. The two-electrode ASC device displayed the *C*_sp_ of 61.4 F g^−1^ and 24.47 F g^−1^ for the TFPB-AZO-COF and TFPA-AZO-COF, respectively, at a current density of 1.0 A g^−1^. Moreover, at 10 A g^−1^, the TFPB-AZO-COF and TFPA-AZO-COF ASC devices exhibited the energy densities of 24.6 W h kg^−1^ and 9.8 W h kg^−1^ at the power densities of 8.5 kW kg^−1^ and 8.6 kW kg^−1^, respectively. The enhanced redox activity of TFPB-AZO-COF and TFPA-AZO-COF could be ascribed to the presence of the azo (–N=N–) subunit and abundant covalent conjugated double bonds. Moreover, the higher specific capacitance of these COFs was possible due to their larger surface area. However, the synthesis of these complex electrode materials on an industrial-scale is not cost-effective. Therefore, more research is needed for the development of protocols that can be scaled-up.

#### 4.5.3. AZO MOFs for supercapacitors

**Fig. 45:**
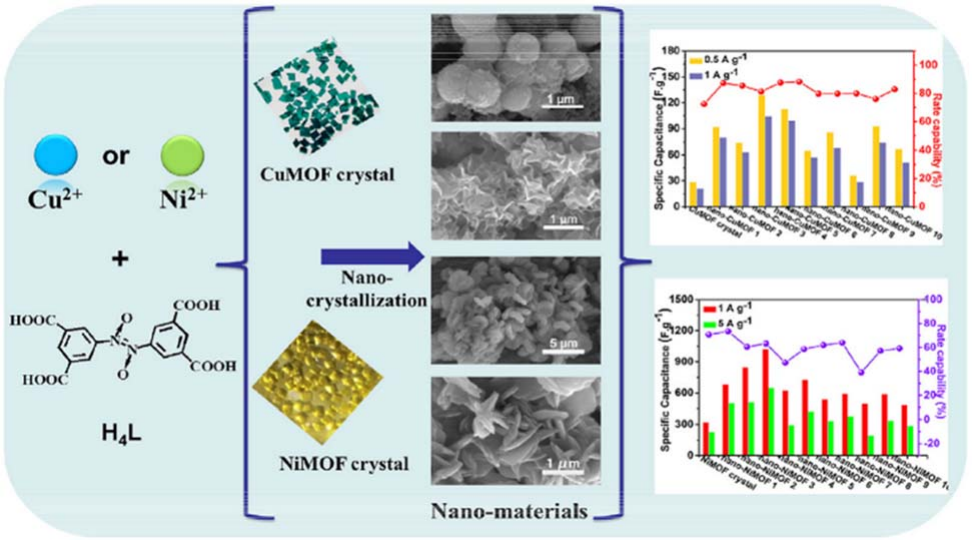
Molecular structure of 3,5-dicarboxyl-(3′,5′-dicarboxylazophenyl)benzene acid H_4_L ligand, SEM images of MOF nanomaterials and specific capacitance of the CuMOF crystal and NiMOF crystal. Reproduced from ref. 232 with permission from [Elsevier], Copyright [2020].

In recent years, metal–organic frameworks (MOFs) have emerged as promising electrode materials for supercapacitor applications. To enhance the charge-storage properties of MOFs, it is necessary to reduce their particle size. MOFs with a particle size on the nanometer scale help to reduce the distance between the electrode and electrolyte.^231^ Yi and co-workers prepared MOFs such as CuMOF and NiMOF using Cu(NO_3_)_2_·3H_2_O and NiCl_2_·4H_2_O, respectively, and 3,5-dicarboxyl-(3′,5′-dicarboxylazophenyl)benzene acid (H_4_L) ligand (Fig. 45).^232^

Further, to achieve higher charge-storage properties, they utilized the *in situ* solvothermal method to prepare nano-CuMOF 1–10 and nano-NiMOF 1–10. The size of nano-CuMOF and nano-NiMOF was controlled by means of solvent and surfactant effects. They tested the electrochemical properties of the MOFs ranging from CuMOF and NiMOF to nano-CuMOF and nano-NiMOF as electrode materials. Among the examined electrode materials based on these MOFs, nano-NiMOF-3 displayed the best *C*_sp_ of 1024 F g^−1^ at a current density of 1 A g^−1^ and maintained a very good cycling life (Fig. 45). The ASC device based on nano-NiMOF-3 and activated carbon exhibited *C*_sp_ as high as 38.65 F g^−1^ at 0.5 A g^−1^.^232^ Moreover, the nano-NiMOF3//AC ASC displayed an energy density as high as 13.74 W h kg^−1^ at a power density of 400 W kg^−1^, and 8.5 W h kg^−1^ at a power density of 2400 W kg^−1^. It is important to notice that the ASC device displayed an outstanding specific capacity of 109% retention after 5000 cycles at 3 A g^−1^ (Fig. 45).^232^ This investigation implies the importance of nanoscale MOF electrode materials in the construction of the ASC cell configuration. These results offer a new pathway for developing pristine MOFs for high-performance SCs or other EES devices.

Tang and co-workers demonstrated the preparation of the CNT@UiO-66-AQ electrode and its use in SC applications.^233^ The nitrogen adsorption–desorption analysis was performed to examine the porous structures of MOFs based on UiO-66 and their hybrids in combination with CNTs. A type-I isotherm was observed for UiO-66-NO_2_, exhibiting a SSA of 462.6 m^2^ g^−1^. Alternatively, UiO-66-AQ exhibited a type-IV isotherm, which displayed an SSA of about 129.2 m^2^ g^−1^. Compared to UiO-66-NO_2_, UiO-66-AQ displayed an increase in pore size distribution from 1.7 to 9.1 nm and pore volume enhancement from 0.2 to 0.3 cm^3^g^−1^. The present results suggest that azo-coupled AQ functionalization can enhance the charge storage properties in SC applications. At a current density of 1 mA cm^−2^, the as-fabricated three-electrode device in 1 M H_2_SO_4_ showed a *C*_sp_ of 302.3 mF cm^−2^. Moreover, the CNT@UiO-66-AQ composite electrode displayed negligible *C*_sp_ loss over 5000 GCD cycles. CNT@UiO-66-AQ as a self-standing film electrode in a flexible SSC at 1 mA cm^−2^ displayed the excellent areal capacitance of 155.4 mF cm^−2^. The SSC devices based on the CNT@UiO-66-AQ self-standing film electrodes exhibited a specific energy as high as 0.037 mW h cm^−2^ at a power density of 10.4 mW cm^−2^. The device exhibited a *C*_sp_ retention of 71.9% after 10 000 GCD cycles. This MOF-based electrode material paves the new way to fabricate electrode materials with higher electrochemical performances. In summary, redox-active azo-containing small molecules, polymers COFs and MOFs are promising materials for energy storage systems. Their higher charge-storage capabilities can be attributed to their redox-active –N=N– functional groups. Further investigation and optimization will produce even higher-performing SCs based on azo-contacting materials.

Azo compounds, their polymers, COFs and MOFs have been applied as electrode materials in the development of pseudocapacitors (Table 7). The composite electrode PANI/GO-Azo^217^ in a two-electrode ASC cell configuration displayed the highest *C*_sp_ of 296.36 F g^−1^ at 0.5 A g^−1^ among the reported azo-based small molecules. In contrast, the bisazo-rGO-3electrode in an SSC device exhibited the highest energy density of 49.5 W h kg^−1^.^217^ The AZO-BQ-P^228^ polymer embedded with azo subunits was proven to exhibit the highest *C*_sp_ of 200 F g^−1^ at 0.5 A g^−1^ and excellent energy density of 25.00 W h kg^−1^ at the power density of 900.00 W kg^−1^. In addition, nano-NiMOF-3 in an ASC device displayed 38.65 F g^−1^ at 0.5 A g^−1^ with a reasonable energy density of 13.74 W h kg^−1^.^232^ Thus, the azo-based small molecules and polymers displayed better results compared to MOFs.

**Table 7:** Comparison of the electrochemical properties of azo-based small molecules, polymers, covalent organic frameworks (COFs), metal organic frameworks (MOFs)

Compound code	Electrolyte	Type of working electrode	Specific capacitance (*C*_sp_)	Energy density (ED)	Power density (PD)	Ref.
**Azo-based small molecules**						
P-Azo	Polyacrylamide ion gel	Two-electrode flexible ASC	1195.88 mF cm^−2^ at 0.1 mA cm^−2^	425.2 mW h cm^−2^	80 mW cm^−2^	212
AZOAQ/GF	1 M H_2_SO_4_	Two-electrode SSC	159.12 F g^−1^ at 0.5 A g^−1^	28.64 W h kg^−1^	1080.02 W kg^−1^	214
Bisazo-rGO-3	PVA/H_2_SO_4_ gel	Two-electrode SSC	131.2 F g^−1^ at 1 A g^−1^	30.8 W h kg^−1^	845 W kg^−1^	215
	Acetonitrile (AN) solution of (EMIMBF_4_)	Two-electrode SSC	About ∼201 F g^−1^ at 1 A g^−1^ see Fig. 5b	49.5 W h kg^−1^	1350 W kg^−1^	
Azo-C_*n*_	0.1 M Na_2_SO_4_	Three electrode	204.5 F g^−1^ at 5.0 A g^−1^	13.9 W h kg^−1^	1.5 kW kg^−1^	216
PANI/GO-Azo	1 M H_2_SO_4_	Three electrode	426 F g^−1^ at 0.25 A g^−1^	—	—	217
		Two-electrode ASC	296.36 F g^−1^ at 0.5 A g^−1^	12.45 W h kg^−1^	274.9 W kg^−1^	
Zinc metal-2:1-900	6 M KOH	Three electrode	266.2 F g^−1^ at 1 A g^−1^	—	—	218
	[EMIm]BF_4_ in acetonitrile (AN)	Two-electrode ASC	—	33.4 W h kg^−1^	0.5 kW kg^−1^	
Im-CNT/PPy	1 M H_2_SO_4_	Three electrode	305 F g^−1^ at 1 A g^−1^	—	—	219
AA3/PANI/HCl	0.1 M KCl/Ethanol	Three-electrode.	816.9 F g^−1^ at 50 mV s^−1^	—	—	221

**Azo based polymers**						
Py-*m*Azo-CMP and Py-*p*Azo-CMP	1M KOH	Three electrode	142 F g^−1^ and 93 F g^−1^, respectively at 1 A g^−1^	19.72 W h kg^−1^ and 12.92 W h kg^−1^, respectively	For PD, see Fig. 6	227
Py-*m*Azo-CMP and Py-*p*Azo-CMP		Two-electrode SSC	17 and 28 F g^−1^, respectively, at 2 A g^−1^	2.36 and 3.88 W h kg^−1^, respectively	For PD, see Fig. 6	
AZO-BQ-P	1 M H_2_SO_4_	Two-electrode SSC	200 F g^−1^ at 0.5 A g^−1^	25.00 W h kg^−1^	900.00 W kg^−1^	228

**Azo based COFs**						
TFPB-AZO-COF	1 M KOH	Two-electrode ASC	61.4 F g^−1^ at 1 A g^−1^	24.6 W h kg^−1^ at 10 A g^−1^	8.5 kW kg^−1^ at 10 A g^−1^	230
TFPA-AZO-COF			24.47 F g^−1^ at 1 A g^−1^	9.8 W h kg^−1^ at 10 A g^−1^	8.6 kW kg^−1^ at 10 A g^−1^	

**Azo-based MOFs**						
Nano-NiMOF-3	1.0 M KOH	Three electrode	1024 F g^−1^ at 1 A g^−1^	—	—	232
		Two-electrode ASC	38.65 F g^−1^ at 0.5 A g^−1^	13.74 W h kg^−1^	400 W kg^−1^	
CNT@UiO-66-AQ	1 M H_2_SO_4_	Flexible SSC	155.4 mF cm^−2^	0.037 mW h cm^−2^	10.4 mW cm^−2^	233

### 4.6. Ferrocene-based organic materials for SCs

#### 4.6.1. Redox properties of ferrocene

**Fig. 46:**
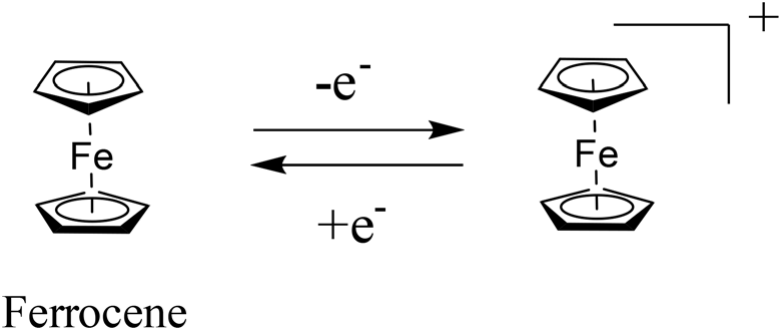
Redox reaction of ferrocene.

The ferrocene structure is comprised of two negatively (−vely) charged aromatic cyclopentadienyl (Cp) rings and Fe(ii) ions (Fig. 46). The Cp ring subunit generates a stable 18-electron system and forms coordination complexes with Fe(ii) ions.^234^ Ferrocene exhibits good solubility in organic solvents and displays thermal and photochemical stability.^235^ Ferrocene displayed excellent reversible redox reactions (Fig. 46) at the redox potential of +0.403 V *vs.* a saturated calomel electrode (SCE) in organic solutions.^236^ Owing to the easy handling of low-cost ferrocene with versatile redox-active properties, it has been utilized in biological sensing, nanomedicine, catalysis and battery applications.^237^ It is noticeable that the functionalization of ferrocene structures is easy. This structural manipulation can provide increased research density towards diverse molecular materials with a wide variety of applications.^238^ In this context, ferrocene is an important building block utilized in various applications related to the electrochemistry field.^239^

**Fig. 47:**
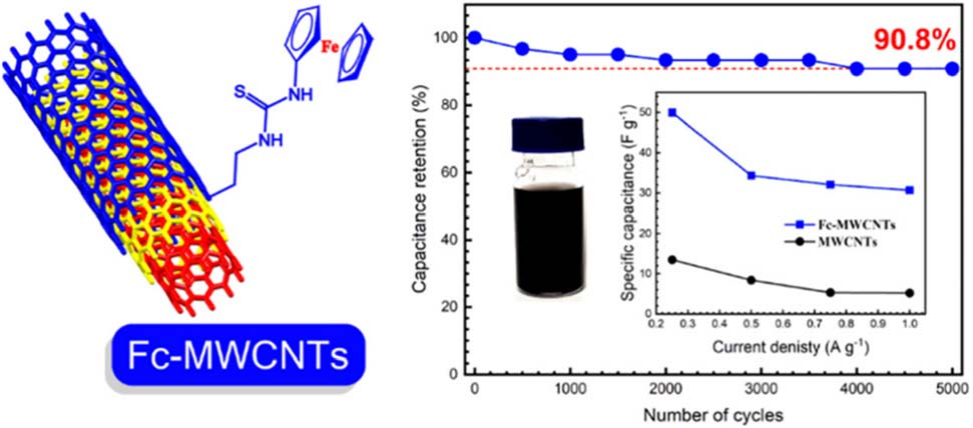
Structural unit of ferrocene-functionalized MWCNTs and capacitance retention after 5000 GCD cycles (inset shows the variation of *C*_sp_ with current density). Reproduced from ref. 242 with permission from [Elsevier], Copyright [2020].

The redox-active ferrocene can be utilized as an effective electron-transfer mediator.^240^ Moreover, the ferrocene molecular entity with a lower oxidation potential and electrochemical behavior can be employed as a redox probe in energy storage applications.^239*b*,241^ In this context, Chong and co-workers demonstrated the utilization of redox-active ferrocene-modified multiwall carbon nanotubes (MWCNTs) (Fig. 47) for supercapacitor applications.^242^ The electrochemical performance of the Fc-MWCNT electrode was investigated by GCD measurements at 0.25 A g^−1^, showing the *C*_sp_ of about 50 F g^−1^, which was higher than that of the pristine MWCNT-NH_2_ electrode (13 F g^−1^). The Fc-MWCNT electrode displayed reversible faradaic redox-behaviour at the electrolyte/electrode interface.^242^ At 2 A g^−1^, the as-fabricated Fc-MWCNTs displayed outstanding cycling life with 90.8% retention of its initial *C*_sp_ after 5000 GCD cycles (Fig. 47). These charge-storage results shown by Fc-MWCNTs make these materials as promising electrodes for next-generation EES.

**Fig. 48:**
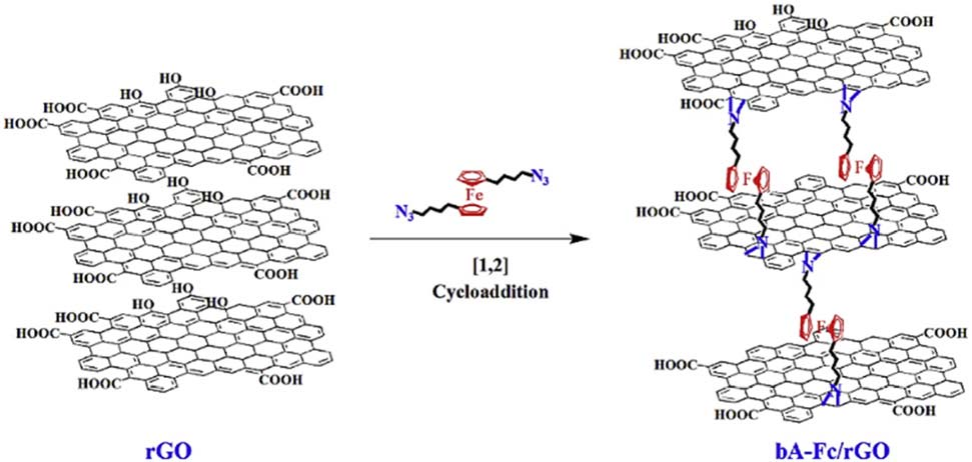
Synthetic method for the preparation of bA-Fc/rGO. Reproduced from ref. 246 with permission from [Elsevier], Copyright [2019].

Hadi *et al.* reported the synthesis of 4-azidobutylferrocene (AzFc)-anchored rGO and its nanocomposite with PANI and its use as a battery-type SC material.^243^ The rGO, AzFc/rGO and AzFc/rGO/PANI electrodes in 1 M H_2_SO_4_ electrolyte displayed the charge storage capacity of 14, 70 and 95 mA h g^−1^ at a current density of 14 A g^−1^, respectively. Moreover, the AzFc/rGO/PANI electrode exhibited 89% *C*_sp_ retention over 2000 cycles of CV and acted as a promising new battery-type supercapacitor electrode material.^243^ In recent years, zinc ion hybrid SCs (ZHSCs) have appeared as low-cost electrode materials for energy storage applications on a large scale.^244^ In this case, to develop SCs with economic feasibility and high energy density, We and co-workers reported the fabrication of a hybrid SC device based on ferrocene with hydrazide activated carbon (AC) to yield the new electrode material ferrocene/AC as the cathode and Zn-ion as the anode.^245^ As-fabricated hybrid ZHSCs supercapacitor cell based on the ferrocene/AC electrode displayed an impressive electrochemical performance compared to the pristine ferrocene cathode material. The ZHSC cell configuration exhibited faster pseudocapacitive reaction kinetics. delivering *C*_sp_ of 125.1 F g^−1^ with an energy density as high as 44.8 W h kg^−1^ at 0.1 A g^−1^ and power density as large as 1839 W kg^−1^ at a current density of 5 A g^−1^. The device showed 73.8% *C*_sp_ retention over 10 000 GCD cycles. It is noticeable that the ferrocene/AC cathode material in the ZHSC device yielded a good electrochemical performance at the lower temperature of −30 °C. This investigation can help understand the properties of inorganic–organic hybrid electrode materials and their utilization in SCs. A novel ferrocene-grafted reduced graphene oxide (rGO) material was developed to fabricate the bA-Fc/rGO nanocomposite electrode (Fig. 48).^246^ The bA-Fc/rGO electrode (Fig. 48) in an SC device in aqueous 1 M H_2_SO_4_ electrolyte displayed a higher performance compared to the bare rGO and bA-Fc-based materials.

**Fig. 49:**
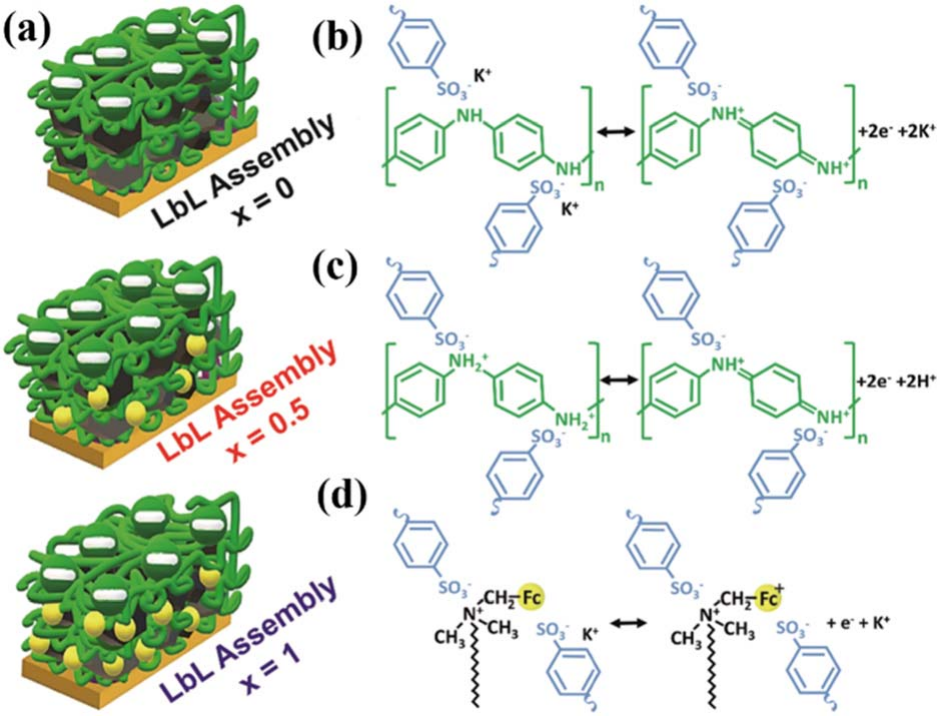
(a) Schematic representation of the assemblies fabricated for the three systems; systematic presentation of the ionic exchange in the electrochemical cycling for LbL assembly, (b) PANI-PSS in KCl, (c) PANI-PSS in HCl and (d) FcCTAB in KCl. Reproduced from ref. 252 with permission from RSC.

Although pseudocapacitive electrode materials provide a high *C*_sp_ and energy density, but they also suffer from a low rate capability and conductivity. Thus, to overcome these limitations, electrode materials from two groups are utilized.^247^ In this context, MnO_2_ and different carbon-based electrode materials are used to enhance the *C*_sp_ of SC devices.^248^ Moreover, ferrocene-functionalized MnO_2_ was used to synthesize MnO_2_-Fc/CA nanocomposites.^249^ The MnO_2_-Fc/CA-based electrode compared to nonfunctionalized nanoparticles displayed a good electrochemical performance.^249^ MnO_2_-Fc/CA exhibited an excellent *C*_sp_ value of 963 F g^−1^ at 1 A g^−1^ and outstanding cycling life of about 96% after 3000 CV cycles. The SSC device revealed the highest energy density of 38.1 W h kg^−1^ at a power density of 1232 W kg^−1^, indicating that the MnO_2_-Fc/CA nanocomposite is an attractive material for SC applications. Recently, Boota *et al.* reported the ferrocene (Ferro) and decamethylferrocene (DFerro)-functionalized GP for SC applications.^250^ They found that the electrochemical properties of Ferro@rGO and DFerro@rGO are inferior compared to *N*,*N*,*N*′,*N*′-tetramethyl-*p*-phenylenediamine (TMPD)@rGO electrode materials. This could be attributed to the fact that TMPD can exfoliate graphite into few-layer graphene sheets and act as a dopant during the reduction process. The obtained n-doped material based on TMPD@rGO showed an excellent performance in SCs. A carbon nanotube/chitosan-ferrocene nanocomposite (CNTs/Cs-Fc) was prepared and successfully utilized in SC devices in 1 M H_2_SO_4_ electrolyte.^251^ The CNT/Cs-Fc-based SC device showed *C*_sp_ of 695 F g^−1^ at 1 A g^−1^, which is about nine-fold higher than that of the bare CNT (75 F g^−1^)-based electrode material. Moreover, the CNT/Cs-Fc nanocomposite displayed 99.93% retention of its initial *C*_sp_ value after 2000 cycles of CV. Marmisollé and co-workers utilized layer-by-layer assembly to construct the PANI-PSS/nanocarbon nanomaterial through different fabrication processes.^252^ They used 1-hexadecyltrimethylammonium bromide (CTAB)^253^ as well as ferrocene-labelled surfactant (FcCTAB) to construct the electroactive materials for EES applications (Fig. 49). To explore the contribution from ferrocene to the charge-storage performance, they utilized nanoarchitectonics for the fabrication of three assemblies based on various ferrocene-labelled surfactants (*x* = 0, 0.5 and 1) (Fig. 49a). The nanocarbon dispersed with FcCTAB/PANI-PSS (Au electrode) in aqueous 0.1 M KCl displayed the *C*_sp_ of 423.75 F g^−1^ at a current density of 1.5 A g^−1^ and retained 125.0 F g^−1^ at 10 A g^−1^. The PANI-poly(vinylsulfonate) films at lower pH exhibited a proton exchange as the dominant process, whereas anion insertion process became important at intermediate pH values.^254^ Alternatively, PANI-PSS under acidic conditions exhibited excess negative charge. At pH 7, the *ζ*-potential value of PANI-PSS was estimated to be −21.6 mV, while it was −13.8 mV in 0.5 M HCl. Therefore, in PANI-PSS complex building blocks, charge compensation takes place *via* cation transportation.^255^ As shown in Fig. 49b, when reduction occurs, K^+^ goes into the PANI-PSS film to neutralize the excess −ve charge. In contrast, the cations migrate from the film to solution during the oxidation process. In 0.1 M HCl, the cation movement was confirmed by means of identical measurements (Fig. 49c), where charge balance was achieved by cation movement between the PANI-PSS film and solution. As illustrated in Fig. 49d, a reduction in mass was observed during the oxidation process although the ferrocene chemistry dominated the redox kinetics. Herein, the LbL assemblies containing FcCTAB displayed the charge compensation by the ferrocenium subunits in the presence of excess PSS moieties (Fig. 49d).

**Fig. 50:**
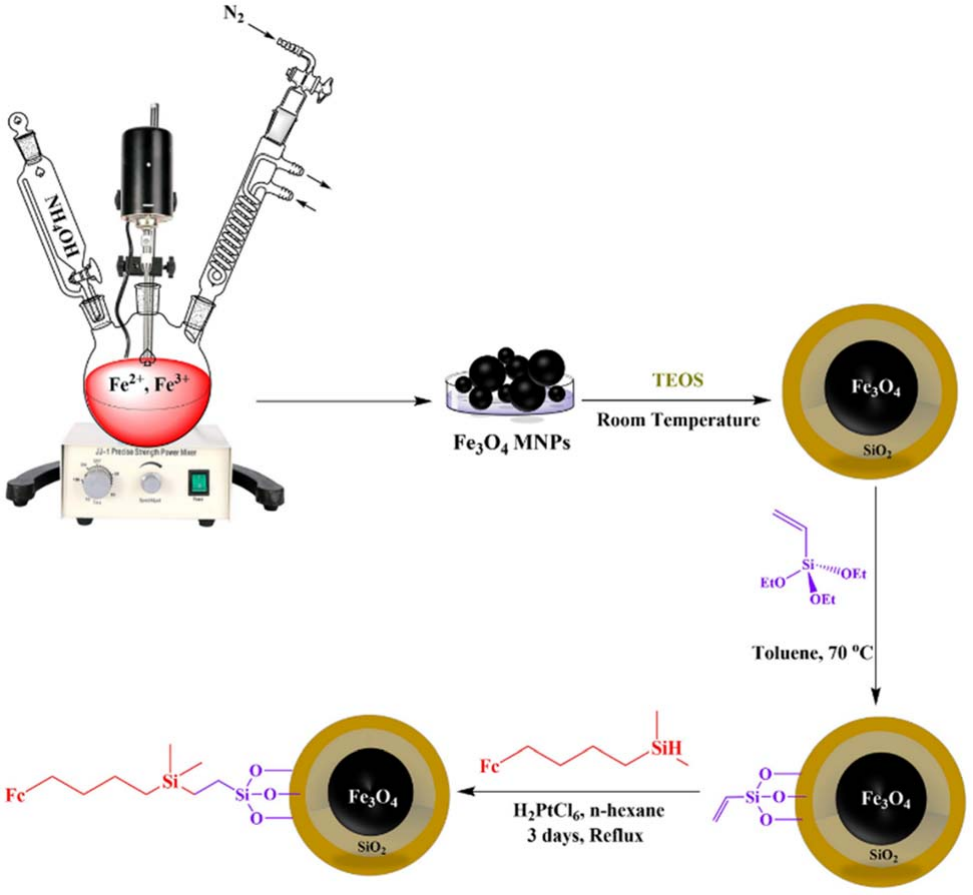
Route for the preparation of Fe_3_O_4_@SiO_2_@Fc. Reproduced from ref. 256 with permission from [Elsevier], Copyright [2024].

The PANI-PSS-based electrode systems store charge by means of EDLC and faradaic redox processes and enhanced the energy storage characteristics of the cell. It is important to note that the ferrocene-labelled surfactant (FcCTAB) added the third dimension of redox capacitance to the LbL assembly. The present investigation demonstrated that the ferrocene-labelled surfactant significantly contributed to the electrochemical connectivity within the LbL assemblies. Moreover, the authors stated that the integration of the ferrocene-labelled surfactant significantly enhanced the chare storage performance of the devices in neutral solution without disturbing their structural stability and cycling life. Therefore, the present cell fabrication approach using electroactive polymers and nanocarbon materials in combination with the redox active surfactant led to the newer creation of SC electrode materials with excellent electrochemical performances in aqueous neutral solution. The redox-active (4-ferrocenylbutyl)dimethylsilane was used for the functionalization of the surface of magnetite nanoparticles (MNPs) to fabricate the novel Fe_3_O_4_@SiO_2_@Fc (Fig. 50) electrode material.^256^

The BET analysis based on the N_2_ adsorption–desorption study of the Fe_3_O_4_ and Fe_3_O_4_@SiO_2_@Fc electrode materials displayed type-IV isotherms. The N_2_ adsorption–desorption isotherm of Fe_3_O_4_@SiO_2_@Fc displayed a hysteresis loop, indicating that this material has abundant mesoporosity. The presence of mesopores in Fe_3_O_4_ and Fe_3_O_4_@SiO_2_@Fc was also confirmed by means of BJH plots. The BET parameter of SSA was determined to be 73.45 m^2^ g^−1^ and 198.34 m^2^ g^−1^ for Fe_3_O_4_ and Fe_3_O_4_@SiO_2_@Fc, respectively. The increase in mesopores during the preparation of the Fe_3_O_4_@SiO_2_@Fc framework played a crucial role in the development of the electrode materials. At 2.5 A g^−1^, Fe_3_O_4_@SiO_2_@Fc exhibited a specific capacity of 161 mA h g^−1^, which was higher compared to that of pure Fe_3_O_4_ (71 mA h g^−1^). The Fe_3_O_4_@SiO_2_@Fc electrode-based SC configuration exhibited an energy density as high as 96.6 W h kg^−1^ at a power density of 1473 W kg^−1^. The cycling performance displayed by the Fe_3_O_4_@SiO_2_@Fc electrode was as high as 84.6% after 3000 GCD cycles. This electrode material based on ferrocene and magnetic nanoparticles can be utilized in next-generation charge storage devices.

**Fig. 51:**
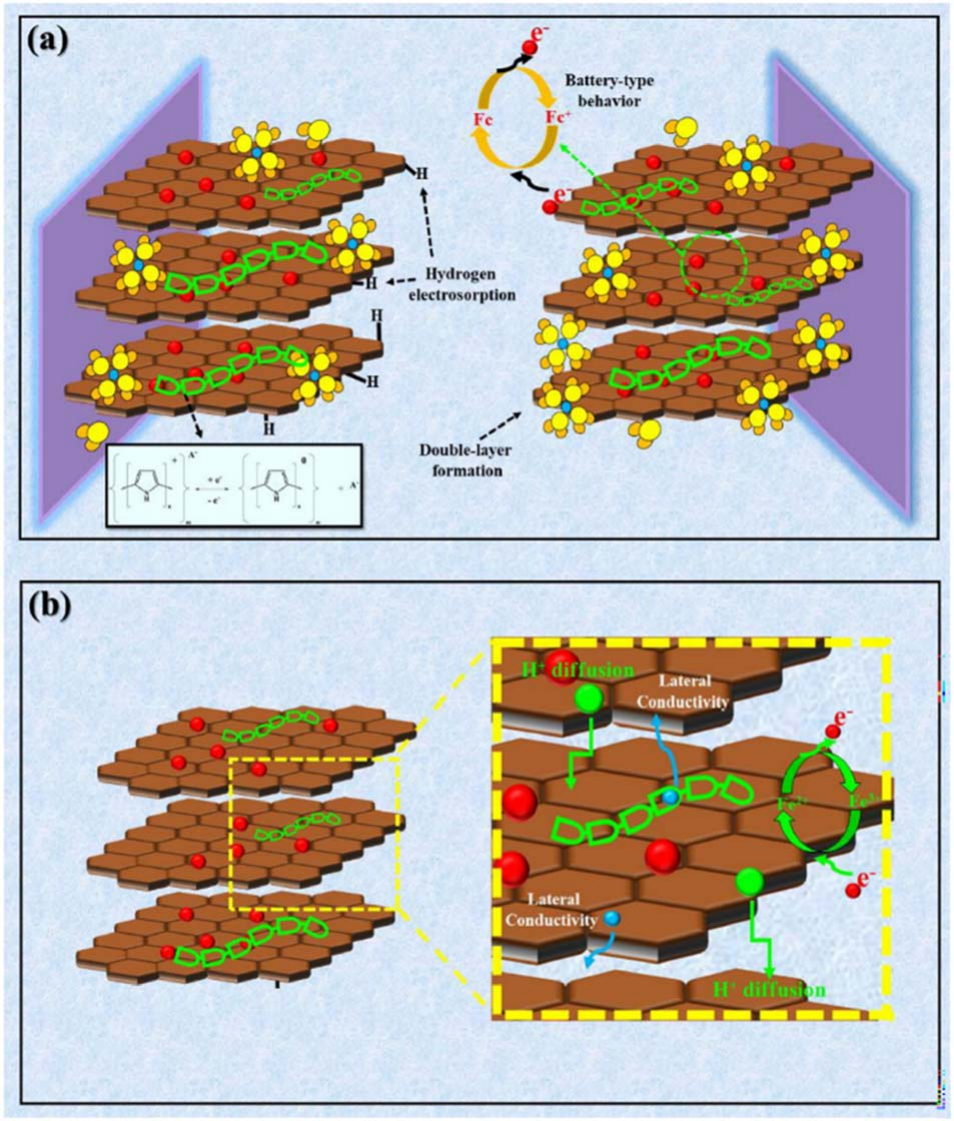
(a) Schematic presentation the hybrid charge storage mechanisms and (b) exact electron transfer in the nanocomposite. Reproduced from ref. 259 with permission from [Elsevier], Copyright [2024].

A ferrocene-functionalized graphene nanoribbon, dicationic ionic liquid, and poly (*o*-aminophenol) nanocomposite, abbreviated as GNR-Fc/DCIL/PoAP-NC, was synthesized and used for battery-type SC applications.^257^ The GNR-Fc and GNR-Fc/DCIL/PoAP-NC electrodes in a three-electrode cell SC device at 1 A g^−1^ displayed a *C*_sp_ of 208 and 395 mA h g^−1^, respectively. Moreover, the GNR-Fc/DCIL/PoAP-NC electrode-based device delivered an energy density as high as 29.625 W h kg^−1^ at a power density of 852 W kg^−1^. Its electrochemical performance could be ascribed to the synergistic effects of NC, lowering the charge transfer resistance and expanding the charge transfer characteristics of GNR. The ferrocene-based Fc–Phe–Phe–propyne self-assembled material was utilized to functionalize a carbon electrode and successfully employed in supercapacitor applications.^258^ To achieve a higher performance in the SC device, ferrocene-modified graphene oxide (GO) nano-hybrids, denoted as GO-FBF1/PPy and GO-FBF2/PPy, were prepared.^259^ The BET analysis of the as-prepared electrode materials was studied using nitrogen adsorption and desorption. The SSA of GO, PPy, GO-FBF1, GO-FBF2, GO-FBF1/PPy, and GO-FBF2/PPy was found to 21.403, 47.533, 33.305, 37.203, 45.197 and 49.289 m^2^ g^−1^, respectively.^259^ According to the data, it was observed that the electrode materials with an increase in FBP to GO ratio possessed a higher surface area. The as-fabricated ferrocenyl composite materials displayed type-I isotherms, indicating the presence of mesoporous structures with a smaller width. The average pore diameter values of GO, PPy, GO-FBF1, GO-FBF2, GO-FBF1/PPy, and GO-FBF2/PPy estimated using the BJH method were 4.0527, 5.0984, 12.265, 10.786, 10.192 and 35.218 nm, respectively. The larger surface area and the mesoporous range of the GO-FBF1/PPy and GO-FBF2/PPy electrode structures resulted in abundant electroactive sites, which can be advantageous for high-performance charge-storage. The GO-FBF1/PPy and GO-FBF2/PPy nanocomposite electrodes in SC application in 1 M Na_2_SO_4_ at 1 A g^−1^ displayed the charge storage capacity of 229.43 and 269.57 mA h g^−1^, respectively.^259^ Both the GO-FBF1/PPy and GO-FBF2/PPy nanocomposite electrodes retained *C*_sp_ of 91% and 93%, respectively, after 5000 CV cycles. The GO-FBF2/PPy//GO-FBF2/PPy SSC cell in the applied voltage window of 1.6 V showed the *C*_sp_ of 78 mA h g^−1^ at a current density of 1 A g^−1^. Moreover, the SSC device showed an exceptional cycling life with 95% retention of its initial *C*_sp_ value after 3000 cycles. The as-fabricated GO-FBF2/PPy//GO-FBF2/PPy SSC device exhibited energy and power densities as high as 123 W h kg^−1^ and 7859 W kg^−1^, respectively.^259^ The hybrid charge storage mechanism is demonstrated in Fig. 51a.^259^ After reduction at the cathode surface, the H^+^ ions are rapidly adsorbed on the surface of the carbon material. The sharp increase in the anodic current after the adsorption of the hydrogen can be attributed to the anodic oxidation of the adsorbed hydrogen. The battery-type redox-properties were observed based on the redox peaks in the anodic and cathodic scans. The higher capacity of the device was observed due to the repeated redox-reactions at the cathode surface. The electron transfer process is displayed in Fig. 51b.^259^ The charge separation and transfer were observed due to the layered GO structure and the nanocomposite porous structures (Fig. 51b). Herein, the SCs showed an improvement in electrochemical performance and acted as battery-type electrode materials. The exceptional stability of the electrode nanocomposites is ascribed to the synergistic effects between GO nanosheets and ferrocene-containing material and polypyrrole polymers. The GO layered structure provides mechanical strength to the electrode, helping to enhance the cycling stability. The stacking of the GO layer was prevented by the polypyrrole and FC-containing material in the nanosheets.

#### 4.6.2. Ferrocene-based polymers for SCs

**Fig. 52:**
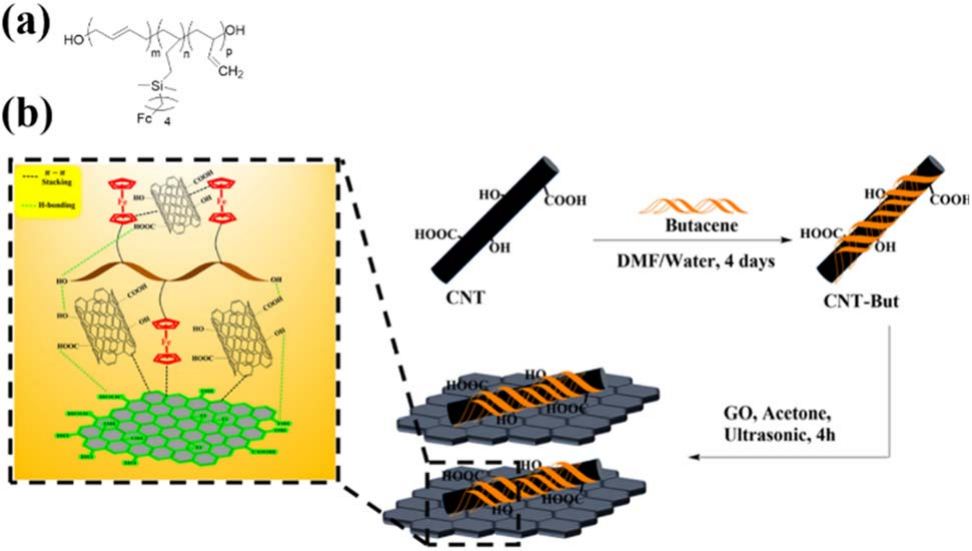
(a) Structure of Fc-appended butacene polymer and (b) preparation of final CNT-But/GO nanocomposites. Reproduced from ref. 263 with permission from [Elsevier], Copyright [2022].

In recent years, conducting polymers with faradaic behaviour in combination with carbon-based nanocomposites^260^ have attracted attention from researchers to fabricate high-performance SCs.^261^ The incorporation of conducting polymers in the carbon nanotube template facilitates the reversible insertion-deinsertion of ions, which in turn enhances the specific capacity of battery-type SC devices. In recent years, ferrocene-based materials, which display fast electron transfer kinetics and excellent charge/discharge behaviour, have been utilized in EES applications.^262^ To improve the power and energy densities, ferrocene (Fc)-containing butacene polymers (Fig. 52a) could be incorporated into CNTs. The Fc-functionalized CNTs were intercalated in the GO layer (Fig. 52b).^263^ Herein, the authors assumed that the design of this material will enhance the redox-response of the electrode by incorporating Fc components and stability due to the presence of carbon-based nanocomposites such as CNT and GO. The Fc-flanked polymer was incorporated in CNTs by π–π stacking interactions and H-bonding interaction between the CNTs and butacene (Fig. 52b). The as-fabricated Fc-bearing electrode materials such as CNT-But, CNT-But/GO_0.5:1_, CNT-But/GO_1:1_, and CNT-But/GO_3:1_ samples were examined for their SC applications in a three-electrode SC system in 1 M H_2_SO_4_ electrolyte solution. The CNT-But, CNT-But/GO_0_._5:1_, CNT-But/GO_1:1_, and CNT-But/GO_3:1_ tested materials displayed redox peaks, confirming their battery-type electrode properties. The CNT-But/GO_1:1_ electrode in 1 M H_2_SO_4_ electrolyte showed a specific capacity of 456 mA h g^−1^ at a current density of 2.5 A g^−1^ together with 96% charge retention after 5000 GCD cycles. The CNT-But/GO_1:1_//CNT-But/GO_1:1_ SSC device displayed a specific capacity as high as 104 mA h g^−1^ at 1 A g^−1^. It was observed that the SSC device exhibited excellent charge storage retention of 91% over 2000 GCD cycles. The maximum energy and power densities for the SSC device were found to be 94.5 W h kg^−1^ and 8370 W kg^−1^, respectively.^263^ The specific capacity of CNTs was significantly enhanced due to the presence of the Fc subunit in the polymer chain, and also showed battery-type properties in the device. Therefore, this CNT-But/GO_1:1_ nanocomposite electrode appears to be a promising energy storage material.

**Fig. 53:**
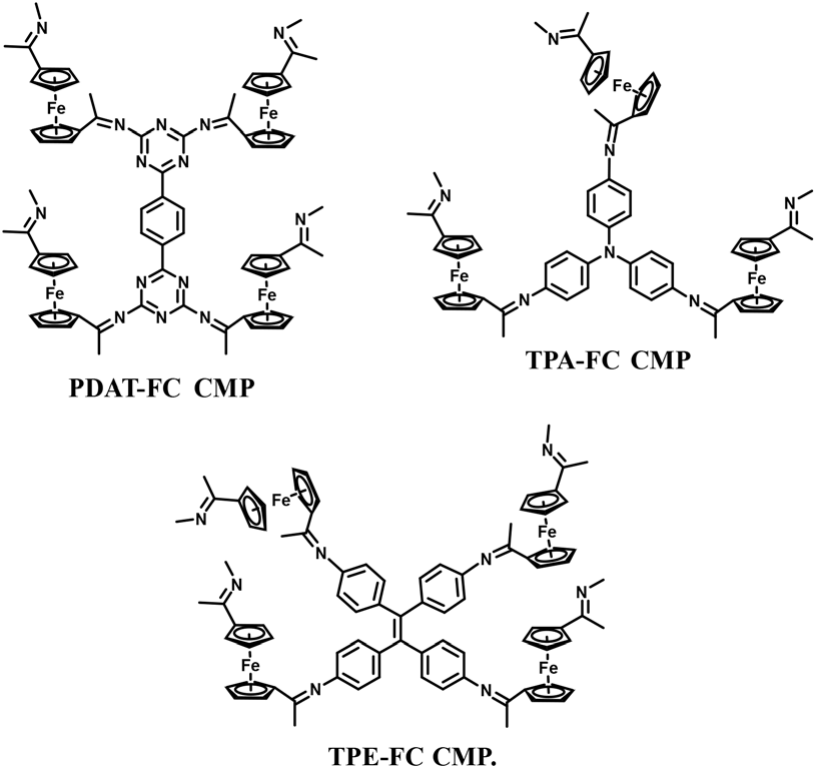
Structures of PDAT-FC CMP, TPA-FC CMP, and TPE-FC CMP.

Morsali and co-workers demonstrated the synthesis and charge-storage applications of redox-active Fc-modified (Fc(COOH)_2_) clusters based on coinage metals, [(PPh_3_)_2_AgO_2_CFcCO_2_Ag(PPh_3_)_2_]_2_·7CH_3_OH (SC1) and [(PPh_3_)_3_CuO_2_CFcCO_2_Cu(PPh_3_)_3_]·3CH_3_OH (SC2), as next-generation SC electrodes.^264^ The coordinated polymer electrode materials based SC1 and SC2 in Na_2_SO_4_ electrolyte delivered the *C*_sp_ of 130 F g^−1^ and 210 F g^−1^ at 1.5 A g^−1^, respectively. The charge-storage results displayed that the presence of Cu^I^ and Ag^I^ enhances the electrochemical performance of the SCs. They also reported an enhanced electrochemical performance using the polymeric structure PSC_2_ ([(PPh_3_)_2_CuO_2_CFcCO_2_]_∞_), which displayed the *C*_sp_ of 455 F g^−1^ at 3 A g^−1^ with energy and power densities of 161 W h kg^−1^ and 2416 W kg^−1^, respectively, and cycling stability of 93% after 4000 cycles. This coordinate polymer design without hybridization and additive material composition can lead to the amplification of the performance of SCs. Conjugated microporous polymers (CMPs) such as PDAT-FC CMP, TPA-FC CMP, and TPE-FC CMP (Fig. 53) bearing ferrocene subunits were utilized for SC applications.^265^ Nitrogen sorption measurements (adsorption/desorption isotherms) were performed at 77 K to examine the porosity of the FC CMPs (PDAT-FC, TPA-FC, and TPE-FC CMPs). All three electrode materials exhibited type-IV adsorption isotherms, indicating the presence of both micropores and mesopores (hysteresis loops) within their polymeric framework. The BET analysis of PDAT-FC, TPA-FC, and TPE-FC CMPs displayed the SSA of 502, 701, and 100 m^2^ g^−1^, respectively. The estimated pore size distribution for PDAT-FC CMP, TPA-FC CMP and TPE-FC CMP was 1.11–4.80 nm, 1.16–3.90 nm and 1.83–4.10 nm, respectively. Among them, PDAT-FC CMP and TPA-FC CMP exhibited the high surface area of 502 and 701 m^2^ g^−1^. It was observed that among the as-prepared electrode materials, the TPA-FC CMP electrode demonstrated the highest *C*_sp_ of 129 F g^−1^ with 96% retention over 5000 cycles. The good performance of TPA-FC CMP is ascribed to the redox-active ferrocene and triphenylamine moieties. Moreover, the higher surface area and good porosity of the TPA-FC CMP are responsible for the rapid redox-activity of the electrode in SCs.

Ferrocene-based redox-active conjugated macroporous polymers (CMPs) in combination with rGO in the 2D porous framework were used to construct electrode materials for SC applications.^266^ The BET analysis using N_2_ sorption isotherm measurements of the porous CMPs, CMPs/rGO, pure Fc-CMPs and Fc-CMPs/rGO polymers exhibited type-I isotherm characteristics, indicating the microporous structure of the materials. The calculated BET SSA for the pure CMPs, CMPs/rGO, pure Fc-CMPs and Fc-CMPs/rGO was 672.3, 668.3, 653.2 and 800.1 m^2^ g^−1^, respectively. The as-prepared FcCMPs/rGO composite electrode in three-electrode and two-electrode systems displayed the *C*_sp_ of 470 F g^−1^ (933 mF cm^−3^) and 231 F g^−1^ (238 mF cm^−3^), respectively, at a current density of 0.5 A g^−1^. Moreover, after 8000 GCD cycles, FcCMPs/rGO showed 95% *C*_sp_ retention. The higher performance of the SCs was ascribed to the synergistic effects exhibited by the highly conducting rGO and the redox-active Fc-CMPs. The redox-active porous Fc-CMPs provided a higher surface area, and also facilitated faster electrolyte transfer. This combination of the redox-active polymer and conducting rGO offers an opportunity for the design and construction of highly efficient electrode materials with better electrochemical properties. Similarly, different ferrocene-functionalized CMPs have been utilized for SC applications with high specific capacitance and capacity retention.^267^

#### 4.6.3. Ferrocene-based COFs for SCs

Covalent organic frameworks (COFs) with excellent structural stability are beneficial for examining their utilization in EES applications. To the best of our knowledge, ferrocene-based conjugated microporous polymers (CMPs) have been reported thus far, but we could not trace COF materials based on ferrocene for supercapacitor applications.

#### 4.6.4. Ferrocene-based MOFs for SCs

**Fig. 54:**
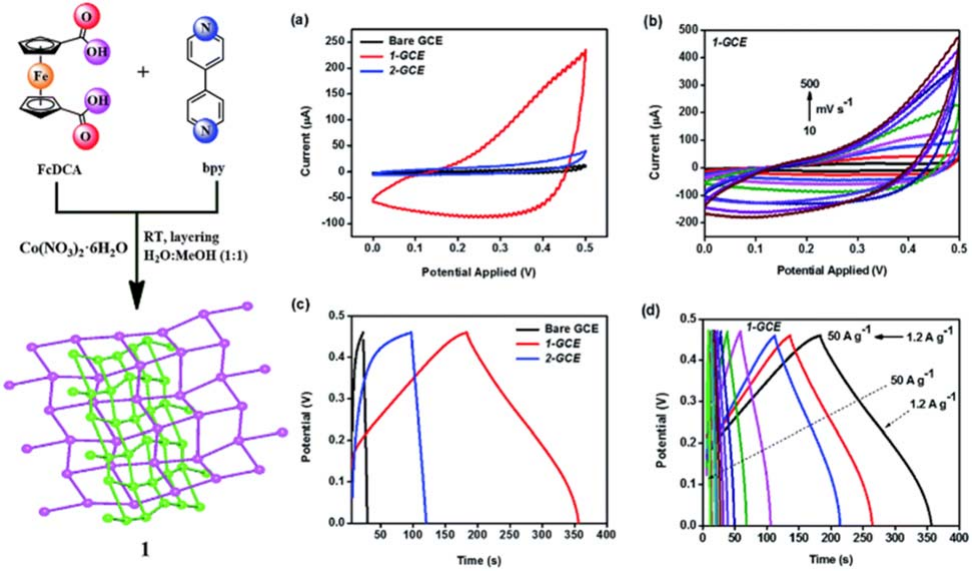
Structure of Fc-MOF-1 and (a) CV curves of base GCE, 1-GCE and 2-GCE at 100 mV s^−1^; (b) CV curves of 1-GCE at different scan rates (10 to 500 mV s^−1^), (c) GCD profiles of base GCE, 1-GCE and 2-GCE at 1.2 A g^−1^; and (d) GCD profiles of 1-GCE at different current densities ranging from 1.2 to 50 A g^−1^ in 1 M KOH electrolyte solution. Reproduced from ref. 268 with permission from RSC.

To tackle the challenges associated with the fabrication of next-generation SCs, metal organic frameworks (MOFs) have attracted attention from researchers due to their tunable properties. In this regard, MOFs based on ferrocene subunits are an attractive choice. Therefore, to achieve long-term energy storage, the ferrocene-based {[Co_4_(FcDCA)_4_(bpy)_4_(H_2_O)_6_]·11H_2_O}_*n*_ [FcDCA = 1,1′-ferrocene dicarboxylic acid and bpy = 4,4′-bipyridyl] (Fc-MOF-1) MOF was designed and successfully utilized in SCs (Fig. 54) by Mobin and co-workers.^268^ The active MOF material Fc-MOF-1 was used to modify a glassy carbon electrode without using any binder. Subsequently, the 1-GCE electrode was explored for SC applications. 1-GCE exhibited a *C*_sp_ of 446.8 F g^−1^ at a current density of 1.2 A g^−1^ (Fig. 54), and also showed cycling stability of ∼88.37% after 800 cycles. The higher specific capacity of 1-GCE could be attributed to the improved electronic conductivity and increased contact at the electrolyte–electrode interface. The faster ion diffusion due to the shorter path improved the energy storage efficiency.^269^ The superior results revealed from the 1-GCE material compared to {[Co(bpy)_1.5_(NO_3_)_2_]}_*n*_ (2-GCE) suggest the importance of the presence of FcDCA in 1-GCE.^268^

Ferrocenes have also been applied in pseudocapacitor applications (Table 8). The redox-activity of these electrode materials arise from the ferrocene part of the composite. Among the reported electrode materials based on ferrocene small organic molecules, ferrocene/AC^245^ displayed the highest *C*_sp_ of 125.1 F g^−1^ at 0.1 A g^−1^ and excellent energy density of about 44.8 W h kg^−1^ at 0.1 A g^−1^. It is noticeable that the GO-FBF2/PPy^259^ electrode in an SSC device delivered an outstanding energy density of 123 W h kg^−1^. The high energy density performance of the ferrocene/AC^245^ and GO-FBF2/PPy^259^ electrodes make them attractive materials for commercial applications. Ferrocene based polymers and COFs have been utilized in electrode materials in PSCs. In pseudocapacitors, BP-FC-CMP^267^ revealed the highest *C*_sp_ of 608 F g^−1^ at 0.5 A g^−1^. Besides the superior charge-storage capacity, BP-FC-CMP^267^ displayed the outstanding energy density of 87.45 W h kg^−1^ at a power density of 250 W kg^−1^. However, the major limitation of this electrode is that the authors did not report the fabrication of two-electrode SSC or ASC devices, which can help test the real-world application of this material. The 1-GCE^268^ MOF also exhibited the excellent *C*_sp_ of 446.8 F g^−1^ at 1.2 A g^−1^. Thus, ferrocene electrode materials have been proven to be a promising class of faradaic redox-active materials for SCs.

**Table 8:** Comparison of the electrochemical properties of ferrocene-based small molecules, polymers, covalent organic frameworks (COFs), and metal organic frameworks (MOFs)

Compound code	Electrolyte	Type of working electrode	Specific capacitance (*C*_sp_)	Energy density (ED)	Power density (PD)	Ref.
**Ferrocene based small molecules**						
Fc-MWCNTs	2 M KOH	Three electrode	50 F g^−1^ at 0.25 A g^−1^	—	—	242
AzFc/rGO	1 M H_2_SO_4_	Three electrode	70 mA h g^−1^ at 14 A g^−1^	—	—	243
AzFc/rGO/PANI			95 mA h g^−1^ at 14 A g^−1^	—	—	
Ferrocene/AC	2 M ZnSO_4_	Two-electrode ASC	125.1 F g^−1^ at 0.1 A g^−1^	44.8 W h kg^−1^ at 0.1 A g^−1^	1839 W kg^−1^ at 5 A g^−1^	245
bA-Fc/rGO	1M H_2_SO_4_	Three electrode	∼132 F g^−1^ at 1 A g^−1^ [see Fig. 8b]	—	—	246
MnO_2_-Fc/CA	1M H_2_SO_4_	Three electrode	963 F g^−1^ at 1 A g^−1^	38.1 W h kg^−1^	1232 W kg^−1^	249
TMPD-rGO	1 M H_2_SO_4_	Three electrode	185 F g^−1^ at 2 mV s^−1^	—	—	250
CNTs/Cs-Fc	1 M H_2_SO_4_	Three electrode	695 F g^−1^ at 1 A g^−1^	—	—	251
FcCTAB/PANI-PSS	0.1 M KCl	Three electrode	423.75 F g^−1^ at 1.5 A g^−1^	—	—	252
Fe_3_O_4_@SiO_2_@Fc	1 M H_2_SO_4_	Three electrode	161 mA h g^−1^ (582C g^−1^) at 2.5 A g^−1^	96.6 W h kg^−1^	1473 W kg^−1^	256
GNR-Fc	1 M H_2_SO_4_	Three electrode	208 mA h g^−1^ at 1 A g^−1^	—	—	257
Fc/DCIL/PoAP-NC			395 mA h g^−1^ at 1 A g^−1^	29.625 W h kg^−1^ at 1 A g^−1^	852 W kg^−1^ at 1 A g^−1^	
GO-FBF1/PPy	1 M Na_2_SO_4_	Three electrode	229.43 mA h g^−1^ at 1 A g^−1^	—	—	259
GO-FBF2/PPy			269.57 mA h g^−1^ at 1 A g^−1^	—	—	
GO-FBF2/PPy	1 M Na_2_SO_4_	Two electrode SSC	78 mA h g^−1^ at 1 A g^−1^	As high as 123 W h kg^−1^	As high as 7859 W kg^−1^	

**Ferrocene-based polymers**						
GO-B-(EtFc)-Pr3/PANI0.5	1 M H_2_SO_4_	Three electrode	429 mA h g^−1^ at 2.5 A g^−1^	—	—	262
CNT-but/GO_1:1_	1 M H_2_SO_4_	Three electrode	456 mA h g^−1^ at 2.5 A g^−1^	—	—	263
		Two-electrode SSC	104 mA h g^−1^ at 1 A g^−1^	Max. 94.5 W h kg^−1^	Max. 8370 W kg^−1^	
PSC_2_([(PPh_3_)_2_CuO_2_CFcCO_2_]_∞_)	1 M Na_2_SO_4_	Three electrode	455 F g^−1^ at 3 A g^−1^	161 W h kg^−1^	2416 W kg^−1^	264
PDAT-FC CMP	1 M KOH	Three electrode	102 F g^−1^ at 0.5 A g^−1^	14 W h kg^−1^	250 W kg^−1^	265
TPA-FC CMP			129 F g^−1^ at 0.5 A g^−1^	18 W h kg^−1^	250 W kg^−1^	
TPE-FC CMP			80 F g^−1^ at 0.5 A g^−1^	11 W h kg^−1^	250 W kg^−1^	
FcCMPs/rGO	1 M H_2_SO_4_	Three electrode	470 F g^−1^ (933 mF cm^−3^) at 0.5 A g^−1^	—	—	266
		Two electrode	231 F g^−1^ (238 mF cm^−3^) at 0.5 A g^−1^	8 W h kg^−1^	124 W kg^−1^	
Py-FC-CMP	1 M KOH	Three electrode	272 F g^−1^ at 0.5 A g^−1^	37.75 W h kg^−1^	250 W kg^−1^	267
TBN-FC-CMP			385 F g^−1^ at 0.5 A g^−1^	52.43 W h kg^−1^	250 W kg^−1^	
TBE-FC-CMP			497 F g^−1^ at 0.5 A g^−1^	87.45 W h kg^−1^	250 W kg^−1^	
BP-FC-CMP			608 F g^−1^ at 0.5 A g^−1^	87.45 W h kg^−1^	250 W kg^−1^	

**Ferrocene-based MOFs**						
1-GCE	1 M KOH	Three electrode	446.8 F g^−1^ at 1.2 A g^−1^	Max. 13.70 W h kg^−1^	Max. 11 750.99 W kg^−1^	268

### 4.7. Tetrathiafulvalene (TTF)-based organic materials for supercapacitors

Tetrathiafulvalene (TTF) is an organosulfur compound exhibiting redox-active properties, which are ideal characteristics for electrochemical devices.^270^ In recent years, TTFs have emerged as important building blocks for the fabrication of optoelectronic materials.^271^ Herein, we aim to summarize the application of TTF^270,271^ in the fabrication of supercapacitor devices for energy storage.^272^

**Fig. 55:**
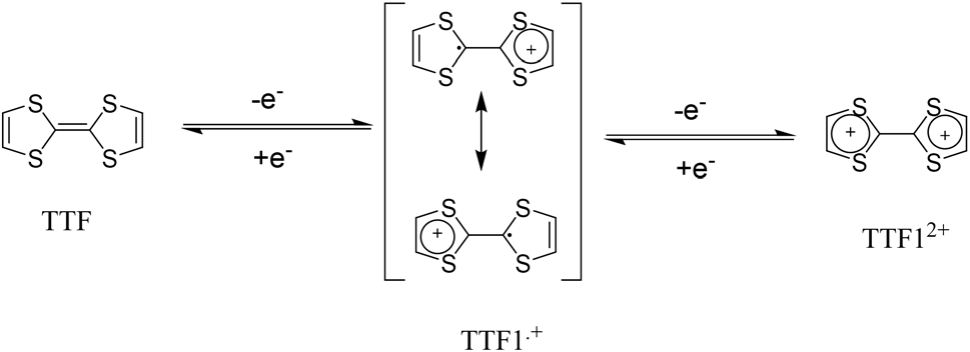
Redox-properties of TTF.

The TTF molecular structure is the classical Weitz type redox-system exhibiting three oxidation states.^273^ As illustrated in Fig. 55, at first, the neutral TTF is converted into the long-term stable radical cation species TTF1^+^*via* one-electron oxidation.^274^ Subsequently, a second oxidation results in the formation of the TTF1^2+^ dication. Both redox-steps are fully reversible and exhibit a lower oxidation potential of 0.37 and 0.74 V *vs.* Ag/AgCl in acetonitrile.^275^ TTF with this reversible redox-behaviour and its stability in both the solution and solid states makes it an attractive candidate for charge-storage applications.^270–275^ In this regard, Zhou and co-workers fabricated three-dimensional sulfur (tetrathiafulvalene)-doped graphene hydrogels (SGHs).^276^ They observed that the presence of TTF plays a key role in the formation of the 3D structure. They confirmed that not only GO assembled into 3D nanostructures but TTF can also be transformed into TTF^+^ and TTF^2+^. The SC in the three-electrode configuration based on SGHs in aqueous 6 M KOH electrolyte solution with a voltage window of −0.9 to 0 V exhibited a *C*_sp_ of 212.5 F g^−1^ at a current density of 0.3 A g^−1^. Alternatively, in the SSC device, the SGH-based electrode displayed a *C*_sp_ of 191.6 F g^−1^ at 0.3 A g^−1^. The SSC device also showed a longer cycling life with 98% retention of its initial *C*_sp_ value after 4000 GCD cycles at a current density of 1 A g^−1^, indicating the excellent performance of the as-fabricated electrode based on TTF.

#### 4.7.1. Tetrathiafulvalene-based polymers for supercapacitors

TTF-based redox-active polymers have been utilized as next-generation electrode materials in battery applications^277^ but our search revealed that these polymers have not been explored for SC applications to the best of our knowledge.

#### 4.7.2. Tetrathiafulvalene-based COFs for supercapacitors

**Fig. 56:**
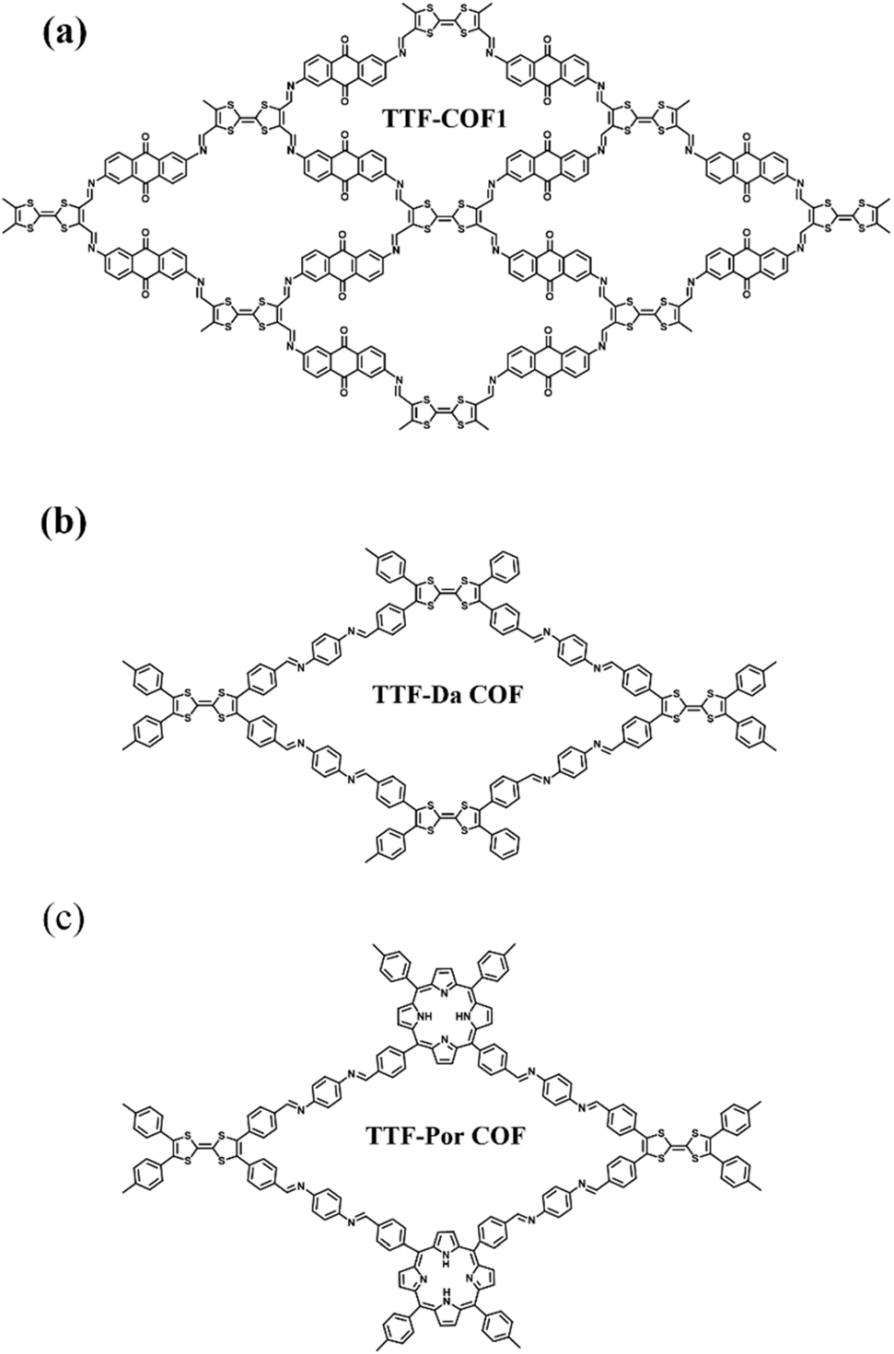
(a–c) Different structures of TTF-based COFs.

Owing to their highly conjugated skeleton, porous structure, and tuneable functionality, electrode materials based on covalent organic frameworks (COFs) have attracted attention from researcher for use in SC applications.^278^ It was observed that due to their lack of redox-active subunits and low conductivity, COFs are considered unsuitable for SC applications.^279^ Thus, to overcome these limitations, Gu and co-workers designed a COF denoted as TTF-COF1 based on the donor tetraformyl-tetrathiafulvalene (TTF-fo) and acceptor 2,6-diaminoanthraquinone (DAQ) and used it for the fabrication of electrode materials (Fig. 56).^280^

The BET analysis of TTF-COF1 exhibited a typical type-II adsorption/desorption isotherm. The calculated SSA based on the BET analysis was 729 m^2^ g^−1^. The pore size of TTF-DAQ was found to be 1.4 nm. The present authors also examined the stability of the COF material after immersing it in organic solvents, boiling water, acids and bases. Based on these experiments, the BET SSA of TTF-COF1 showed no significant changes, implying the structural stability of the electrode. TTF-COF1 based on TTF-fo and DAQ displayed intramolecular charge transfer properties. Moreover, in SC applications at a current density of 1 A g^−1^, the device exhibited a *C*_sp_ of 752 F g^−1^. In contrast, the asymmetric SC device assembled using TTF-COF1 (Fig. 56a) and activated carbon as the cathode and anode, respectively, displayed a *C*_sp_ of 183 F g^−1^ at 1 A g^−1^ with the energy density reaching 57 W h kg^−1^ at a power density of 858 W kg^−1^. Moreover, the ASC device displayed 90% *C*_sp_ retention after 10 000 GCD cycles. The higher performance of TTF-COF1 was ascribed to the conjugated donor–acceptor arrangement in its 2D structure, which enhanced the delocalization of π–electrons, thus increasing the electrical conductivity. It is important to note that the highly porous structure of TTF-COF1 with numerous redox-active sites contributed to the higher pseudocapacitance in the device. In all, the combination of intramolecular charge transfer (ICT) properties and enhanced redox characteristics resulted in a high electrochemical performance by TTF-COF1.

**Fig. 57:**
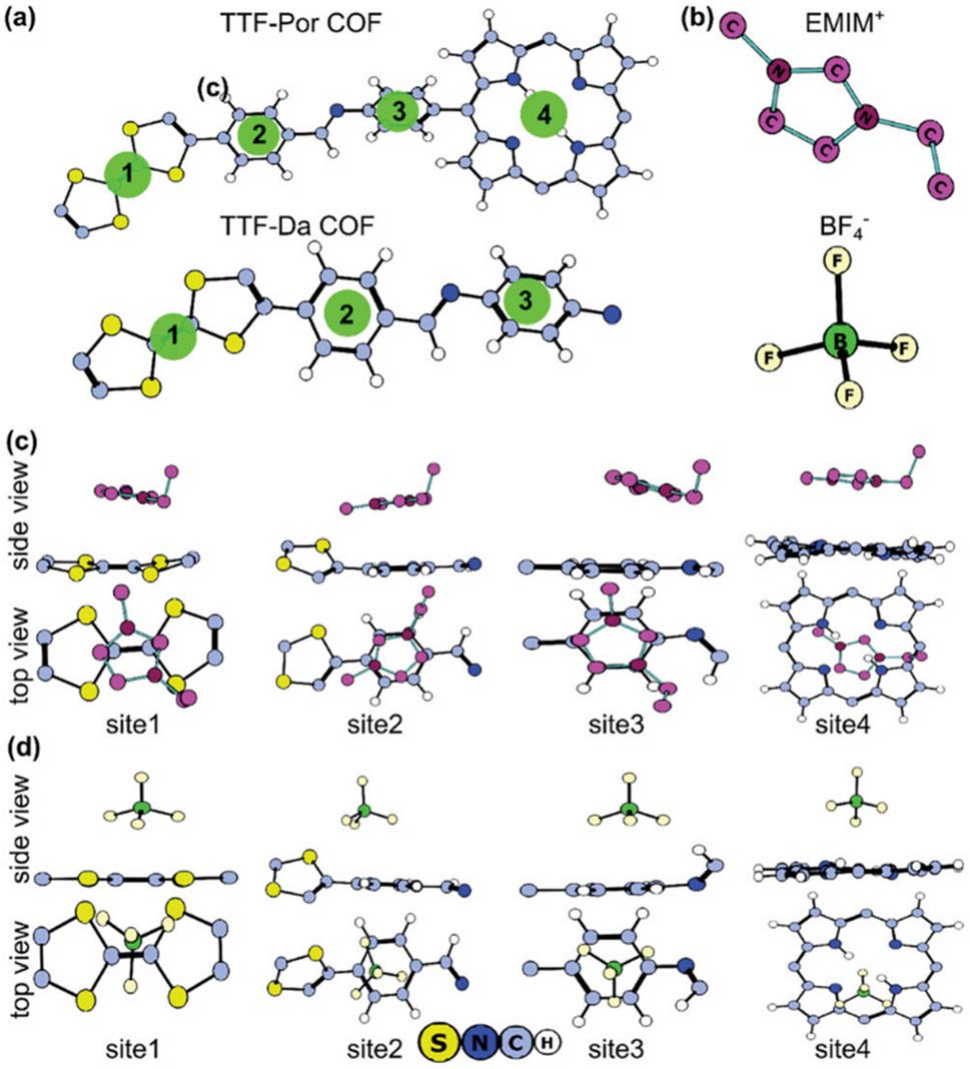
(a) All considered adsorption sites on the TTF-Por COF and TTF-Da COF and (b) EMIM^+^ and BF_4_^−^ optimized geometries. Optimized geometries of adsorbed (c) EMIM^+^ and (d) BF_4_^−^ on all the sites of the TTF-Por COF (viewed from either the side or the top). The atom colors for the COF are elaborated in the figure. H atoms of EMIM^+^ were omitted for clarity. Reproduced from ref. 281 with permission from [John Wiley and Sons], Copyright [2023].

Voort and co-workers reported the preparation of two donor–acceptor-type COFs named TTF-Da (Fig. 56b) and TTF-Por (Fig. 56c) based on TTF and their utilization is SSC applications in the presence of different electrolyte systems.^281^ At 87 K, argon sorption measurements were performed to examine the textural properties of the as-fabricated COF material. The BET analysis of both TTF-Da COF and TTF-Por COF exhibited a type-I isotherm. The BET SSA and total pore volume of TTF-Da COF and TTF-Por COF were found to be 496 m^2^ g^−1^ (pore volume = 0.30 cm^3^ g^−1^) and 424 m^2^ g^−1^ (pore volume = 0.29 cm^3^ g^−1^), respectively. The BET analysis indicated that the present TTF-Da COF and TTF-Por COF materials display great potential for charge storage applications in SC cell configurations. Most of the previously reported COFs were studied in aqueous electrolytes, showing faradaic pseudocapacitor behavior. However, these authors for the first time examined COFs in their pristine form for their charge storage properties and stability by performing the tests in the non-faradaic electrochemically double layer capacitance region. The performance of the TTF-Da and TTF-Por COF-based SSC devices was examined in three different electrolytes including aqueous 1 M Na_2_SO_4_ electrolyte, organic 1 M TEABF_4_ electrolyte in ACN and ionic liquid (IL) EMIMBF_4_ electrolyte.^281^ The GCD profiles of TTF-Da and TTF-Por in a three-electrode system at a lower current density of 0.1–1 A g^−1^ displayed that the specific capacitance in both cases improved with a change in electrolyte, following the order of aqueous < organic < IL.^281^ This could be ascribed to the wider potential window and increased charge difference between the TTF electron donor and porphyrin electron acceptor. Thus, TTF-Por exhibited a *C*_sp_ of 42, 70 and 130 F g^−1^ at a current density of 1 A g^−1^ in aqueous, organic and IL electrolyte, respectively. The COFs and ionic liquid electrolyte showed stronger electrostatic attraction, resulting in the EDLC mechanism. The TTF-Por COF displayed an energy density as high as 58 W h kg^−1^ at the power density of 1 kW kg^−1^. The authors claimed that the TTF-based COF exhibited EDLC behaviour with the best power and energy densities comparable to that of faradaic pseudocapacitive COF electrode materials.^282^ Moreover, they observed that the charge storage properties of these COFs in ionic liquid were dependent on the surface charge density of the donor–acceptor and the micropore size of the COFs. DFT calculations were performed at the PBE-D3 (BJ) level^283^ in the VASP package^284^ to establish the influence of the electron-accepting subunits on the surface charge density properties of the TTF-Por and TTF-Da COFs and their effect on the energy-storage mechanism in an ionic liquid electrolyte. As demonstrated in Fig. 57, the binding of EMIM^+^ and BF_4_^−^ on COFs was studied. According to the DFT calculations, the authors observed that EMIM^+^ binds at the top of the porphyrin ring (Fig. 57, site 4) (adsorption energy of −1.66 eV), whereas in the TTF-Da COF, the benzene subunit (adsorption energy −1.40 eV) (Fig. 57, site 2). The estimated absorption energies of these adsorption are consistent with the experimental results. In the case of BF_4_^−^ adsorption (Fig. 57, site 1) on the TTF-Da COF with a binding energy of −3.94 eV, it is stronger than TTF-Por COF with a binding energy of −3.84 eV.

**Fig. 58:**
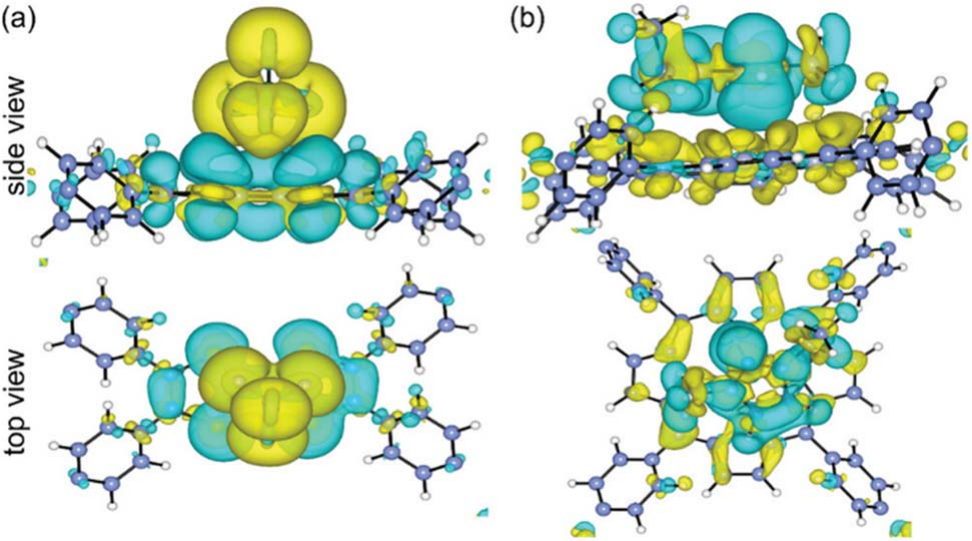
Charge distribution of (a) adsorbed BF_4_^−^ (site 1) and (b) EMIM^+^ (site 4) on the TTF-Por COF. The cyan and yellow isosurfaces (0.001 eÅ^−3^) represent the charge depletion and accumulation, respectively. Reproduced from ref. 281 with permission from [John Wiley and Sons], Copyright [2023].

The charge density distribution of the adsorbed EMIM^+^ and BF_4_^−^ on COFs is illustrated in Fig. 58.^281^ As shown in Fig. 58a, the yellow colored isosurface around BF_4_^−^ displays the charge accumulation, whereas the cyan isosurface on the COF surface shows charge depletion. Fig. 58b shows that charge depletion and accumulation take place on the EMIM^+^ and COF surfaces, respectively.^281^ This offers favorable conditions for the EMIM^+^ cation to adsorb on the COF surface. These results are in agreement with the experimental results, which showed that the adsorption of the EMIM^+^ cation on TTF-Por COF is stronger than the TTF-Da COF. These results were extracted from the Bader charge analysis data, where ≈0.81|*e*| charge transfer takes place between TTF-Por and EMIM^+^ cations and for TTF-Da it decreased to ≈0.72|*e*|.

The theoretical and electrochemical experimental results obtained for TTF-Da and TTF-Por can be utilized as a platform to design novel electrode materials for the future development of EES technology.

#### 4.7.3. Tetrathiafulvalene-based MOFs for supercapacitors

Hybrid organic–inorganic materials such as metal–organic frameworks (MOFs) have been utilized in SC applications due to their ordered porosity, robust framework and fast ion diffusion.^285^ Although TTF based MOFs have been utilized in battery applications their use in SCs have been unexplored on a large scale. In this context, Dai and co-workers designed and prepared three redox-active TTF-based MOFs named [Cu(HL)_2_(bpa)_2_]_*n*_ (1), [Cu(bpe)_2_(H_2_O)_2_]_*n*_·2*n*(HL)·*n*MeOH·*n*H_2_O (2), and [Cu(bpp)_2_(H_2_O)_2_]_*n*_·2*n*(HL) (3) (L = dimethylthio-tetrathiafulvalene-bicarboxylate).^286^ The charge-storage performance of these compounds were examined by means of CV and GCD measurements in the applied voltage window of 0.0 to 0.55 V. At a current density of 1 A g^−1^, MOFs 1, 2 and 3 displayed the *C*_sp_ of 45, 86 and 70 F g^−1^, respectively. These results suggest that 2D MOFs based on the TTF redox-active molecular subunit are beneficial for the delivery of a good specific capacitance in SC applications. The electrochemical results of these MOFs established the structure–property relationship of TTF for use in advanced pseudocapacitor applications.

Tetrathiafulvalene (TTF) represents a very prominent class of redox-active materials in the field of pseudocapacitive electrode materials. A compilation of SC results is displayed in the Table 9. Among the tested small molecules, COF and MOFs, and TTF-COF1^280^ displayed the highest *C*_sp_ of 183 F g^−1^ at 1 A g^−1^, whereas TTF-Por^281^ exhibited excellent energy density of about 58 W h kg^−1^. An improvement regarding the energy density was achieved by the application of TTF in polymers and COFs.^280,283^ Interestingly, the presence of TTF in the structure of these polymers and COFs significantly influenced the faradaic reversible redox-process in SCs.

**Table 9:** Comparison of the electrochemical properties of tetrathiafulvalene (TTF)-based small molecules, polymers, covalent organic frameworks (COFs), and metal organic frameworks (MOFs)

Compound code	Electrolyte	Type of working electrode	Specific capacitance (*C*_sp_)	Energy density (ED)	Power density (PD)	Ref.
**TTF based small molecules**						
SGHs	6 M KOH	Three electrode	212.5 F g^−1^ at 0.3 A g^−1^	—	—	276
		Two-electrode SSC	191.6 F g^−1^ at 0.3 A g^−1^	—	—	

**TTF based polymers and COFs**						
TTF-COF1	3 M KOH	Three electrode	752 F g^−1^ at 1 A g^−1^	—	—	280
		Two-electrode ASC	183 F g^−1^ at 1 A g^−1^	57 W h kg^−1^	858 W kg^−1^	
TTF-Da	EMIMBF_4_ (ionic liq.)	Two-electrode SSC	100 F g^−1^ at 1 A g^−1^	58 W h kg^−1^	1 kW kg^−1^	281
TTF-Por	EMIMBF_4_ (ionic liq.)		130 F g^−1^ at 1 A g^−1^	58 W h kg^−1^	1 kW kg^−1^	

**TTF-based MOFs**						
[Cu(HL)_2_(bpa)_2_]_*n*_ (1)	6.0 M KOH	Three electrode	45 F g^−1^ at 1.0 A g^−1^	—	—	286
[Cu(bpe)_2_(H_2_O)_2_]_*n*_·2*n*(HL)·*n*MeOH·*n*H_2_O (2)			86 F g^−1^ at 1.0 A g^−1^	—	—	
[Cu(bpp)_2_(H_2_O)_2_]_*n*_·2*n*(HL) (3)			70 F g^−1^ at 1.0 A g^−1^	—	—	

### 4.8. Viologen-based organic materials for supercapacitors

**Fig. 59:**
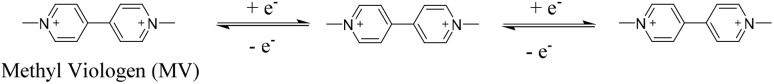
Redox-properties of methyl viologen (MV).

Viologen is an organic heterocyclic redox compound exhibiting two one-electron reduction steps (Fig. 59).^287^ Two quaternary pyridinium rings are present in the viologen molecular skeleton. The outstanding redox-behaviour of viologen makes it a fascinating molecular architecture for different optoelectronic applications such as photochromism, electrochromism, electrocatalysis and energy storage.^288^ Viologen with cationic charges and redox-behaviour is highly impressive to deliver excellent charge storage performances in energy storage systems.^287,288*d*^

**Fig. 60:**
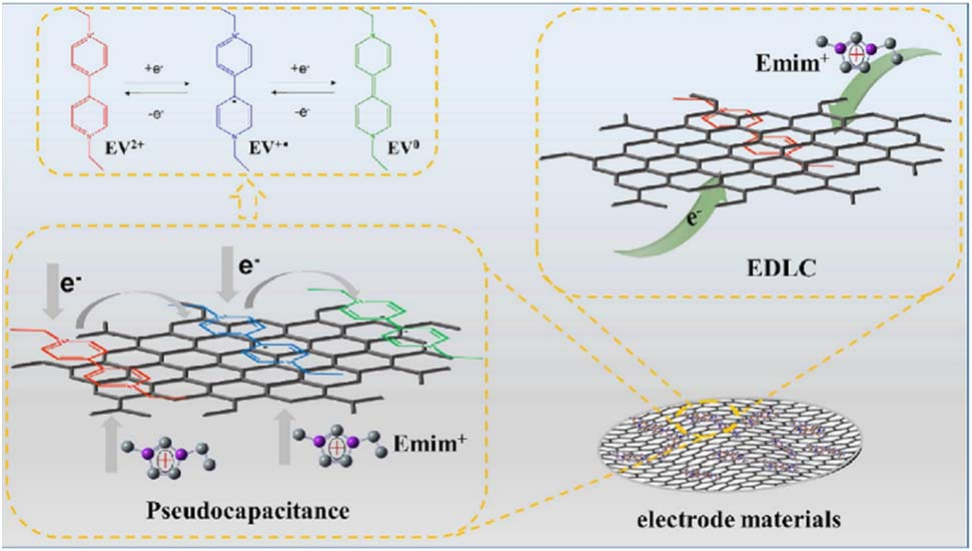
Charge-storage mechanisms of EV_20_-RGO on the negative electrodes. Reproduced from ref. 292 with permission from [Elsevier], Copyright [2023].

Velayutham and co-workers demonstrated the utilization of a redox-active viologen (1,1′-diethyl-4,4′-bipyridinium bromide)-based electrolyte in combination with 1 M H_2_SO_4_ aqueous electrolyte for SC applications.^289^ The presence of viologen improved the performance of the cathode and anode simultaneously *via* its redox behaviour. The AC//AC SSC device based on activated carbon as the cathode and anode in the presence of 1.0 M H_2_SO_4_–0.03 M viologen electrolyte delivered a *C*_sp_ of 408.0 F g^−1^ at a current density of 0.25 A g^−1^, which is higher than that of AC//AC (*C*_sp_ = 254 F g^−1^ at 0.25 A g^−1^) in the presence of only 1.0 M H_2_SO_4_–0.03 M N_222_Br. This could be ascribed to the continuous increase in the pseudocapacitive contribution from bipyridinium cations, which enhanced the specific capacitance of the device. Herein, the bipyridinium cations adsorb on the carbon-based materials *via* π–π stacking interactions between the viologen electrolyte and electrodes, which resulted in a higher concentration of bipyridinium cations at the activated carbon-based electrode surface.^290^ Moreover, the AC//AC SSC device also exhibited a specific energy as high as 23.0 W h kg^−1^ at 0.25 A g^−1^. In the case of the viologen-mediated AC//AC device, the specific capacitance continuously increased in the GCD cycles with a 30% increment over 1000 cycles. Kim and co-workers utilized aniline-substituted viologen electrolytes for the fabrication of SCs. The aniline monomer (AM-viologen), dimer (AD-viologen) and trimer (At-viologen) exhibited the areal capacitance of 0.82, 5.81 and 2.17 mF cm^−2^, respectively.^291^ AD-viologen displayed the energy density of 1.13 μW h/cm^2^ and power density of 62.03 μW cm^−2^ at 0.1 mA cm^−2^. Moreover, the cycling stability of the aniline monomer-, aniline dimer-, and aniline trimer-substituted viologens after 1000 cycles was found to be 98.6%, 90.8%, and 20.3%, respectively. According to this investigation, they established that the aniline dimer-functionalized viologen electrolyte displayed a good performance compared to the mono- and tri-aniline-substituted derivatives. Zhuo and co-workers reported the preparation of an ethyl-viologen (EV)-functionalized reduced graphene oxide (RGO) material named EC-RGO composite and its utilization in SC applications.^292^ The SSA and pore size distribution of EV_20_-RGO and RGO were examined by means of BET analysis using N_2_ adsorption/desorption isotherms. According to the IUPAC classification, both electrode materials displayed type-IV adsorption/desorption isotherms. The presence of a hysteresis loop indicates that the material possessed mesopores in its structure. In addition, the presence of macropores was confirmed by means of the rapid upward trend in high *P*/*P*_0_ (>0.95). To store more electrolyte, the macropores in the EV_20_-RGO and RGO structures provided ion buffer compartments. Consequently, the as-fabricated RGO, EV_10_-RGO, EV_20_-RGO, EV_30_-RGO and EV_40_-RGO exhibited the SSA of 339.7, 80.4, 97.9, 69.2 and 42.5 m^2^ g^−1^, respectively, indicating the potential of these materials for enhancing the charge storage properties. TGO and EC-RGO in EmimBF_4_ ionic liquid electrolyte were tested for SCs. At a current density of 1 A g^−1^, RGO, EV_10_-RGO, EV_20_-RGO, EV_30_-RGO, and EV_40_-RGO displayed the specific capacitance of 132.2 F g^−1^, 198.2 F g^−1^, 215.7 F g^−1^, 190.9 F g^−1^, and 176 F g^−1^, respectively.^292^ It was observed that EV_20_-RGO delivered a 1.6-times higher *C*_sp_ compared to the pristine RGO. The highest *C*_sp_ of 222.7 F g^−1^ at 0.5 A g^−1^ was observed for EV_20_-RGO. The EV^2+^ in EmimBF_4_ showed a faradaic reversible redox process with low internal resistance, and also a good rate capability. Thus, the EV_20_-RGO//AC ASC device exhibited the *C*_sp_ of 36 F g^−1^ at 0.5 A g^−1^. Moreover, the ASC device exhibited the specific energy of 64.1 W h kg^−1^ at a power density of 888 W kg^−1^. The plausible pseudocapacitive mechanism exhibited by EV_20_-RGO in the GCD process is illustrated in Fig. 60.^292^ During the faradaic reversible redox process, the active EV^2+^ species undergoes two one-electron reactions (see Fig. 59 also). In addition, during the charging/discharging processes, Emim^+^ acts as a counterion. It is also found that ethyl viologen cations with RGO showed electrostatic, π–π stacking and cation–π interactions. Therefore, the higher electrochemical performance could be attributed to the synergic contributions from EDLC and pseudocapacitance.

**Fig. 61:**
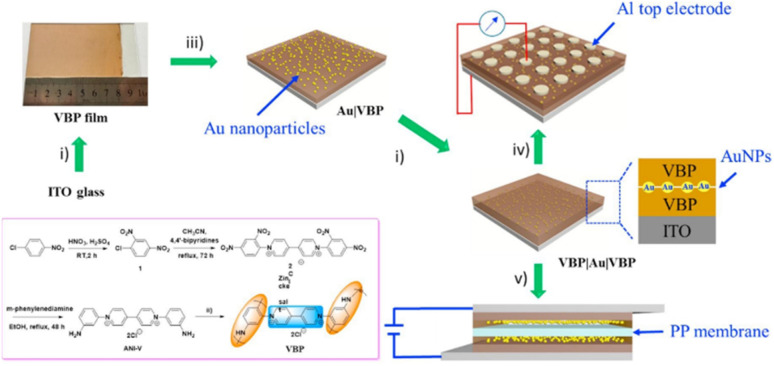
Schematic presentation of the fabrication of the VBP|Au|VBP hetero-film and device. Reproduced from ref. 293 with permission from [Elsevier], Copyright [2017].

Sandwich-like multi-layered films have been used as active materials to fabricate energy storage devices. In this regard, Zhuang and co-workers synthesized a viologen-bridged polyaniline (VBP) film, which was utilized to prepared gold nanoparticles (AuNPs) to fabricate hetero-films (VBP|Au|VBP) (Fig. 61) as active electrode materials.^293^ The VBP|Au|VBP-based SC delivered a volumetric capacitance of 6.22 F cm^−3^. In the case of the VBP|Au|VBP-based SC device, it achieved a volumetric energy density of 2.24 mW h cm^−3^ at a power density of 13.98 mW cm^−3^. Thus, the rational design of these sandwiched films offers a new way for the fabrication of solid-state SC devices.

**Fig. 62:**
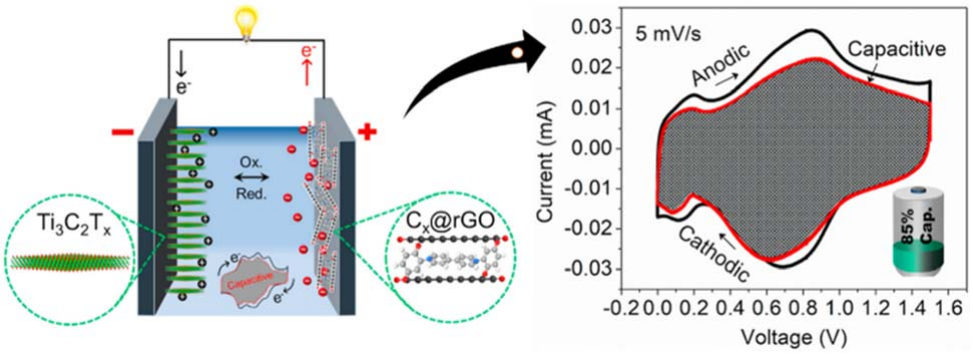
ASC device architecture based on quinone-functionalized viologen (C_*x*_)@rGO as the positive electrode and Ti_3_C_2_T_*x*_ MXene as the negative electrode and the energy storage mechanism. Reproduced from ref. 294 with permission from [Elsevier], Copyright [2020].

Boota and co-workers synthesized quinone-functionalized viologen and in combination with reduced graphene oxide (rGO) was utilized for the fabrication of the C_*x*_@rGO electrode material.^294^ The as-fabricated C_3_@rGO as the positive and Ti_3_C_2_T_*x*_ MXene as the negative electrodes were used for the construction of an asymmetric SC device (Fig. 62).^294^ The system acted as a multi-electron redox asymmetric pseudocapacitor. The ASC device in the applied potential window of 1.5 V in 3 M H_2_SO_4_ electrolyte solution displayed the *C*_sp_ of 64 F g^−1^ at a scan rate of 10 mV s^−1^. It also exhibited a good specific energy of ∼20 W h kg^−1^ with 80% capacitance retention after 10 000 GCD cycles. The good electrochemical behaviour of the C_3_@rGO//Ti_3_C_2_T_*x*_ MXene ASC device (Fig. 62) can be ascribed to the matching redox characteristics of its electrodes, balanced mass, electronic conductivity of the rGO and MXene materials, higher ionic diffusion in the H_2_SO_4_ electrolyte and synergistic effect of both redox-active electrodes at various potentials in similar electrolyte solutions.^295^

**Fig. 63:**
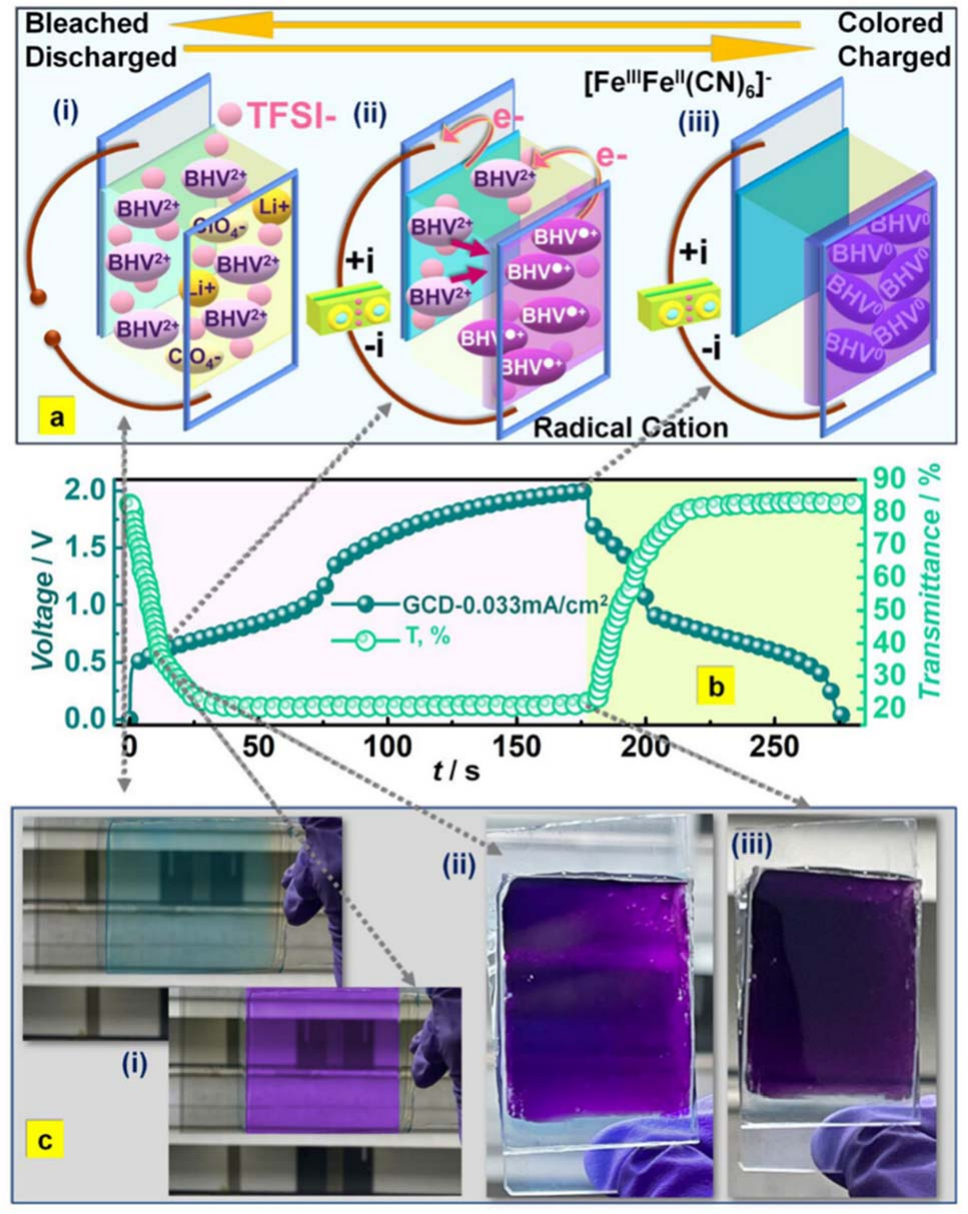
(a) Schematic representing the reversible change in the composition of the BHV//PB ESC during GCD measurements: (i) discharged, (ii) semi-charged, and (iii) fully charged states. (b) Concomitant transmittance (at 550 nm) *vs.* time and voltage *vs.* time profiles of BHV//PB ESC. (c) Photographs of the BHV//PB ESC corresponding to (i–iii) states in (a). Reproduced from ref. 296 with permission from [the American Chemical Society], Copyright [2023].

Recently, Deepa and co-workers fabricated an electrochromic supercapacitor (ESC) (Fig. 63) based on benzyl hexenyl viologen (BHV) as the anode and Prussian blue (PB) as the cathode, which in the applied potential voltage window of 2 V displayed a *C*_sp_ of 1.67 mF cm^−2^ or 67 F g^−1^ at a current density of 0.03 mA cm^−2^.^296^ The device also yielded an energy of 37.2 W h kg^−1^ at a power density of 6.7 W kg^−1^. The cycling stability for the BHV//PB ESC after 5000 GCD cycles was found to be 85% without major optical modulation loss. The device color was pale blue (Fig. 63a(i)), which could be attributed to the color of the PB film. Fig. 63a(ii) shows the charging process of BHV//PB ESC and the purple color of the film. Further oxidation of the PB film resulted in a darker purple color. The variation in transmittance of the device at 550 nm as a function of time and changing voltage is demonstrated in Fig. 63b. Thus, the charging and discharging process can be monitored by means of the color change in the device (Fig. 63c).^296^ The pale blue color of the PB film indicates the discharging state of the device, whereas pale purple suggests the semi-charged state and the discharged state is confirmed by the change in color to deep purple (Fig. 63c). The three charging and discharging states and their color changes are illustrated in Fig. 63a and c, respectively. The authors claimed that this BHV//PB ESC device can be utilized to construct next-generation electronic devices.^296^

Zhuang *et al.* reported the synthesis of triarylamine-functionalized symmetric viologen molecular architectures such as TPA-bpy and CZ-bpy for electrochromic supercapacitors.^297^ The as-fabricated ESC device based on TPA-bpy in the applied potential voltage window of 2.0 V showed a change in color from purple to yellow during the charge storage process. Moreover, the device displayed excellent cycling stability of 90% after 6000 cycles. Polyoxometalates (POMs) have emerged as promising candidates as pseudo-capacitive electrode materials due to their rich reversible multi-electron redox behavior.^298^ To formulate high-performance supercapacitor devices, Wang and co-workers synthesized three Keggin-based Cu/Ni viologen complexes, [Cu_2_(L_1_)_4_(L_2_)(H_2_O)_4_(Si/GeMo_12_O_40_)_2_]·8H_2_O (1 = Si and 2 = Ge) and [Ni(L_1_)_2_(L_2_)(H_2_O)_2_(SiMo_12_O_40_)]·4H_2_O (3) (*L*_1_ = 1-(4-formyl-benzyl)-[4,4′]bipyridinyl-1-ium and *L*_2_ = 4,4′-bipyridinyl).^299^ The BET analysis of complexes 1–5 was performed to estimate their SSA. Complex 1 displayed the highest BET SSA of ∼1.6389 m^2^ g^−1^ compared to complexes 2 (1.3707 m^2^ g^−1^), 3 (1.337 m^2^ g^−1^), 4 (0.3733 m^2^ g^−1^) and 5 (0.1331 m^2^ g^−1^). It is also noticeable that complex 1 exhibited a smaller pore size distribution of ≈2.97 nm. The BET results provide the basis for the high charge storage properties of complex 1. They utilized complexes 1, 2 and 3 as high-capacity negative electrodes for the fabrication of SC devices. Viologen-based complexes 1, 2 and 3 at a current density of 2 A g^−1^ exhibited the *C*_sp_ of 1618.4 F g^−1^, 1457.6 F g^−1^ and 1421.6 F g^−1^, respectively. Moreover, at a current density of 1 A g^−1^, the 1//activated carbon (AC) ASC device showed a significant specific energy density of 26.82 W h kg^−1^ at a power density of 600 W kg^−1^. At a higher current density of 10 A g^−1^ and higher power density of 6000 W kg^−1^, the energy density was maintained at 9.39 W h kg^−1^. The cycling life of the initial *C*_sp_ was found to be 78.8% after 10 000 cycles. Furthermore, the ASC device was successfully used for the illumination of a red diode, demonstrating the importance of complex 1 in real-world practical applications. Thus, the present results indicate the importance of viologen in POM-based pseudocapacitor electrode materials. It is noticeable that the viologen subunit in the electrode material enhanced the intrinsic conductivity and provided the basis for the excellent acceptor to display a hydrogen bond network.

Thus, the above-mentioned examples demonstrate the importance of viologen as an electrolyte and electrode material in supercapacitor applications.

#### 4.8.1. Viologen-based polymers for supercapacitors

**Fig. 64:**
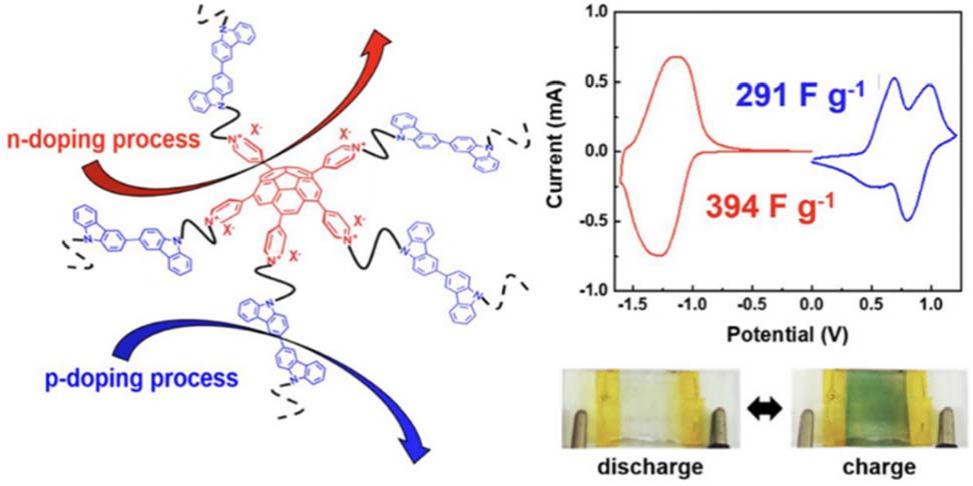
Structure of ambipolar polymers and their potential window and color changes during the charging and discharging states in the visible region. Reproduced from ref. 302 with permission from [the American Chemical Society], Copyright [2022].

The combination of p- and n-type subunits is necessary to construct ambipolar polymers with electrochromic properties. These p/n-type moieties can accept positive and negative charges during the faradaic reversible redox process, respectively, and are suitable for SC applications.^300^ In case connection, viologen with redox-active properties acts as an n-type material in battery and supercapacitor applications.^301^ Luo and co-workers reported the electrochemical polymerization of carbazole-substituted corannulene-extended viologen (Fig. 64) in the mixed solvent of dichloromethane/acetonitrile using tetrabutylammonium hexafluorophosphate (TBAPF_6_) as the electrolyte by scanning from 0.0 to 1.4 V *vs.* Ag/Ag^+^.^302^ The as-fabricated polymers PCP2, PCP4, PCP6, and PCP8 (Fig. 64)^302^ displayed *C*_sp_ of 234, 291, 142, and 69 F g^−1^, respectively, in the p-doping region. In contrast, in the n-doping region, the specific capacitance of 406, 394, 237, and 34 F g^−1^ was observed for PCP2, PCP4, PCP6, and PCP8, respectively. According to these specific capacitance results, it was found that n-type doping is impressive. The PCP4 polymer with a butyl chain linker displayed the highest *C*_sp_ in both types of doping. The ambipolar polymer in an electrochromic SC device showed color in the neutral and doped states. The chain length of the polymers also affected the optical contrast and *C*_sp_.

**Fig. 65:**
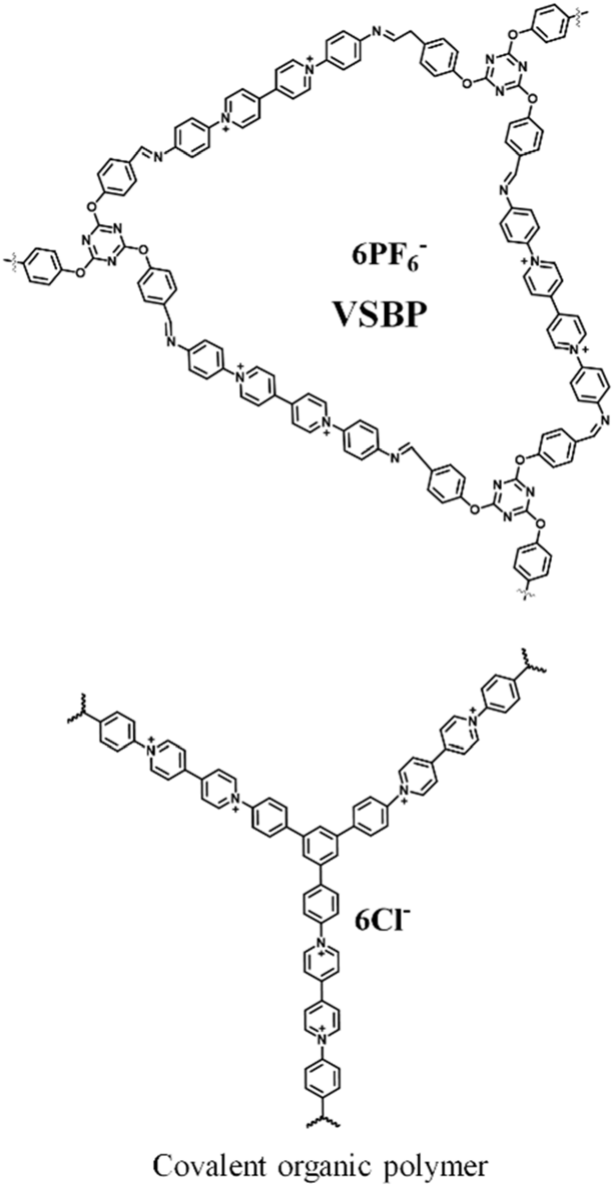
Structures of the hyperbranched viologen-containing Schiff base polymer (VSBP) and covalent organic polymers.

Mareeswaran and co-workers a synthesized viologen-bearing Schiff base polymer denoted as VSBP (Fig. 65) and examined its SC performance.^303^ The VSBP-modified nickel foam (NF) electrode at 0.5 A g^−1^ displayed a *C*_sp_ of 256 F g^−1^ and 87% was retained after increasing the current density to 10 A g^−1^. Moreover, at 0.5 A g^−1^, after 3000 GCD cycles, the three-electrode device retained 95% of its initial *C*_sp_ value. The VSBP/NF//VSBP/NF SSC device showed a specific energy as high as 17.02 W h kg^−1^ at a power density of 816 W kg^−1^. Furthermore, the VSBP/NF//VSBP/NF cell configuration displayed the *C*_sp_ retention of 90.6% at 1 A g^−1^ over 5000 GCD cycles. Herein, VSBP showed pseudocapacitive behaviour due to the presence of abundant redox-active subunits in the polymer, which can provide the basis for the design and synthesis of new viologen-based polymers for pseudocapacitors to resolve the energy crisis.

Kathiresan and co-workers prepared two covalent organic polymers, COP-1 and COP-2 (Fig. 65), in different solvents using viologen as one of the redox-active subunits.^304^ The morphology of COP-1 and COP-2 was found to be hollow spheres and hollow tubes, respectively. In the three-electrode SC cell configuration in 1 M H_2_SO_4_ aqueous electrolyte solution, COP-2 displayed the *C*_sp_ of 604 F g^−1^ at a current density of 2 A g^−1^. In contrast, the COP-2-based SSC device delivered the *C*_sp_ of 404 F g^−1^ at 0.5 A g^−1^. The specific energy reached 14 W h kg^−1^ at a power density of 1848 W kg^−1^. In the SSC device, an impressive cycling stability with 100% *C*_sp_ retention was observed after 50 000 cycles. The covalent polymeric materials unveiled the electrochemical performance of viologen for creating efficient SC device architectures with futuristic charge-storage and electronic applications.

A doubly positively charged cation is the specific feature of viologen redox-active materials. The application of viologen-based electrodes in pseudocapacitors is summarized in Table 10. The n-type viologen electrode material EV_20_-RGO^292^ in a two-electrode ASC device exhibited the outstanding energy density of about 64.1 W h kg^−1^ at a power density of 888 W kg^−1^, whereas the [Cu_2_(L_1_)_4_(L_2_)(H_2_O)_4_(SiMo_12_O_40_)_2_]·8H_2_O (1)^299^ material in a three-electrode SC system displayed the highest *C*_sp_ of 1618.4 F g^−1^ at 2 A g^−1^. Viologen was also embedded in polymers. The PSC device based on PCP4 (ref. 302) displayed the specific capacitance of 291 F g^−1^ (*C*_p_) and 394 F g^−1^ (*C*_n_) with the maximum energy density of 105 W h kg^−1^. The charge-storage results revealed the superior specific capacitance and energy density of the electrodes based on viologen small molecules and polymeric materials.

**Table 10:** Comparison of the electrochemical properties of viologen-based small molecules and polymers

Compound code	Electrolyte	Type of working electrode	Specific capacitance (*C*_sp_)	Energy density (ED)	Power density (PD)	Ref.
**Viologen-based small molecules**						
AC//AC	1.0 M H_2_SO_4_–0.03 M viologen	Two electrode SSC	408.0 at 0.25 A g^−1^	23.0 W h kg^−1^ at 0.25 A g^−1^	Not specified in related ref.	289
	1.0 M H_2_SO_4_–0.03 M N_222_Br		254.0 at 0.25 A g^−1^	14.3 W h kg^−1^ at 0.25 A g^−1^		
ITO	AM-viologen	Three electrode	0.82 mF cm^−2^ 0.04 mA cm^−2^	1.13 μW h cm^−2^ at 0.1 mA cm^−2^	62.03 μW cm^−2^ at 0.1 mA cm^−2^	291
	AD-viologen		5.81 mF cm^−2^ 0.04 mA cm^−2^			
	AT-viologen		2.17 mF cm^−2^ 0.04 mA cm^−2^			
RGO	EmimBF_4_ ionic liquid electrolyte	Three electrode	132.2 F g^−1^ at 1 A g^−1^	—	—	292
EV-RGO			198.2 F g^−1^ at 1 A g^−1^	—	—	
EV_10_-RGO			215.7 F g^−1^ at 1 A g^−1^	—	—	
EV_20_-RGO			190.9 F g^−1^ at 1 A g^−1^	—	—	
EV_30_-RGO			176 F g^−1^ at 1 A g^−1^	—	—	
EV_20_-RGO	EmimBF_4_ ionic liquid electrolyte	Two-electrode ASC	36 F g^−1^ at 0.5 A g^−1^	64.1 W h kg^−1^	888 W kg^−1^	
VBP|Au|VBP	PVA/H_3_PO_4_ gel		6.22 F cm^−3^	2.24 mW h cm^−3^	13.98 mW cm^−3^	293
C_*x*_@rGO	3 M H_2_SO_4_	Two-electrode ASC	64 F g^−1^ at 10 mV s^−1^ scan	∼20 W h kg^−1^	480 W kg^−1^	294
BHV	LiClO_4_/PC	BHV//PB electrochromic SC	1.67 mF cm^−2^ (67 F g^−1^) at 0.03 mA cm^−2^	Max. 37.2 W h kg^−1^	Max. 6.7 W kg^−1^	296
TPA-bpy	(PMMA + LiClO_4_+ propylene carbonate	Solid-state electrochromic SC	1.25 mF cm^−2^ at 0.01 mA cm^−2^	0.59 μW h cm^−2^	9.21 μW cm^−2^	297
[Cu_2_(L_1_)_4_(L_2_)(H_2_O)_4_(SiMo_12_O_40_)_2_]·8H_2_O (1)	0.5 M H_2_SO_4_	Three electrode	1618.4 F g^−1^ at 2 A g^−1^	—	—	299
[Cu_2_(L_1_)_4_(L_2_)(H_2_O)_4_GeMo_12_O_40_)_2_]·8H_2_O (2)			1457.6 F g^−1^ at 2 A g^−1^	—	—	
[Ni(L_1_)_2_(L_2_)(H_2_O)_2_(SiMo_12_O_40_)]·4H_2_O (3)			1421.6 F g^−1^ at 2 A g^−1^	—	—	
[Cu_2_(L_1_)_4_(L_2_)(H_2_O)_4_(SiMo_12_O_40_)_2_]·8H_2_O (1)		Two-electrode ASC	134.1 F g^−1^ at 1 A g^−1^	26.82 W h kg^−1^ at 1 A g^−1^	600 W kg^−1^ at 1 A g^−1^	

**Viologen-based polymers**						
PCP2	TBAPF_6_	Pseudocapacitors	234 F g^−1^ (*C*_p_)	93 W h kg^−1^	—	302
			394 F g^−1^ (*C*_n_)			
PCP4			291 F g^−1^ (*C*_p_)	105 W h kg^−1^	—	
			394 F g^−1^ (*C*_n_)			
PCP6			142 F g^−1^ (*C*_p_)	51 W h kg^−1^	—	
			237 F g^−1^ (*C*_n_)			
PCP8			69 F g^−1^ (*C*_p_)	21 W h kg^−1^	—	
			34 F g^−1^ (*C*_n_)			
VSBP/nickel foam (NF)	3 M KOH	Three electrode	256 F g^−1^ at 0.5 A g^−1^	—	—	303
		Two-electrode SSC	45 F g^−1^ at 1 A g^−1^	17.02 W h kg^−1^	816 W kg^−1^	
COP2	1 M H_2_SO_4_	Three electrode	604 F g^−1^ at 2 A g^−1^			304
		Two electrode	404 F g^−1^ at 0.5 A g^−1^	14 W h kg^−1^	1848 W kg^−1^	

## 5. Discussion

### 5.1. Merits of organic electrode materials

Herein, the compilation of the application of organic electrode materials in the fabrication of supercapacitor cell configurations is an important phase of research. To fulfill consumer demands in the fast-expanding field of wearable electronics, electric vehicles and grid integration,^305^ the fabrication of appropriate electrode materials from renewable resources has attracted interest from researchers over the past few decades.^306^ In this case, the design of organic small molecules, polymers, covalent organic frameworks and metal organic frameworks as alternative electrode materials to traditional transition metal-oxide-based electrode materials has grown tremendously. Organic electrode materials exhibit several advantages such as resource sustainability, tunability, low cost, excellent stability, high structural flexibility, recyclability and low toxicity.^307^ It is noticeable that their higher flexibility can provide the basis for faster ion diffusion. Moreover, most organic electrode material-based SCs involve faradaic reversible redox-reactions to store charge. The manipulation of the organic molecular structure with donor and acceptor subunits is helpful to widen the operational potential window of SCs. This can enhance the energy density of the device, resulting in excellent electrochemical properties. These characteristics make organic electrode materials attractive for higher energy density. Therefore, these materials possess great potential to be utilized in next-generation supercapacitor technologies.^308^ We hope that this review will provide an invaluable contribution to the development of organic electrode materials for next-generation pseudocapacitors and help to open the door to potential electrical energy storage applications.

## 6. Conclusions and future perspectives

In this review article, we presented the limitations of traditional inorganic electrode materials and the requirement of redox-active organic electrode materials for pseudocapacitor applications. To expedite the commercialization of redox-active small organic molecule-, polymer-, COF- and MOF-based PSC electrode materials, it is of utmost importance to focus on research for their advancement. In conclusion, considering the challenges in the design of new organic electrode materials, developing cost-effective materials will aid in the advancement of EES technology. The utilization of these electrode materials in EES technology has the potential to enhance and facilitate modern electronic applications. In addition, the exploration of SCs with these redox-active electrode materials is anticipated to play a pivotal role in next-generation pseudocapacitor applications. The development of cutting-edge electrode fabrication methods and technologies will significantly contribute to the aerospace and military fields.

To achieve high-performance EES, the selection of redox-active organic molecules is crucial. OEMs for an effective potential voltage window in SCs need to be designed based on donor and acceptor components. This can be achieved by theoretical calculations of the molecular scaffolds. Also, the voltage window characteristics of the SC cell configuration need to be adjusted properly to exhibit a higher energy density. These PSCs will be more applicable in modern electronic applications. In the future, research should be directed towards the design and development of redox-active organic electrode materials that can specifically deliver a higher specific capacitance, higher energy and power densities and higher cycling stability. Further research work should be focused on the fabrication of more stable organic materials, enabling a consistent improvement in the performance of SCs. Moreover, another avenue of research could be the industrial-scale production of these redox-active molecules. Finally, the performance of SCs can be enhanced by improving the reversible redox-properties of organic electrode materials. Herein, we anticipate that this presentation on organic-electrode materials in their various forms will pave the way for researchers to enjoy this field and contribute more aggressively to the enrichment of SCs.

## Author contributions

Both authors conceived the outline of the manuscript. Sid. Bhosale wrote the original draft of the manuscript. Shesh. Bhosale revised and supervised the manuscript.

## Conflicts of interest

There are no conflicts to declare.

## Data Availability

No primary research results, software or code have been included and no new data were generated or analysed as part of this review.
